# Clinical efficacy of Bupleurum inula flower soup for immune damage intervention in Hashimoto’s thyroiditis: A placebo-controlled randomized trial

**DOI:** 10.3389/fphar.2022.1049618

**Published:** 2022-11-24

**Authors:** Xiangfei Meng, Shiyi Liu, Xiumin Deng, Xintong Li, Jia Lei, Hongye Jiang, Mengyao Liu, Ning Zhang, Shiwei Liu

**Affiliations:** ^1^ Department of Endocrinology and Nephrology, Wangjing Hospital, China Academy of Chinese Medical Sciences, Beijing, China; ^2^ Department of Endocrinology, Tianjin Binhai New Area Hospital of Traditional Chinese Medicine, Tianjin, China; ^3^ Geriatrics Department, Pinggu Hospital, Beijing Hospital of Traditional Chinese Medicine, Beijing, China; ^4^ Medical Department, The Affiliated Hospital of Northwest University, Shanxi, China; ^5^ Graduate School of Beijing University of Chinese Medicine, Beijing, China; ^6^ Department of Oncology, Hospital of Chinese Medicine of Chaoyang District, Beijing, China

**Keywords:** Hashimoto’s thyroiditis, immune damage, thyroid autoantibody, quality of life, traditional Chinese medicine

## Abstract

**Background:** Antibody-mediated humoral immune response is involved in the damage process in Hashimoto’s thyroiditis (HT). Although the traditional Chinese medicine (TCM) formula bupleurum inula flower soup (BIFS) is often used in HT treatment, it has not been evaluated through high-quality clinical research. Rigorously designed randomized, double-blind, prospective clinical studies are urgently needed to evaluate BIFS for intervening in the HT immune damage process, and to improve clinical prognosis and patient quality of life.

**Methods:** A prospective randomized, double-blind, placebo-controlled trial was used to evaluate the efficacy of BIFS. Fifty participants diagnosed with HT with hypothyroidism were randomly assigned at a 1:1 ratio to the BIFS (levothyroxine with BIFS) or control (levothyroxine with placebo) group. Participants received 8 weeks of treatment and were followed for 24 weeks. They were monitored for: levels of thyroid peroxidase antibody (TPOAb), thyroglobulin antibody (TgAb), and thyroid stimulating hormone (TSH); scores for depression, anxiety, and health-related quality of life (HRQoL); thyroid volume; safety indicators including routine blood tests, liver and kidney functions, and electrocardiogram; and levothyroxine dose.

**Results:** Forty-eight participants completed the study and were included in the final analysis. At baseline, there were no significant between-group differences in the observed indicators (*p* > 0.05). Post-treatment, compared with the control group, the BIFS group had significantly lower levels of TPOAb (275.77 ± 132.98 vs. 441.78 ± 195.50, *p* = 0.001), TgAb (385.92 ± 281.91 vs. 596.17 ± 282.26, *p* = 0.013), and TSH (6.57 ± 3.73 vs. 9.63 ± 5.34, *p* = 0.001). Compared with the control group, the BIFS group’s scores improved significantly for depression (47.00 ± 5.12 vs. 51.04 ± 3.22, *p* = 0.002), anxiety (43.21 ± 4.22 vs. 48.08 ± 2.81, *p* = 0.005), and HRQoL physical (62.08 ± 5.97 vs. 57.96 ± 4.71, *p* = 0.011) and psychological (60.17 ± 5.94 vs. 55.75 ± 7.09, *p* = 0.024) subscores. At 24-week follow-up, levothyroxine combined with TCM allowed a significantly reduced levothyroxine dose (0.58 ± 0.43 vs. 1.02 ± 0.45, *p* = 0.001). The post-treatment clinical efficacy rates differed significantly (*p* = 0.03), with 75% (18/24) for the BIFS group and 46% (11/24) for the control group. There were no significant between-group differences in thyroid volume or safety indicators after eight treatment weeks or at the 24-week follow-up (*p* > 0.05).

**Conclusion:** The TCM BIFS can effectively reduce thyroid titer, relieve clinical and emotional symptoms, and improve HRQoL in patients with HT.

**Clinical Trial Registration:** https://www.chictr.org.cn/, identifier ChiCTR1900020987

## Introduction

Hashimoto thyroiditis (HT) is an autoimmune thyroid disease caused by immune dysregulation, also known as chronic lymphocytic thyroiditis, which leads to immune damage to thyroid cells *via* T cell- and antibody-mediated immune processes. The age range of highest incidence of HT is 30–50 years, and its incidence in females is 10 times that of males (Ahmed et al., 2012; Wu and Du, 2020). HT prevalence in developed countries is 0.1%–5% which, with modern dietary changes and greater longevity, is increasing annually. In recent years, the prevalence rate in the Chinese population has been reported at 11.5% (Taylor et al., 2018). HT onset is insidious and progresses slowly, without obvious clinical symptoms or with painless thyroid enlargement during its early stage. With disease progression, 50% of patients eventually develop permanent hypothyroidism (Taylor et al., 2018), the symptoms of which include sensitivity to cold, fatigue, constipation, weight gain, dry skin, arteriosclerosis, irregular menstruation, and bradycardia. Associations between hypothyroidism and cognitive function, attention deficit, memory impairment, and mood disorders, including depression and anxiety, are well-established (Bauer et al., 2008).

In autoimmune diseases, the involved thyroid autoantibodies are mainly thyroid-stimulating receptors and sodium-iodide symporter antibodies. Among these, thyroglobulin antibody (TgAb) and thyroid peroxidase antibody (TPOAb) are characteristically involved in HT, showing the cytotoxic effect of activating complement regulation of T lymphocytes. Simultaneously, HT induces sensitized killer T cells to destroy thyroid follicular function and participate in thyroid cell damaging processes (Zhu, 2018). TPOAb can destroy thyroid cells by activating complement regulation and antibody-dependent cell-mediated cytotoxicity. Several studies have shown that TgAb and TPOAb are positively correlated with increased thyroid inflammation and hypothyroidism. Patients with subclinical hypothyroidism with elevated anti-TPO and anti-Tg antibodies are more likely to progress to clinical hypothyroidism (Cappa et al., 2010; Garber et al., 2012).

Health-related quality of life (HRQoL) is the subjective assessment of daily physiological, emotional, and social functions and Wellbeing. In recent years, improving HRQoL has become an important goal in chronic disease treatment. As a chronic disease with a high incidence of thyroid dysfunction, HT affects patient psychological and physiological health and thus reduces their HRQoL. Thyroid autoimmunity, especially anti-TPO, is also associated with a decline in quality of life and increased depression (Bauer et al., 2008; Watt et al., 2012). High antibody level is one factor leading to development of HT-related symptoms. High titer anti-TPO and anti-Tg levels are significantly and negatively correlated with quality of life (Bektas Uysal and Ayhan, 2016), and high anti-TPO levels are positively correlated with depression risk (Delitala et al., 2016).

Currently, the pathogenesis of HT remains unclear and specific drug treatments are lacking. For patients with only increased thyroid autoantibodies, but no obvious symptoms and unclear thyroid enlargement, current treatments are generally unsuitable; with disease progression and the onset of clinical symptoms like decreased thyroid function and compression, surgery and thyroxine replacement therapy are available. Surgical removal of the thyroid gland can improve patient compression symptoms, yet postoperative hypothyroidism and impaired immune function further affect patient quality of life (Promberger et al., 2014). Thyroid hormone replacement therapy can improve thyroid function, but is ineffective at reducing thyroid autoantibody titers or interfering with the process of thyroid immune injury. Further, patients with adequate thyroid hormone supplementation still have various physical symptoms and unimproved quality of life (Bektas Uysal and Ayhan, 2016).

Traditional Chinese Medicine (TCM) classifies HT as yingbing, and its application has a long, rich history. According to the etiology and pathogenesis of HT, TCM treatment mainly adopts the methods of soothing the liver, regulating qi, resolving phlegm, and dispersing stasis. Bupleurum inula flower soup (BIFS) is a prescription based on the *Treatise on Febrile Diseases* and *Synopsis of the Golden Chamber*, which summarizes the experiences of past practitioners. In this approach, bupleurum, inula flowers, and pinellia are commonly prescribed to treat HT. A meta-analysis showed that soothing the liver and strengthening the spleen through TCM can effectively reduce serum thyroid stimulation, thyroglobulin resistance, and thyroid antioxidant enzyme resistance levels in patients with HT (Sa and Zhang, 2019). In the early stage of HT, a combination of BIFS and levothyroxine can reduce antibody titer and improve physical discomfort and mood symptoms, though high-quality prospective studies are lacking. Thus, the purpose of this study was to evaluate the clinical efficacy of BIFS in the treatment of HT immune injury and to examine quality of life effects using a strictly designed randomized controlled trial.

## Methods

### Participants

**FIGURE 1:**
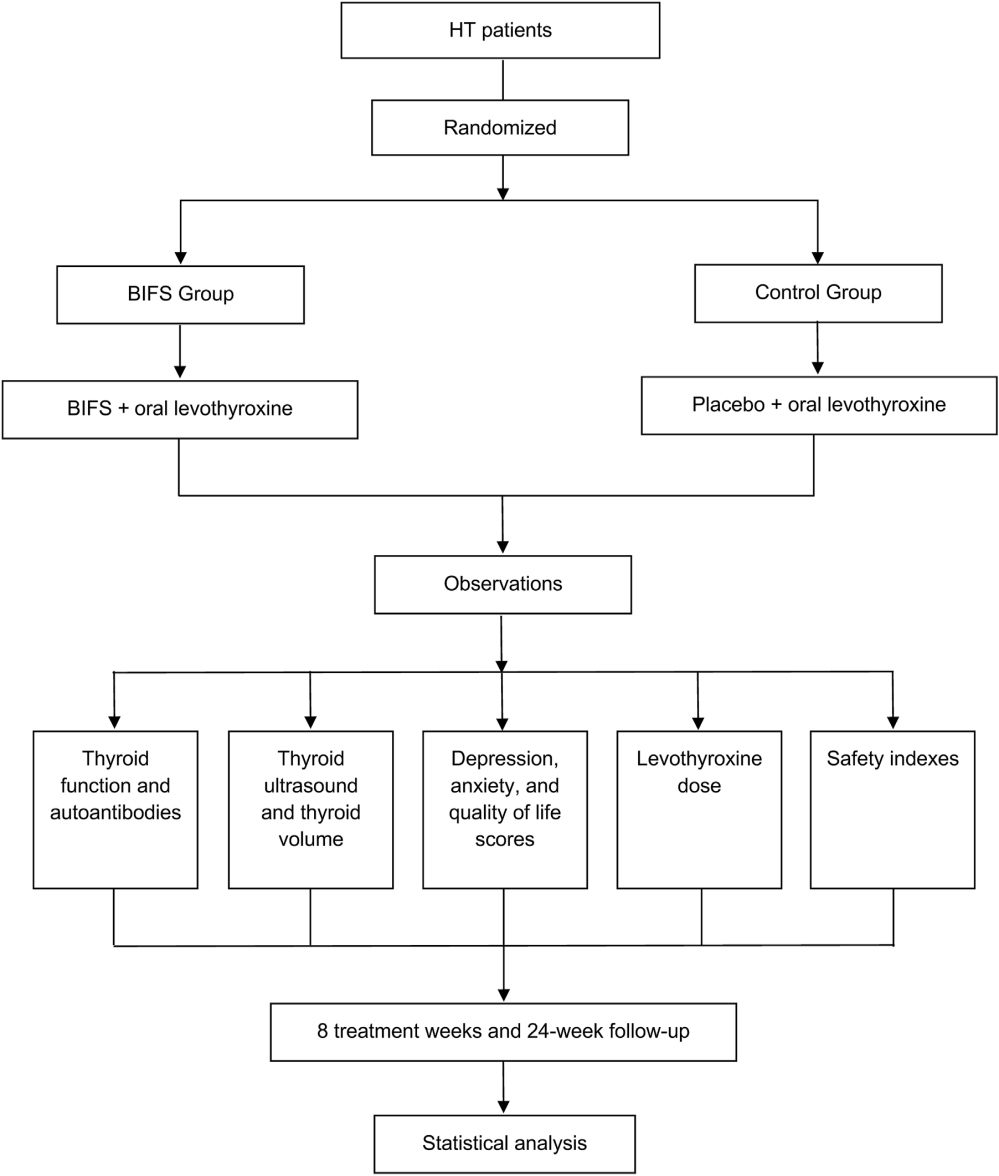
Clinical trial flowchart.

**FIGURE 2:**
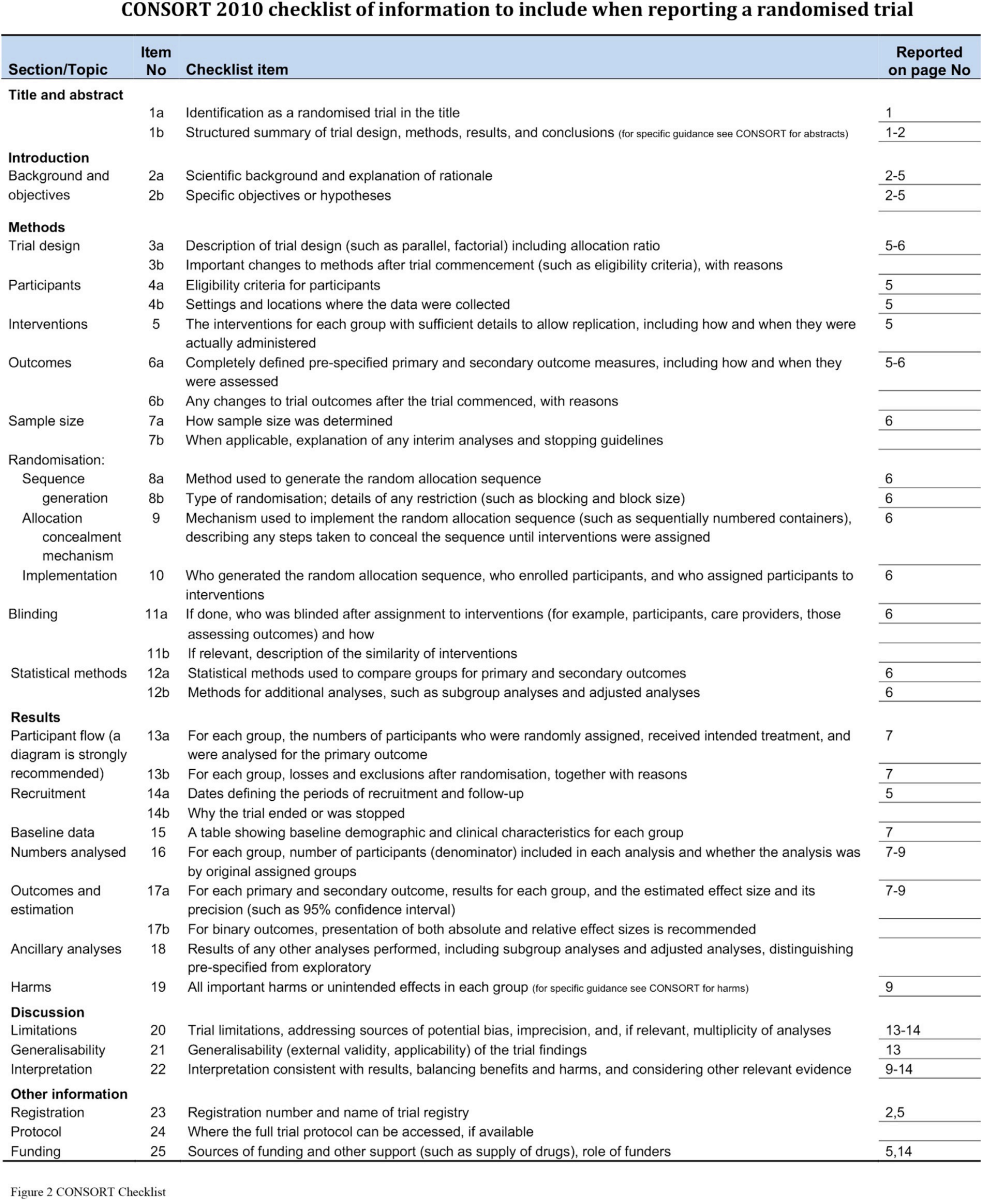
CONSORT checklist.

Study participants were patients who had been initially diagnosed with HT between December 2017 and April 2019 at Wangjing Hospital, which is affiliated with the China Academy of Chinese Medical Sciences, and the study was approved by its Clinical Research Ethics Committee (Chinese clinical trial registry: ChiCTR1900020987). Figure 1 shows the study clinical trial flowchart. Each participant provided written informed consent. This manuscript adheres to the applicable Consolidated Standards of Reporting Trials (CONSORT) guidelines (Figure 2).

Inclusion criteria were: aged 18–65 years; HT with hypothyroidism diagnosed by a doctor following the Chinese Guidelines for Diagnosis and Treatment of Thyroid Diseases (Endocrinology branch of Chinese Medical Association “Chinese thyroid disease diagnosis and treatment guidelines” Editorial Committee, 2007; Editorial Committee, 2008); and TCM differentiation syndrome belonging to liver stagnation and spleen deficiency, and formation of phlegm and blood stasis. The exclusion criteria were: history of thyroid malignant tumors; severe heart, liver, kidney or blood disease; severe mental disorder; pregnant or lactating woman; and allergy to TCM. Patients were randomly divided into two groups, described below.

### Intervention

Both groups were prescribed oral levothyroxine sodium tablets (German Merck Pharmaceuticals, H20140052, 50 ug/tablet) according to the Guidelines for Adult Hypothyroidism issued by the Endocrinology Branch of the Chinese Medical Association in March 2017. Dose was titrated and adjusted every 4 weeks based on the normal ranges of thyroid stimulating hormone (TSH), free triiodothyronine (FT3), and free tetraiodothyronine (FT4). The BIFS group was given BIFS, and the control group was given placebo.

The Chinese medicine test prescription was formulated as decoction-free granules at the Wangjing Hospital of the Chinese Academy of Chinese Medical Sciences as follows: Bupleurum (Apiaceae; *Bupleurum chinense* DC.) 10 g; Roasted White Peony (Paeoniaceae; *Paeonia lactiflora* Pall.) 15 g; Red Peony [Paeoniaceae; *Paeonia anomala* subsp. Veitchii (Lynch) D. Y. Hong and K. Y. Pan] 15 g; Pinellia [Araceae; *Pinellia ternata* (Thunb.) Makino] 10 g; Fritillaria (Liliaceae; *Fritillaria thunbergii* Miq.) 15 g; Prunella (Lamiaceae; *Prunella vulgaris* L.) 15 g; Cyperus rotundus (Cyperaceae; *Cyperus rotundus* L.) 10 g; Codonopsis [Campanulaceae; *Codonopsis pilosula* (Franch.) Nannf.] 12 g; Inula (Asteraceae; *Inula helenium* L.) 30 g; Semen ziziphi spinosae [Rhamnaceae; etc].

The placebo was produced by Hebei Yinfeng Food Technology Co., Ltd. Both BIFS and placebo groups underwent standard baseline pre-treatment evaluations, including: collection of demographics and medical history; physical examination; tests for thyroid autoantibodies and thyroid function; assessment of safety indicators [electrocardiogram and routine blood tests, including white blood cell (WBC), red blood cell (RBC), platelet (PLT), and liver and kidney functions including alanine aminotransferase (ALT), aspartate aminotransferase (AST), blood urea nitrogen (BUN), and blood creatinine (CRE)]; thyroid ultrasound, inventory scores for depression, anxiety, and HRQoL, and levothyroxine dose. After eight treatment weeks and at the 24-week follow-up, we evaluated clinical efficacy and safety in both groups, and all relevant patient treatment information was recorded using a case report form.

### Liquid chromatography analysis of BIFS group and placebo group

Agilent 1260II-DAD-ELSD (Agilent Technologies, United States) was used for liquid chromatography analysis. The column was YMC-Triart C18 ExRS 5 μM 250 mm × 4.6 mm and the column oven was set to 30°C. The stationary phase was eluted with mobile phase A (water with 1‰ trifluoroacetic acid) and mobile phase B (acetonitrile). The mobile phases were delivered in gradient mode as described in Table 1 for a total run of 30 min at a flow rate of 1 ml/min. The DAD and ELSD diagrams of each group are shown in Supplementary Figures S1–S4; Supplementary Materials S1, S2.

**TABLE 1:** Optimized gradient for liquid chromatography analysis.

Time (min)	A (%)	B (%)	Flow rate (ml/min)
0	95	5	1.00
20	50	50	1.00
25	20	80	1.00
30	0	100	1.00

### Thin-layer BIFS chromatograms

The thin-layer chromatograms of BIFS are in Supplementary Figures S5–S12.

### Laboratory tests, thyroid volume, and quality of life, depression, and anxiety scores

TPOAb and TgAb were measured by radioimmunoassay kit (Weifang 3d Bioengineering Group Co., Ltd.). FT3, FT4, and TSH were measured by chemiluminescence immunoassay. AST, ALT, BUN, and CRE were measured by enzyme coupling. WBC, RBC, and PLT were measured by combined detection of electrical impedance radio frequency and cytochemistry.

Thyroid ultrasound was performed by the same blind investigator before and after treatment and at follow-up. Long axis diameter (D1), short axis diameter (D2), and thickness (D3) of both thyroid lobes were measured. Lateral lobe thyroid volume was 0.479 × D1 × D2 × D3 and the total thyroid volume was calculated as the sum of the volumes of both lobes (Brunn et al., 1981).

Quality of life score was based on the World Health Organization’s quality of life summary (WHOQOL-BREF-36), indicating HRQoL with four subscales: physiological, psychological, social, and environmental. Zung’s self-rating depression and anxiety scales were administered before and after treatment and at follow-up; higher scores on each indicate more serious degrees of depression and anxiety, respectively.

### Sample size calculation

Sample size was calculated based on comparisons of two independent samples (rate/count data) sample content calculation using the formula:

$$N={\left\{Z_{\alpha /2}{\left[\left(2P_{average}\right)\left(1-P_{average}\right)\left(Q_{1}^{-1}+Q_{2}^{-1\;}\right)\right]}^{0.5}+Z_{\beta }{\left[P_{1}Q_{1}^{-1}\left(1-P_{1}\right)+P_{2}Q_{2}^{-1}\left(1-P_{2}\right)\right]}^{0.5}\right\}}^{2}/{\left(P_{1}-P_{2}\right)}^{2}$$

Pilot studies determined that clinical efficacy of TCM was 85%, and that of placebo was 50%. The between-groups sample size ratio was 1:1. Other variables were: Zα/2: α = 0.05; Zα/2 = 1.960; Zβ: β = 0.20; Q1, Q2: were the sample ratios of each group where the example design Q1 = Q2 = 0.50; P1, P2 were the pretest rates of each group. This shows an effective rate where: P1(BIFS group) = 0.85, P2(control group) = 0.45. Thus, calculations indicated that BIFS group = control group = 22 cases. Assuming a loss rate of 10%, the final sample for recruitment was: BIFS group = control group = 22 cases + 3 cases = 25 cases.

### Clinical efficacy

The criteria for evaluating clinical efficacy was based on the Guiding Principles for Clinical Research of New Chinese Medicines (Zheng, 2002) and referred to clinical diagnosis and treatment. A decrease in total scores for clinical symptoms and signs by >70% and autoantibody improvement by >30% were considered significantly effective. A decrease in total scores for clinical symptoms and signs between 30% and 69% and autoantibody improvement 10%–29% were considered effective. A decrease in total scores for clinical symptoms and signs <29% and autoantibody improvement <9% were considered ineffective. Effective rate was calculated using the formula:

$$Effective\;rate=\frac{{Quantity}_{\left(significantly\;effective\right)}+{Quantity}_{\left(effective\right)}\;}{{Quantity}_{\left(total\right)}}\times 100\%$$

### Randomization

Independent statisticians used the Statistical Package for Social Sciences (SPSS, International Business Machines Corporation, United States) version 20.0 to generate random numbers, which were then kept in opaque envelopes. Randomized code generation and drug blindness were implemented independent of data collection, evaluation, and analysis. Drug administrators enrolled patients during screening, and neither participants nor researchers were informed of the assignment until the end of the trial. The two groups’ medications were packaged in the same way, and the placebo had the same color, shape, taste, and smell as the test Chinese medicine.

### Statistical analysis

Statistical analyses were carried out using SPSS version 20.0. Normally distributed descriptive statistics are expressed as mean ± standard deviation (SD) and otherwise as median and quartile. Categorical data are described as frequencies and percentages and were analyzed with the χ^2^ test. The Kolmogorov–Smirnov test was used to evaluate normality. If normally distributed, continuous variables were compared between groups using independent samples *t*-tests, and within-group pre-post-test comparisons were made using paired samples *t*-tests. The Wilcoxon rank sum test was used when distribution normality was not met.

## Results

### Participants and baseline characteristics

**FIGURE 3:**
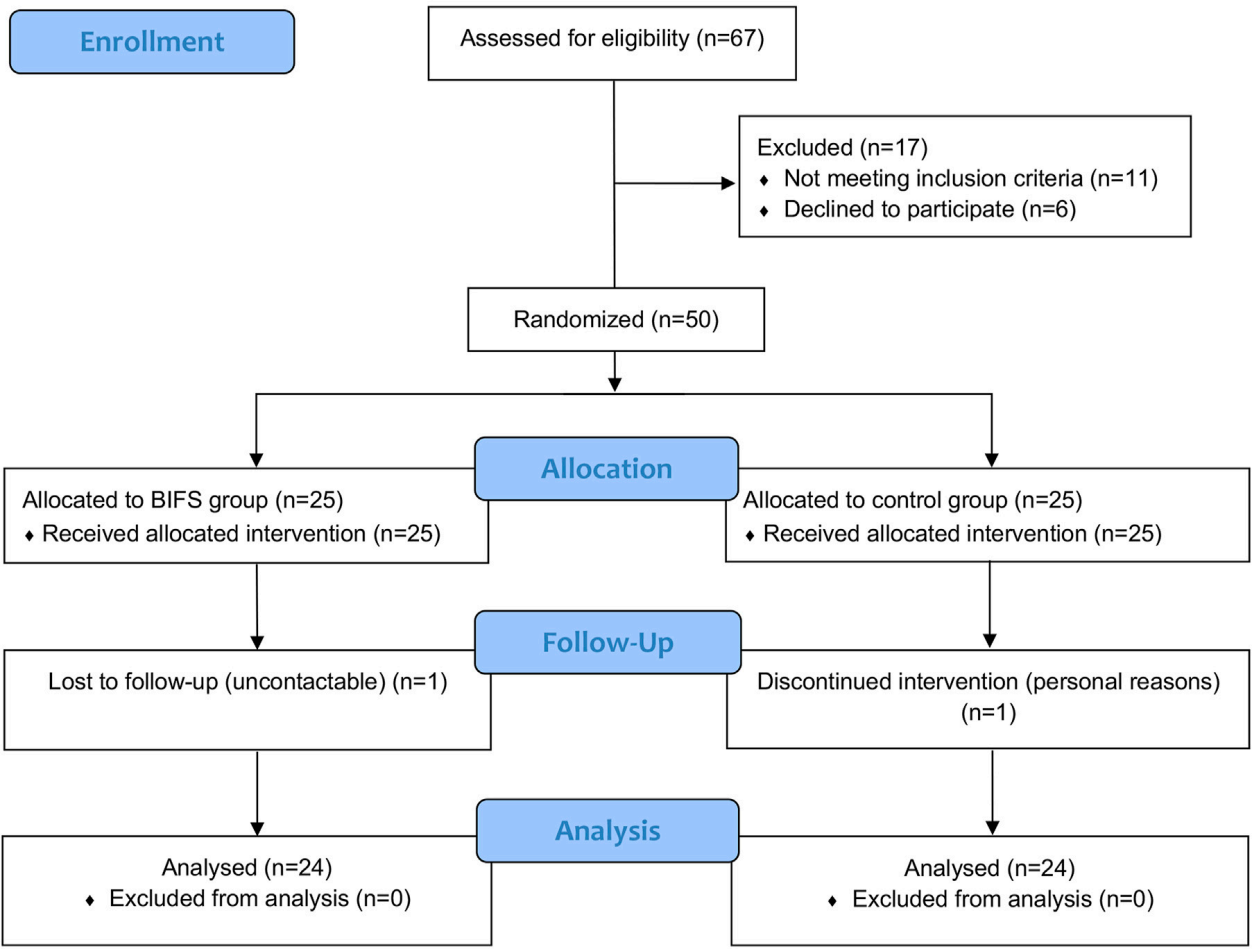
CONSORT flow diagram.

A total of 67 patients with HT were screened for eligibility from December 2017 to April 2019, among whom 17 were excluded and 50 were enrolled and randomly assigned to either the BIFS (25 patients) or control (25 patients) group. Figure 3 shows descriptive information for the 48 patients who completed the study and were included in the final analysis.

Among the final sample of 48 participants, women accounted for 87.5%. The control and BIFS groups were an average of 45.79 ± 13.38 years and 48.17 ± 13.22 years, respectively, and did not differ significantly (Table 2). TSH levels in both groups were higher than normal due to the immune damage to thyroid cells from HT; however, these levels did not differ significantly between the groups (*p* > 0.05), indicating that they were comparable before treatment (Supplementary Table S1).

**TABLE 2:** Baseline characteristics of two patient groups.

Variable	BIFS group	Control group
Number of patients [n]	24	24
Age [years; mean (SD)]	48.17 (13.22)	45.79 (13.38)
Female [n (%)]	20 (83.3%)	22 (91.7%)
BMI [mean (SD)]	21.32 (7.8)	22.75 (4.9)
Disease course [years; mean (SD)]	1.92 (1.62)	2.09 (1.96)
Diabetes [n (%)]	3 (12.5%)	2 (8.3%)
SLE [n (%)]	0 (0%)	0 (0%)
Hypertension [n (%)]	4 (16.7%)	6 (25%)

SD, standard deviation; BMI, body mass index; SLE, systemic lupus erythematosus.

### Thyroid autoantibodies

**FIGURE 4:**
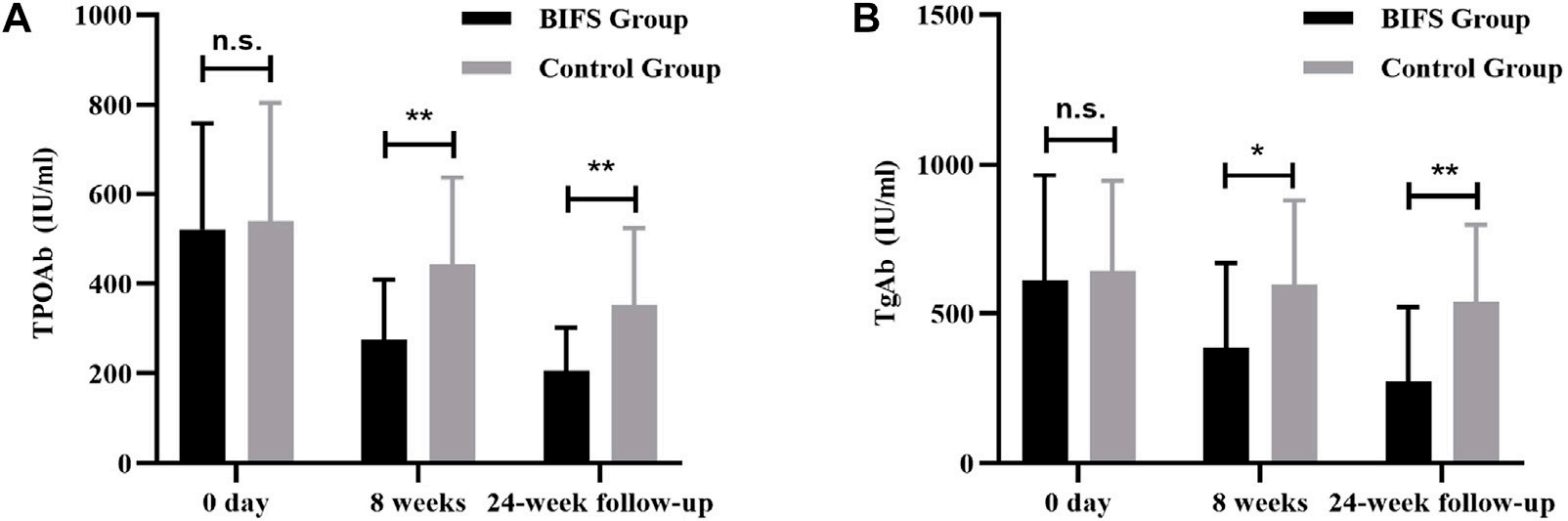
TPOAb and TGAb titers at baseline, after eight treatment weeks, and at 24-week follow-up. **(A)** TPOAb titer; **(B)** TGAb titer; n.s., no statistically significant difference; *p < 0.05; **p < 0.01.

Before treatment, there was not a significant between-group difference (*p* > 0.05) in TPOAb or TgAb. After eight treatment weeks, TPOAb levels in both groups decreased, with a more significant decrease in the BIFS group (275.77 ± 132.98 vs. 441.78 ± 195.50, *p* < 0.01). During the same period, TgAb levels in the BIFS group decreased significantly after treatment compared with the control group (385.92 ± 281.91 vs. 596.17 ± 282.26, *p* < 0.05). At the 24-week follow-up, BIFS group TPOAb and TgAb levels were still significantly lower than those in the control group (205.75 ± 96.20 vs. 352.67 ± 171.18, *p* < 0.01 and 274.38 ± 247.28 vs. 538.92 ± 258.12, *p* < 0.01, respectively) (Figure 4).

### Thyroid function

**FIGURE 5:**
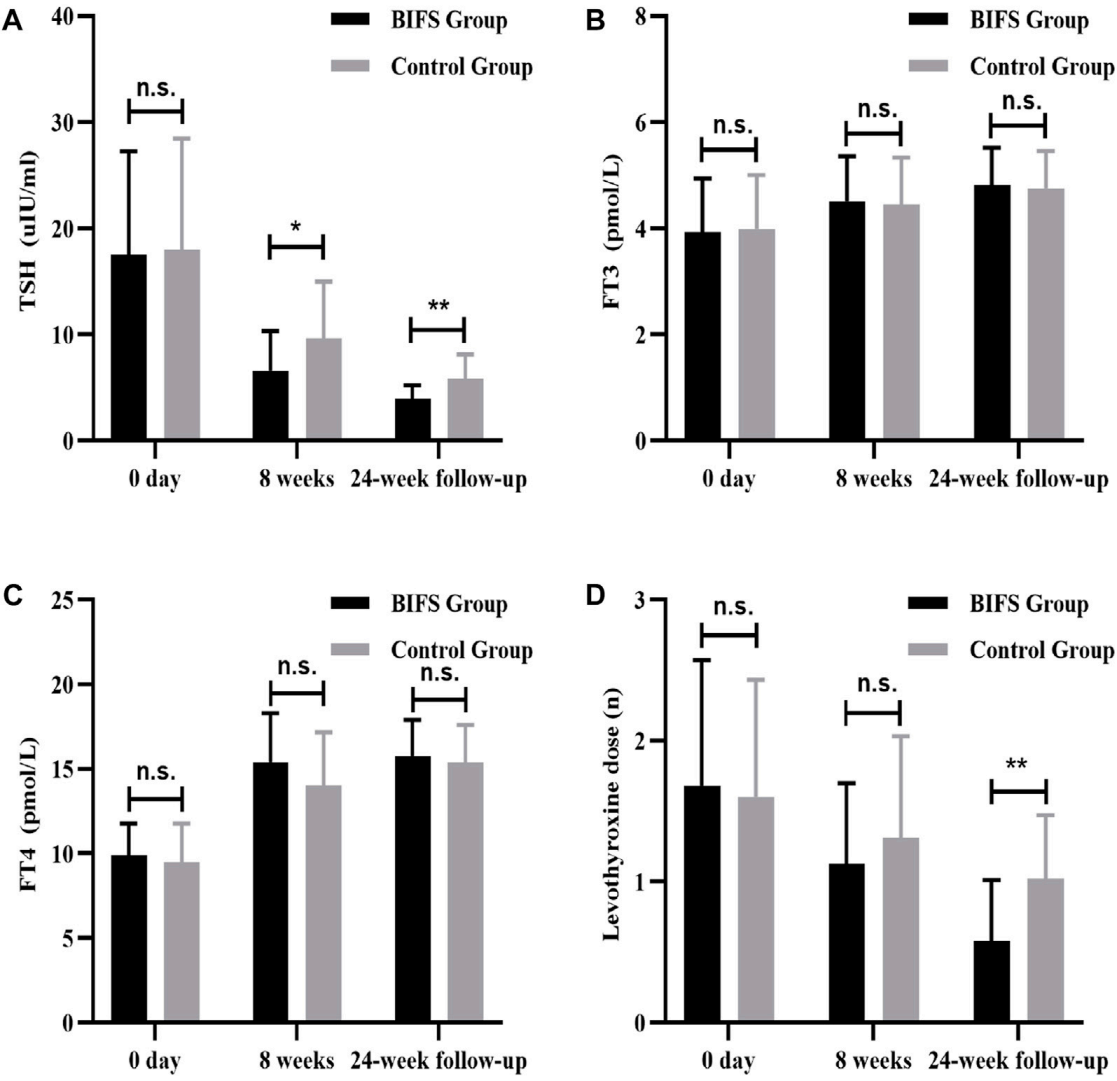
TSH, FT3, FT4, and levothyroxine levels at baseline, after eight treatment weeks, and at 24-week follow-up. **(A)** TSH level; **(B)** FT3 level; **(C)** FT4 level; **(D)** levothyroxine dose; n.s., no statistically significant difference; *p < 0.05; **p < 0.01.

Before treatment, there was not a significant between-group difference (*p* > 0.05) in TSH, FT3, FT4, or levothyroxine dose. After eight treatment weeks, TSH level in the BIFS group decreased significantly (6.57 ± 3.73 vs. 9.63 ± 5.34, *p* < 0.05), though both groups reached a normal post-treatment level. Follow-up at 24 weeks showed that TSH in the BIFS group had decreased to a normal level, and that the two groups differed significantly (3.94 ± 1.26 vs. 5.82 ± 2.29, *p* < 0.01) (Figure 5A). After eight treatment weeks and at the 24-week follow-up, FT3 and FT4 levels increased, but no significant differences were observed (Figures 5B,C).

### Thyroid volume changes

**FIGURE 6:**
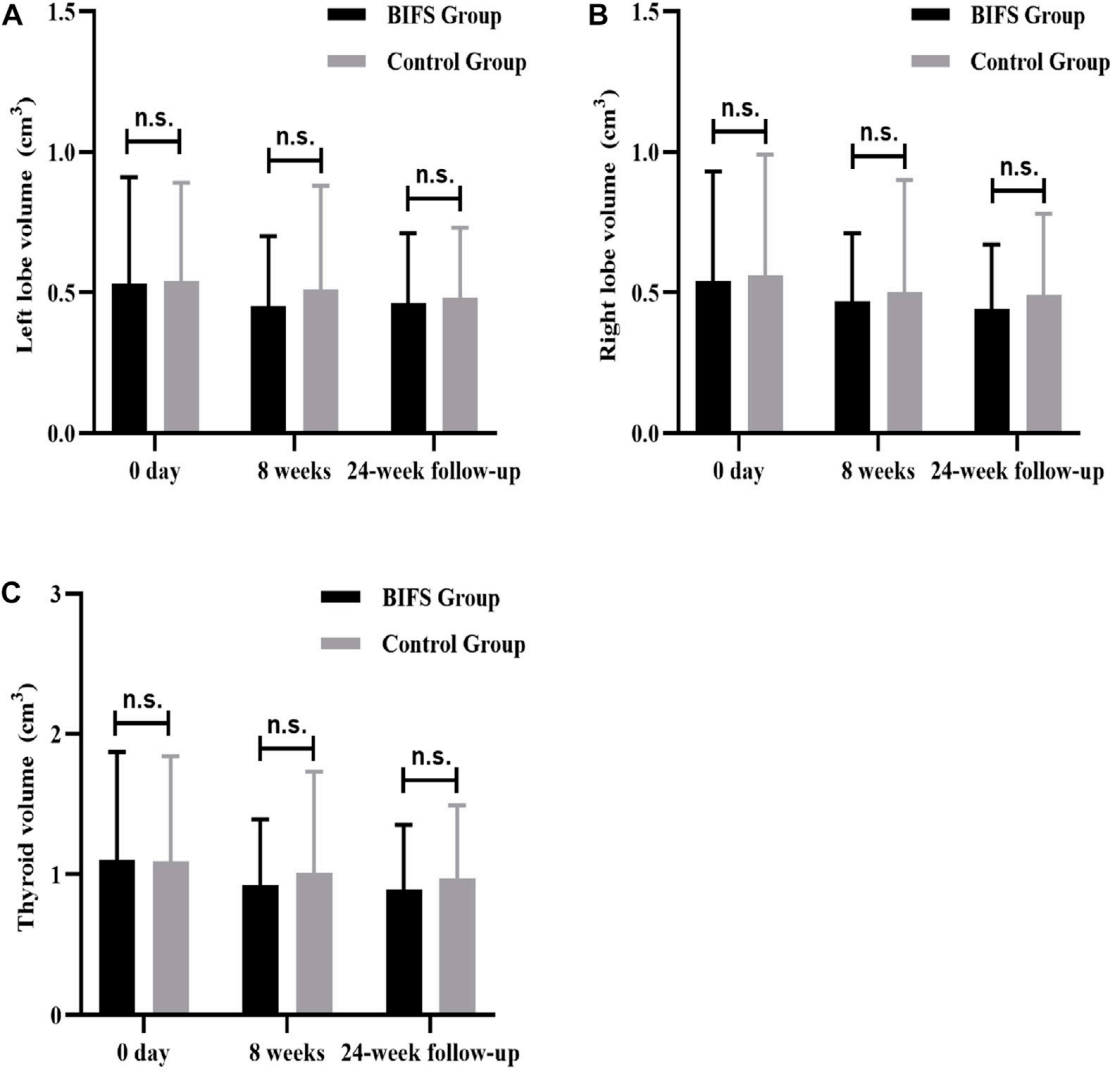
Thyroid volume differences at baseline, after eight treatment weeks, and at 24-week follow-up. **(A)** Thyroid volume of left lobe; **(B)** Thyroid volume of right lobe; **(C)** Total thyroid volume; n.s., no statistically significant difference.

There was not a significant between-group difference in thyroid volume at baseline (*p* > 0.05), After eight treatment weeks and at the 24-week follow-up, left lobe, right lobe, and total thyroid volumes decreased, but there was not a significant difference from pre- to post-treatment (*p* > 0.05). Nor was there a significant difference between the groups (*p* > 0.05) (Figures 6A–C). In other words, both treatments had less effect on thyroid volume.

### Depression, anxiety, and quality of life scores

**FIGURE 7:**
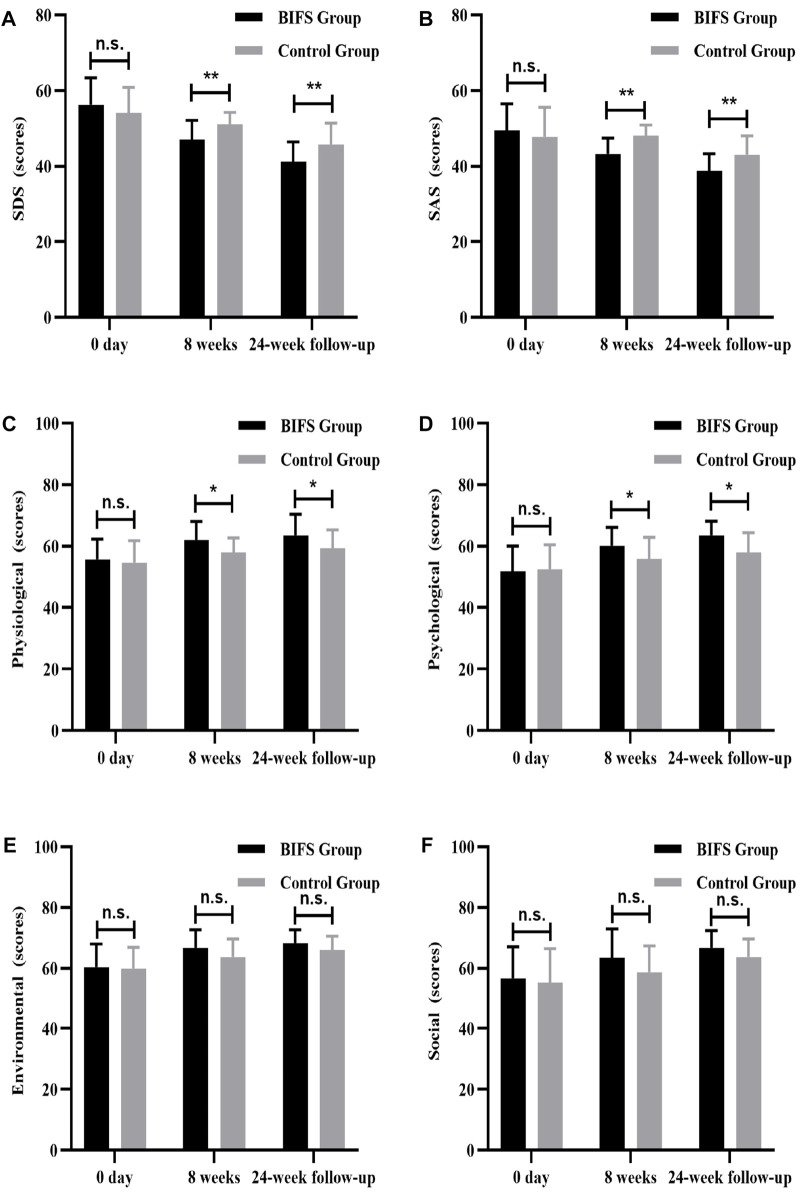
Depression, anxiety, and quality of life scores at baseline, after eight treatment weeks, and at 24-week follow-up. **(A)** Depression score, **(B)** anxiety score, **(C)** physiological function score, **(D)** psychological function score, **(E)** social function score, **(F)** environmental function score. n.s., no statistically significant difference, *p < 0.05, **p < 0.01.

There were no significant between-group differences in depression, anxiety, or HRQoL scores at baseline (*p* > 0.05). After eight treatment weeks and at the 24-week follow-up, BIFS group depression scores were significantly lower than those of the control group (47.0 ± 5.12 vs. 51.04 ± 3.22, *p* < 0.01; 41.17 ± 5.29 vs. 45.79 ± 5.60, *p* < 0.01, respectively). BIFS group anxiety scores were also significantly lower than that of the control group at both timepoints (43.21 ± 4.22 vs. 48.08 ± 2.81, *p* < 0.01; 38.79 ± 4.51 vs. 43.08 ± 4.93, *p* < 0.01, respectively). Anxiety and depression scores of BIFS participants got better with treatment (Figures 7A,B). For HRQoL, after eight treatment weeks, BIFS group physiological and psychological subscores were significantly higher compared with the control group (62.08 ± 5.97 vs. 57.96 ± 4.71, *p* < 0.05; 60.17 ± 5.94 vs. 55.75 ± 7.09, *p* < 0.05, respectively). At the 24-week follow-up, there were still significant between-group differences (63.58 ± 6.76 vs. 59.33 ± 5.95, *p* < 0.05; 63.54 ± 4.62 vs. 57.96 ± 6.40, *p* < 0.05, respectively) (Figures 7C,D). After eight treatment weeks, BIFS group social and environmental scores increased compared with the control group, but there were no statistically significant differences (63.46 ± 9.48 vs. 58.67 ± 8.74, *p* > 0.05; 66.71 ± 5.69 vs. 63.67 ± 5.99, *p* > 0.05, respectively). At the 24-week follow-up, there was no significant between-group difference (*p* > 0.05) (Figures 7E,F).

### Clinically effective rate

After treatment, the BIFS group’s clinically effective rate (75%; 18/24) was significantly higher (*p* < 0.05) than that of the control group (46%; 11/24) (Table 3).

**TABLE 3:** Clinically effective rate of two groups.

Group	Significantly effective (n)	Effective (n)	Ineffective (n)	Total effective rate
Experimental group	7	11	6	75%
Control group	3	8	13	46%
χ^2^ value	—	—	—	4.26
P	—	—	—	0.03

### Safety indicators

**FIGURE 8:**
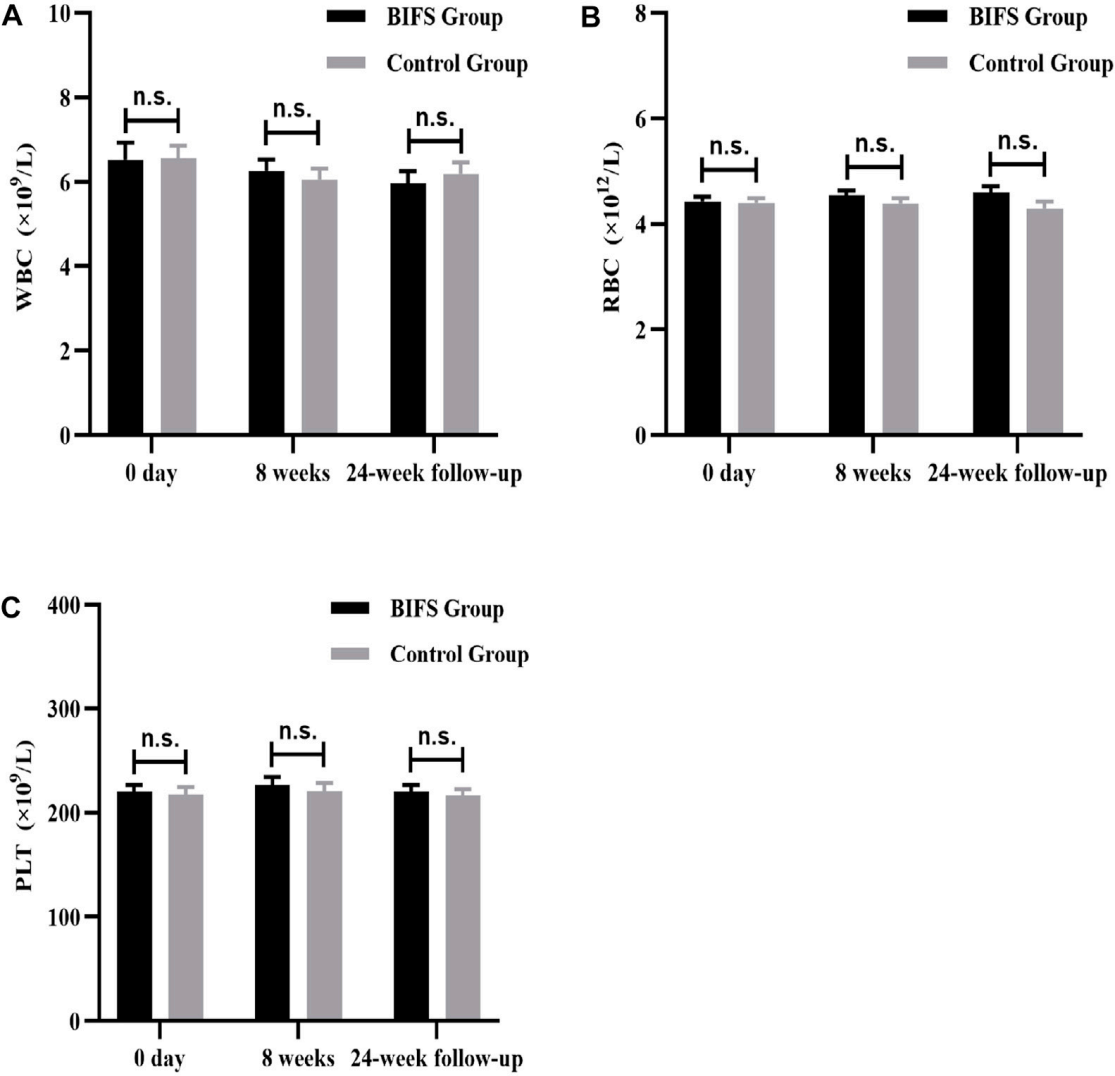
Routine blood tests, after eight treatment weeks, and at 24-week follow-up. **(A)** WBC, **(B)** RBC, **(C)** PLT. n.s., no statistically significant difference, *p < 0.05, **p < 0.01.

**FIGURE 9:**
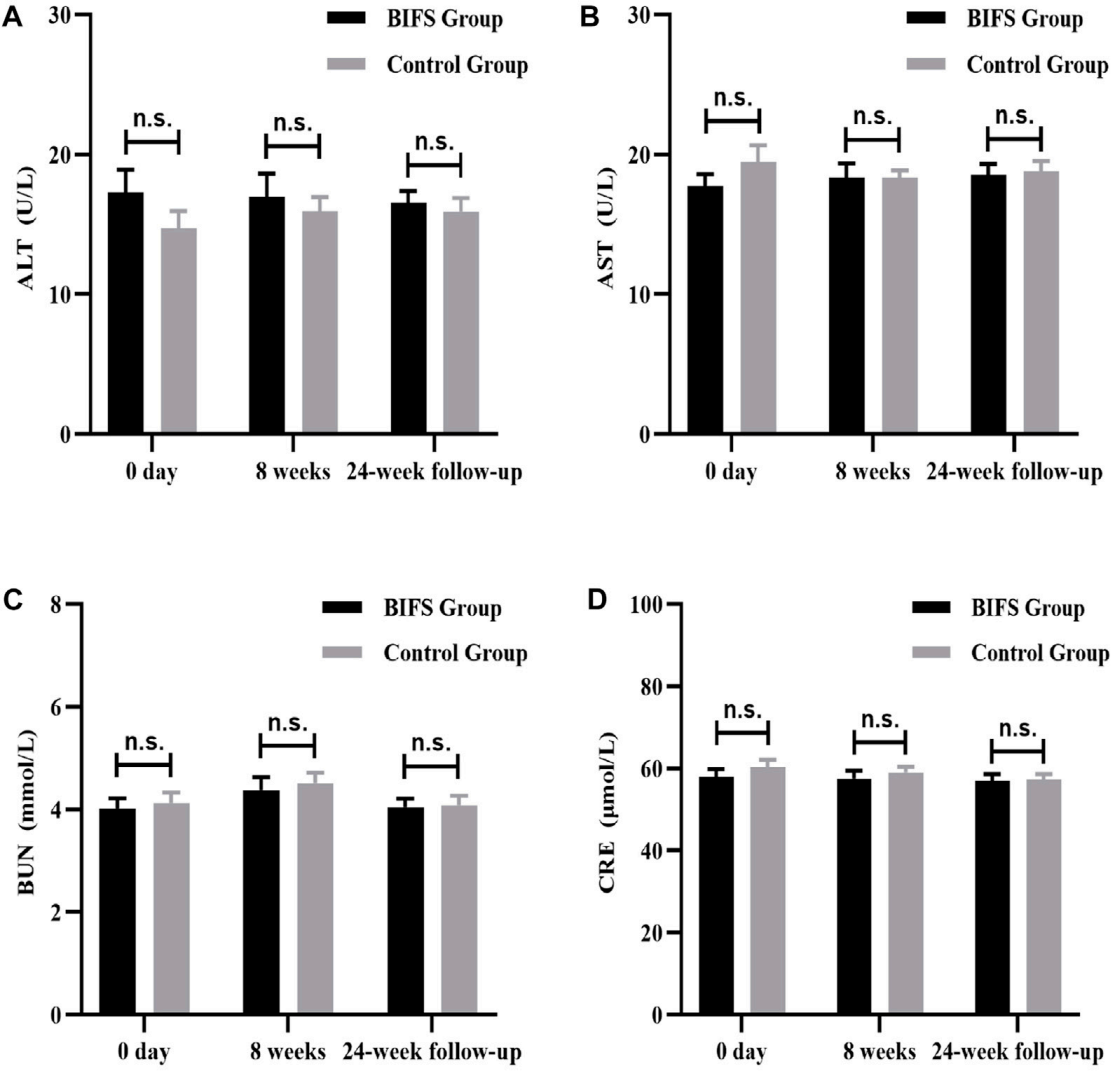
Liver and kidney functions at baseline, after eight treatment weeks, and at 24-week follow-up. **(A)** ALT, **(B)** AST, **(C)** BUN, **(D)** CRE. n.s., no statistically significant difference, *p < 0.05, **p < 0.01.

Both before and after treatment, there were no obvious abnormalities on safety indicators, including routine blood tests (WBC, RBC, and PLT) (Figure 8), liver and kidney function (ALT, AST, BUN, and CRE) (Figure 9), and electrocardiogram. No adverse reactions occurred in either group.

### Levothyroxine dose

At neither the beginning nor the end of the eight treatment weeks was there a significant between-group difference in levothyroxine dose (*p* > 0.05). At the 24-week follow-up, the levothyroxine dose in the BIFS group was lower than in the control group (0.58 ± 0.43 vs. 1.02 ± 0.45, *p* < 0.05). (Figure 5D).

## Discussion

Thyroid autoantibodies are mainly produced by lymphocytes infiltrating the thyroid gland and, to a lesser extent, by local lymph nodes or bone marrow (Antonelli et al., 2015). TPOAb, a membrane-bound heme protein, is an important enzyme in thyroid hormone synthesis and plays a key role in the iodization and conjugation of thyroglobulin tyrosine residues (Cheng et al., 2020). TgAb is an antibody formed after thyroglobulin in thyroid follicles enters the blood, which has a destructive effect on thyroid follicular epithelium (Saravanan and Dayan, 2001). Anti-TPO and anti-Tg antibodies are a prominent feature of HT and exist in almost all patients with this condition (Garber et al., 2012). The titer of anti-TPO antibody is related to the severity of thyroid lymphocyte infiltration (Czarnocka et al., 1997; Fink and Hintze, 2010; Czarnocka, 2011) and anti-TPO antibody increases prior to TSH level (Vanderpump et al., 1995). In addition to assisting diagnosis, anti-TPO and anti-Tg antibody levels can predict the development of hypothyroidism (González et al., 2007). Epidemiological investigations have shown that in long-term follow-up, patients with positive anti-TPO and anti-Tg antibodies are more likely to develop hypothyroidism than are those who are negative (Cappa et al., 2010). Herein, we evaluated thyroid autoantibody titers after treatment in both groups, showing that levothyroxine combined with TCM reduced antibody titers and TSH levels better than levothyroxine monotherapy.

With the development of HT, hypothyroidism can be caused by thyroid hormone deficiency. This deficiency can cause serious dysfunction in physiological, psychological, and social factors (Bektas Uysal and Ayhan, 2016). Mental health is among the most important indicators of social functioning and overall health, and a lack of thyroid hormones can affect a patient’s sensory and emotional functioning (Bektas Uysal and Ayhan, 2016). Increased anxiety and depression scores can be seen in hypothyroidism researches (Watt et al., 2012). Some studies have also shown that autoimmunity is independent of thyroid function and affects the HRQoL of patients with HT, especially in terms of psychological symptoms (Watt et al., 2012). In addition to thyroid hormone deficiency, the mental health of patients with HT is also related to thyroid autoantibodies. Patients with positive TPOAb are more prone to mood disorders (Watt et al., 2012).

At present, the standard treatment for HT is to replace thyroid hormone with levothyroxine. Although this method can exogenously supplement necessary thyroid hormone, Escobar-Morreale et al. (2005) assert that this therapy has little impact on patients’ physical discomfort, mood, or quality of life. The findings herein suggest that compared with levothyroxine alone, levothyroxine plus BIFS better reduces the thyroid autoantibody titer, interfering with the process of HT immune injury, improving HT prognosis, and even reversing the trend toward hypothyroidism with long-term use of BIFS. Levothyroxine combined with BIFS may also improve these patients’ depression and anxiety symptoms and quality of life. This study provides objective evidence about the impacts of TCM intervention in HT.

TCM theory holds that HT and negative emotions like depression and anxiety symptoms are related. The TCM prescription used herein is from the most well-respected medical book *Treatise on Febrile Diseases and Miscellaneous Diseases* in the Eastern Han Dynasty in China, in which it is recorded that the prescription composed mainly of Bupleurum, Inula, and Pinellia ternata can improve emotional disorders. The results herein also confirm that BIFS combined with levothyroxine can effectively improve depression, anxiety, and HRQoL scores (specifically physiological and psychological subscores) compared with placebo.

Patients with HT with hypothyroidism must take levothyroxine for life, which may also lead to mood disorders. Reducing the dose may thus alleviate patient emotional burden. Herein, we found that adding TCM may allow reduction of the levothyroxine dose. Two of our participants were able to stop taking levothyroxine and retain normal thyroid function without obvious discomfort.

There were also some study limitations. The sample was only 50 participants, two of whom withdrew. Overall, relatively few outcomes were assessed, and change in antibody titer was not followed long-term. The lack of pathological indicators disallows clarifying the mechanism by which TCM is an effective intervention. In future studies, in addition to larger samples, it will be important to include pathological and advanced biomarkers that reflect disease mechanism, including TH17 and Treg, to objectively evaluate the clinical efficacy of TCM interventions in HT.

## Data Availability

The original contributions presented in the study are included in the article/Supplementary Material, further inquiries can be directed to the corresponding authors.

## Abbreviations

ALT, alanine aminotransferase; AST, aspartate aminotransferase; BIFS, bupleurum inula flower soup; BMI, body mass index; BUN, blood urea nitrogen; CONSORT, consolidated standards of reporting trials; CRE, blood creatinine; FT3, free triiodothyronine; FT4, free tetraiodothyronine; HRQoL, health-related quality of life; HT, Hashimoto’s thyroiditis; PLT, platelet; RBC, red blood cell; SD, standard deviation; SLE, systemic lupus erythematosus; SPSS, statistical package for social sciences; TCM, traditional Chinese medicine; TPOAb, thyroid peroxidase antibody; TgAb, thyroglobulin antibody; TSH, thyroid stimulating hormone; WBC, white blood cell.
