# Supplementary material for: Clinical efficacy of Bupleurum inula flower soup for immune damage intervention in Hashimoto’s thyroiditis: A placebo-controlled randomized trial
Source: Front Pharmacol. 2022 Nov 24;13:1049618. doi: 10.3389/fphar.2022.1049618 (PMC9730284; doi:10.3389/fphar.2022.1049618)
Supplement: Supplementary file 1 [file DataSheet7.pdf]

R White

Clean, RemissionVis

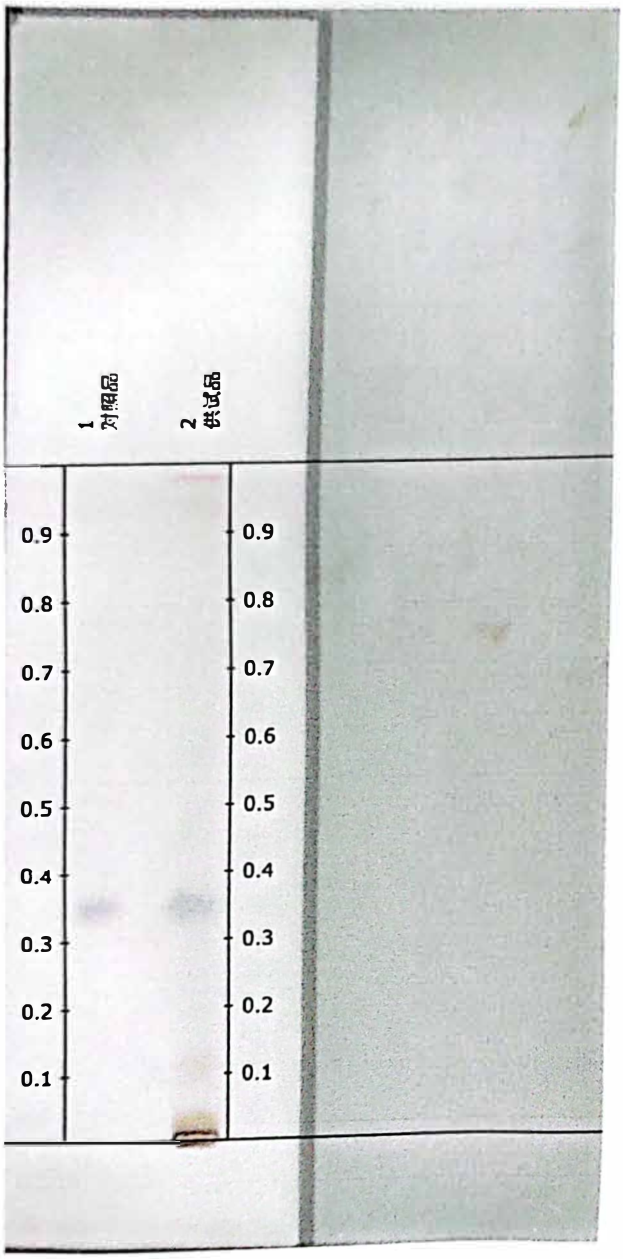

Exposure	0.151 s
Contrast	1
Normalized exposure	Disabled
Clarify	Disabled
White balance	1.00, 1.00, 1.00

Log:

26-Feb-2022 11:38:12 - 许静秋 - hpz240: File created with name '/Demo Project/2022年2月/药材/赤芍（药药）（药材）010070-2202001'

Steps

Plate layout

赤芍（芍药）010070-2202001

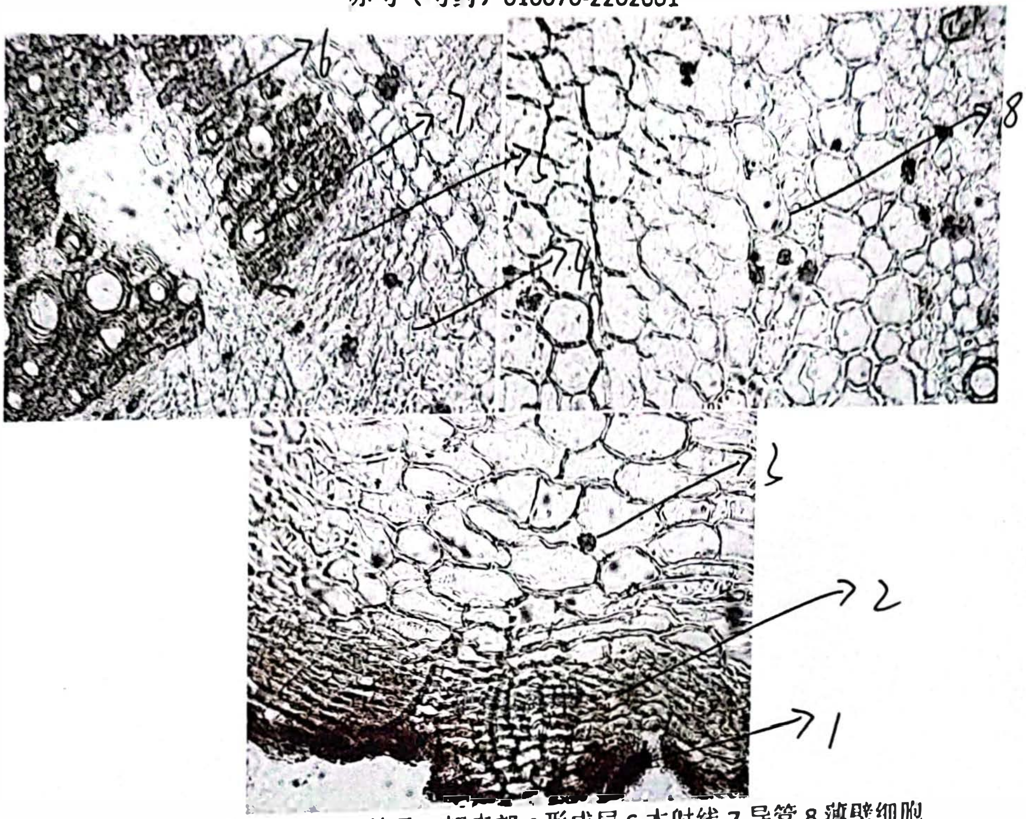

1.木栓层 2.栓内层 3.草酸钙簇晶 4.韧皮部 5.形成层 6.木射线 7.导管 8.薄壁细胞

Figure S11 Red Peony
