# Supplementary material for: Clinical efficacy of Bupleurum inula flower soup for immune damage intervention in Hashimoto’s thyroiditis: A placebo-controlled randomized trial
Source: Front Pharmacol. 2022 Nov 24;13:1049618. doi: 10.3389/fphar.2022.1049618 (PMC9730284; doi:10.3389/fphar.2022.1049618)
Supplement: Supplementary file 2 [file DataSheet2.pdf]

CAMAG®
World Leader in Planar Chromatography

R 254

Clean, Remission254

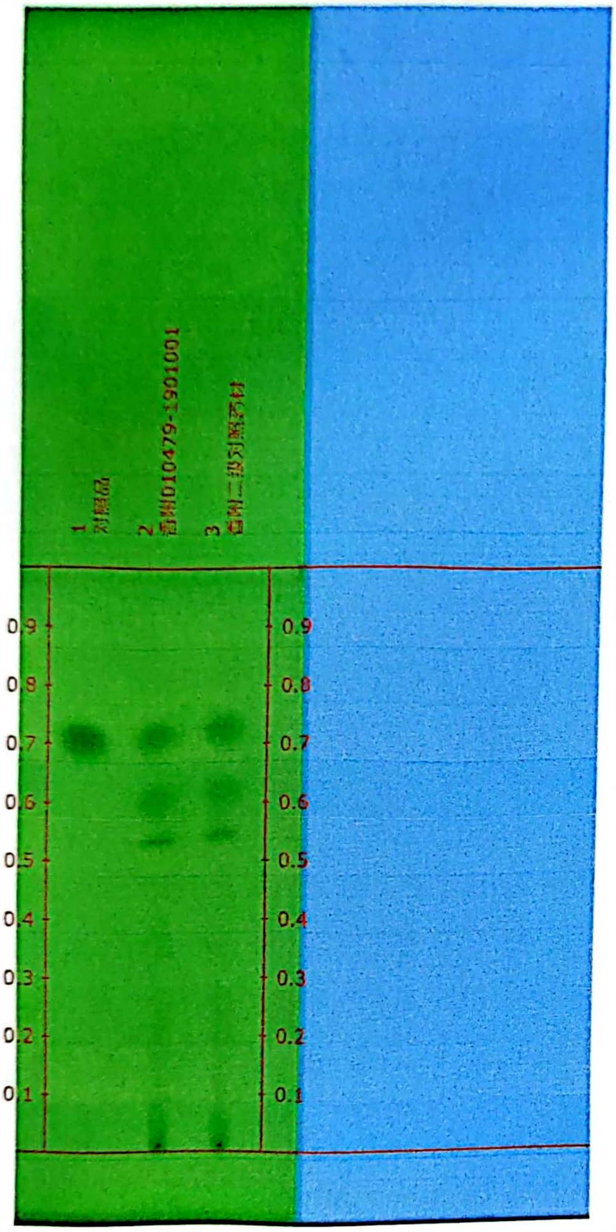


| | |
|---|---|
| Exposure | 0.320 s |
| Contrast | 1 |
| Normalized exposure | Disabled |
| Clarify | Disabled |
| White balance | 1.00, 1.00, 1.00 |

## Take image clean plate 1b - Visualizer (S/N: 240188):

| | |
|---|---|
| Executed | 28-Sep-2021 14:04:37 李潜梅 |

香附 010479-1901001

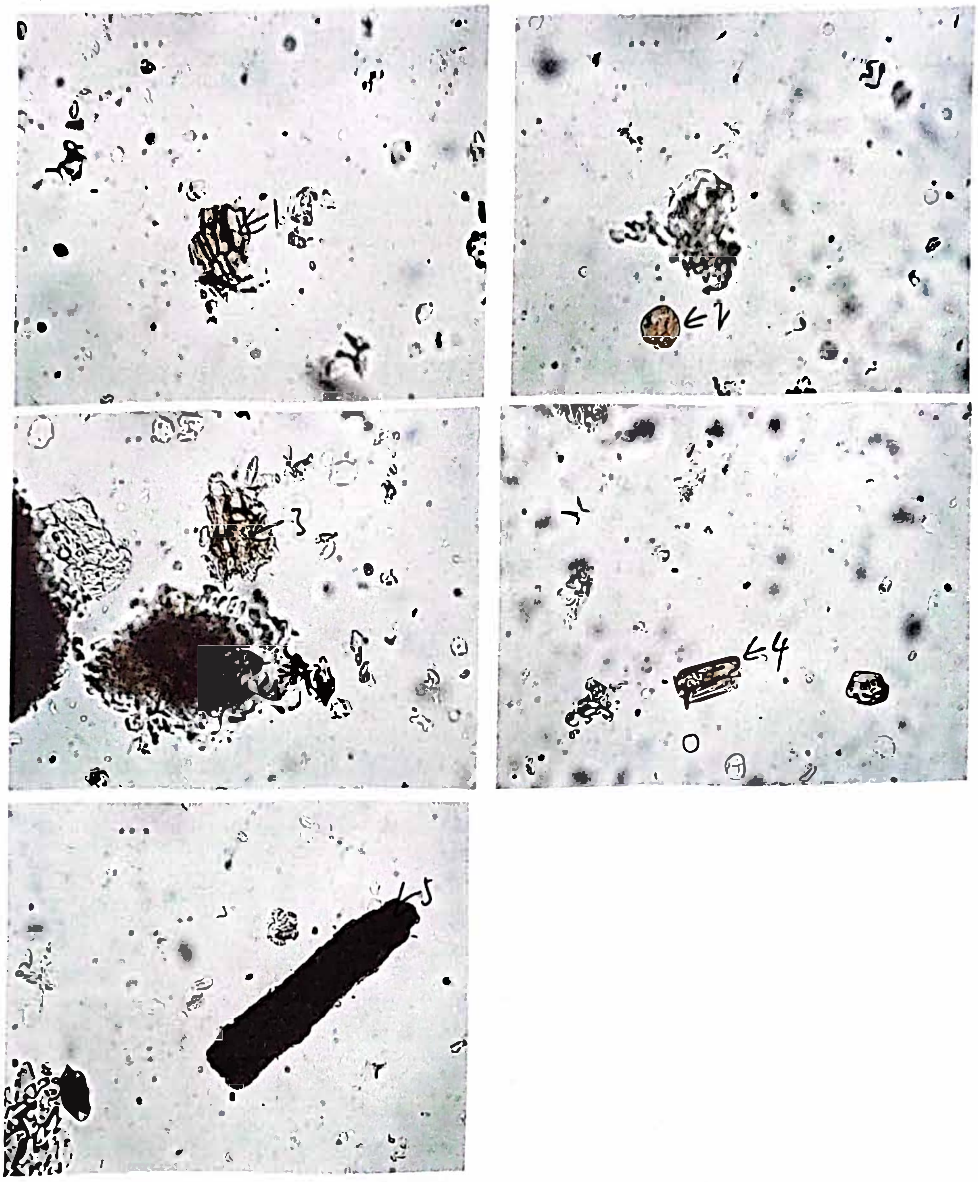

1.表皮细胞 2. 分泌细胞直径 40um3.厚壁细胞 4.石细胞 5. 下皮纤维直径 15um

Figure S6  Cyperus rotundus
