# Supplementary material for: Clinical efficacy of Bupleurum inula flower soup for immune damage intervention in Hashimoto’s thyroiditis: A placebo-controlled randomized trial
Source: Front Pharmacol. 2022 Nov 24;13:1049618. doi: 10.3389/fphar.2022.1049618 (PMC9730284; doi:10.3389/fphar.2022.1049618)
Supplement: Supplementary file 3 [file DataSheet4.pdf]

CAMAG®
World Leader in Planar Chromatography

[illegible] 010493-2110001

visionCATS

Take image clean plate 1a - Visualizer (S/N: 240188):

Executed	09-Nov-2021 14:27:43 尹思亲

R White

Clean, RemissionVis

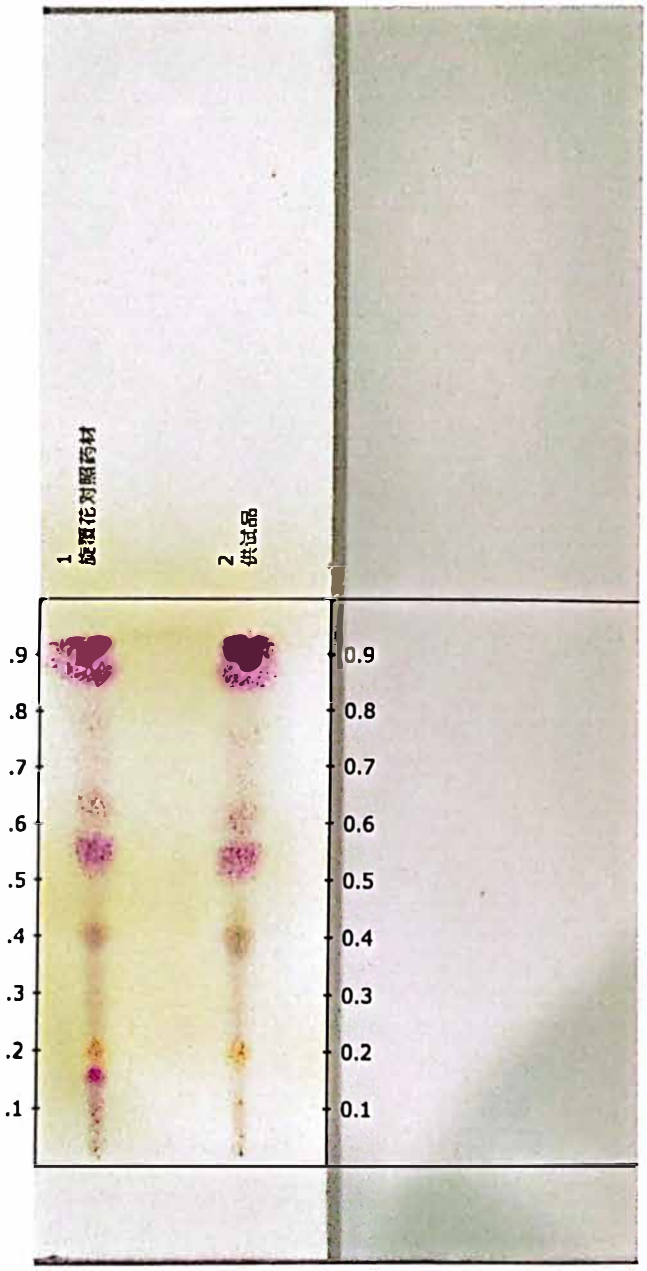

Exposure	0.152 s
Contrast	1
Normalized exposure	Disabled
Clarify	Disabled
White balance	1.00, 1.00, 1.00

Log:

旋覆花（旋覆花）010493-2110001

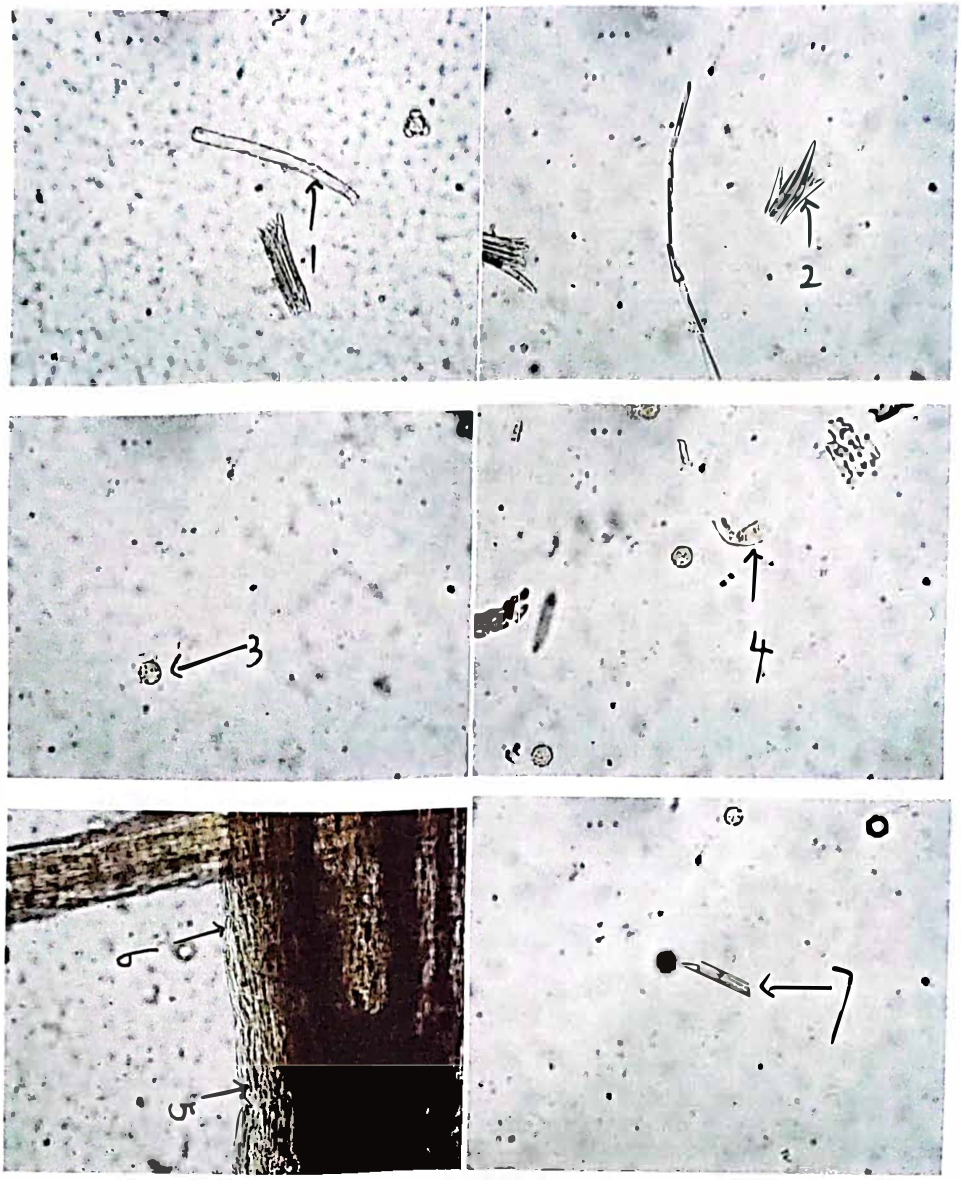

1.苞片非腺毛　2.冠毛非腺毛　3.花粉粒（直径 30um）　4.腺毛　5.子房表皮细胞
6.草酸钙柱晶（长 40um，柱晶 5um）　7.子房非腺毛（长 110um）

Figure S8 Inula
