# Supplementary figures and images for: Clinical efficacy of Bupleurum inula flower soup for immune damage intervention in Hashimoto’s thyroiditis: A placebo-controlled randomized trial
Source: Front Pharmacol. 2022 Nov 24;13:1049618. doi: 10.3389/fphar.2022.1049618 (PMC9730284; doi:10.3389/fphar.2022.1049618)

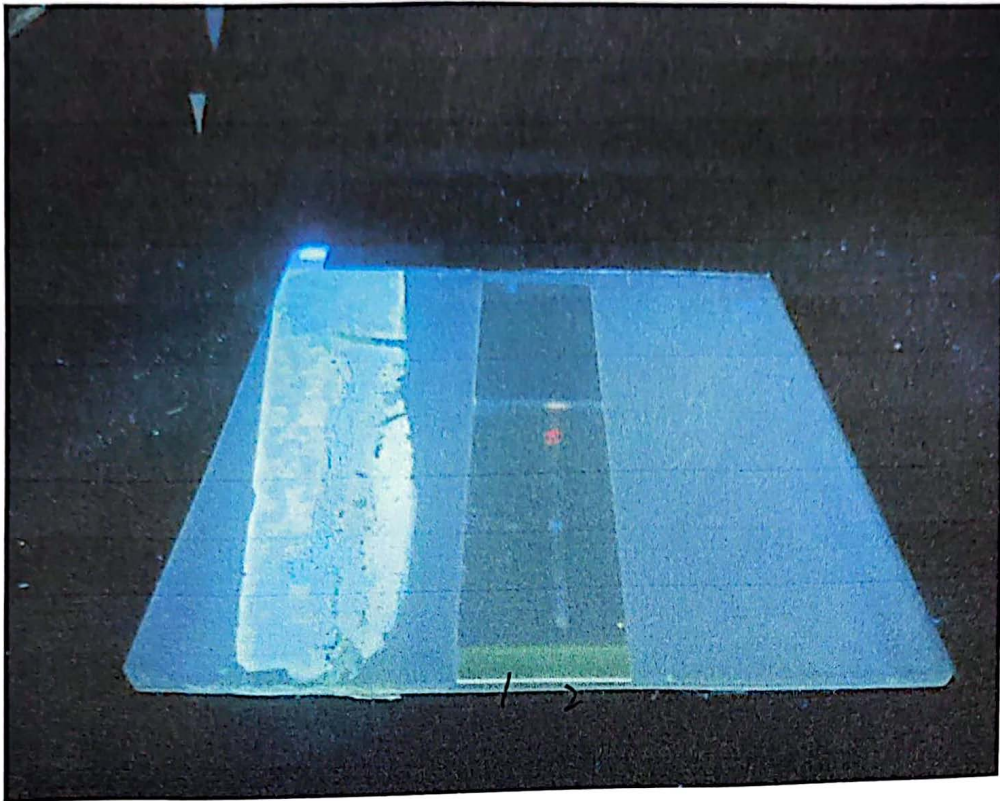

1. 对照品
2. 夏枯草供试品 010576-2209001

夏枯草 010576-2209001

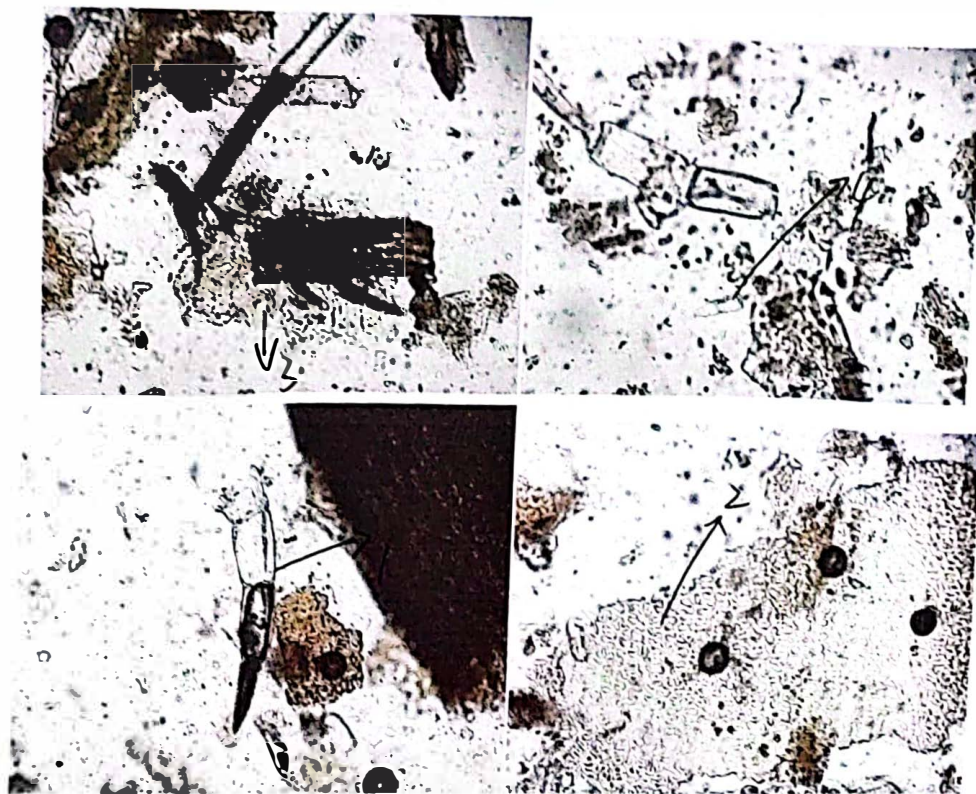

1.非腺毛 2.宿存花萼异形细胞 (直径 30um) 3.腺鳞 (直径 60um) 4.腺毛

Figure S10 Prunella

Supplement: Supplementary file 4 [file DataSheet6.pdf]

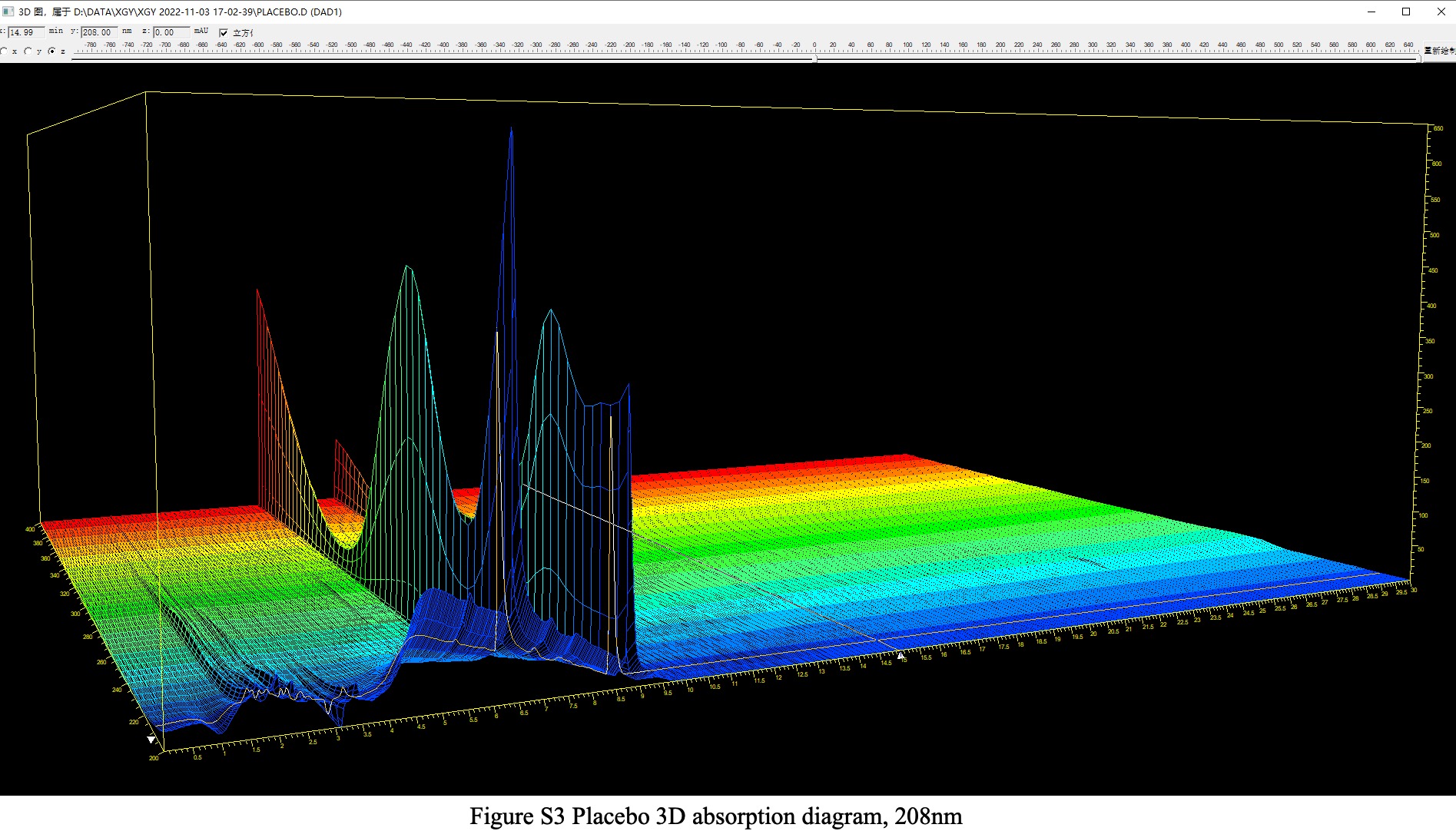

Supplement: Supplementary file 5 [file Image3.jpg]

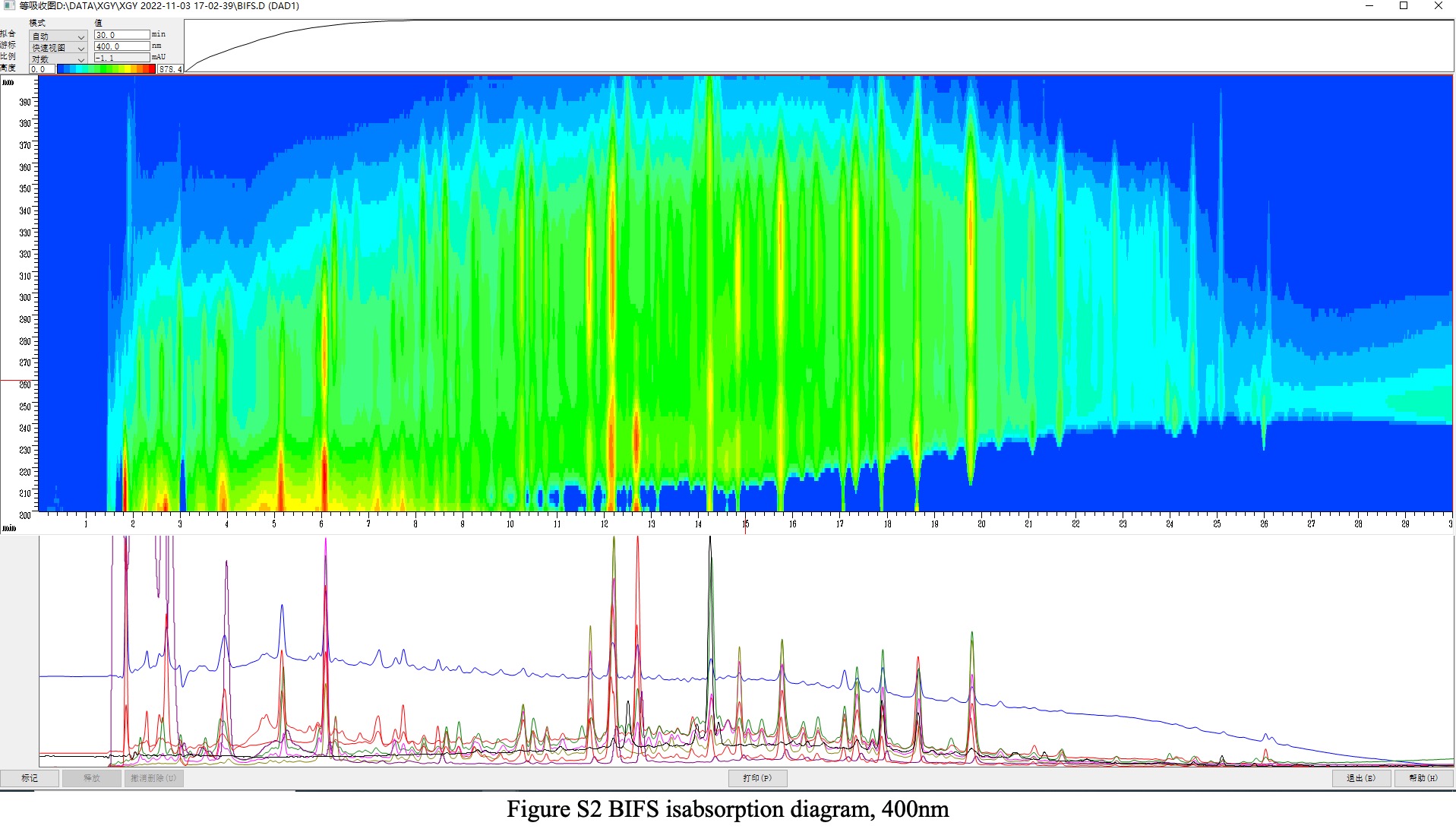

Supplement: Supplementary file 6 [file Image2.jpg]

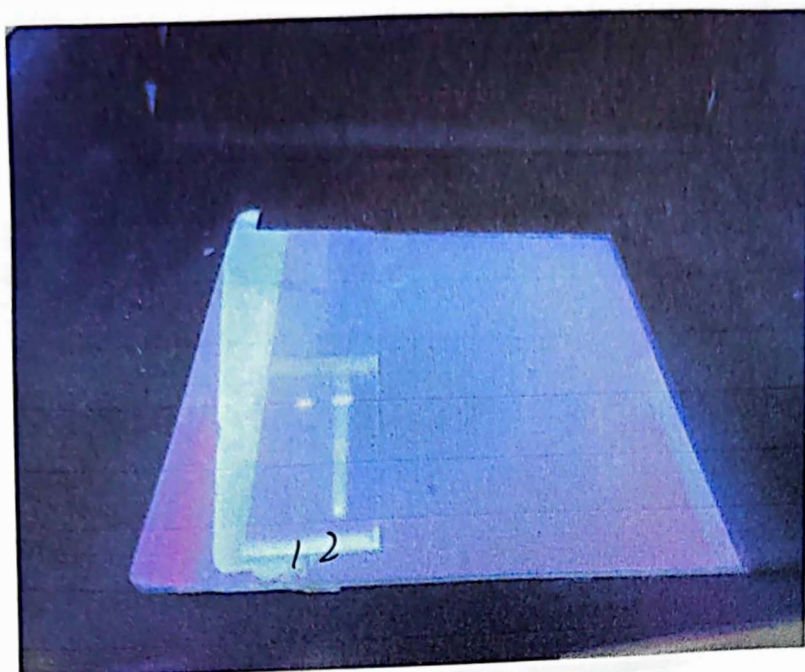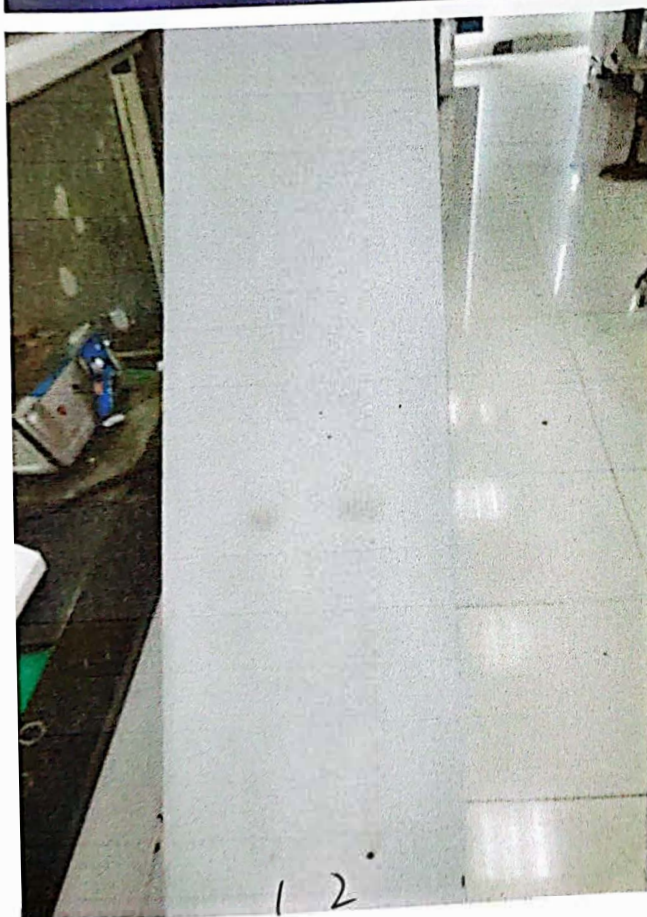

1. 对照品
2. 党参 (党参) 010110-2208001

党参 (党参) 010110-2208001

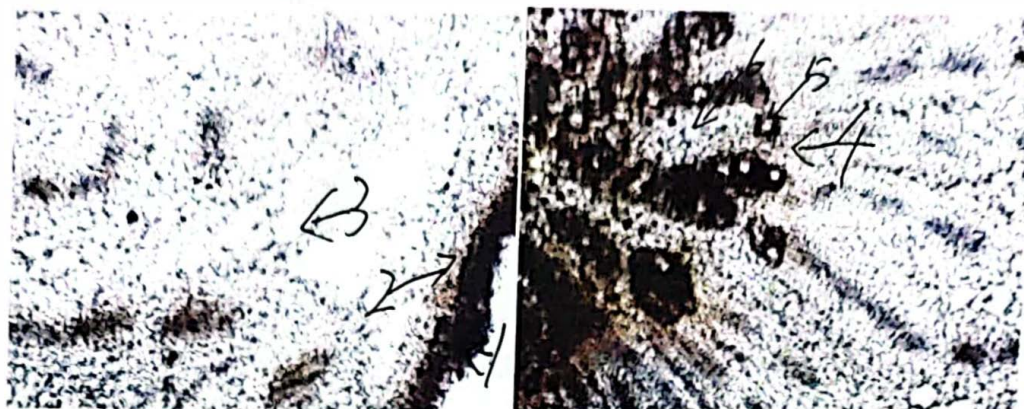

1.木栓细胞 2.栓内层 3.韧皮部 4.形成层 5.木质部导管 6.薄壁细胞

Figure S5 Codonopsis

Supplement: Supplementary file 10 [file DataSheet1.pdf]

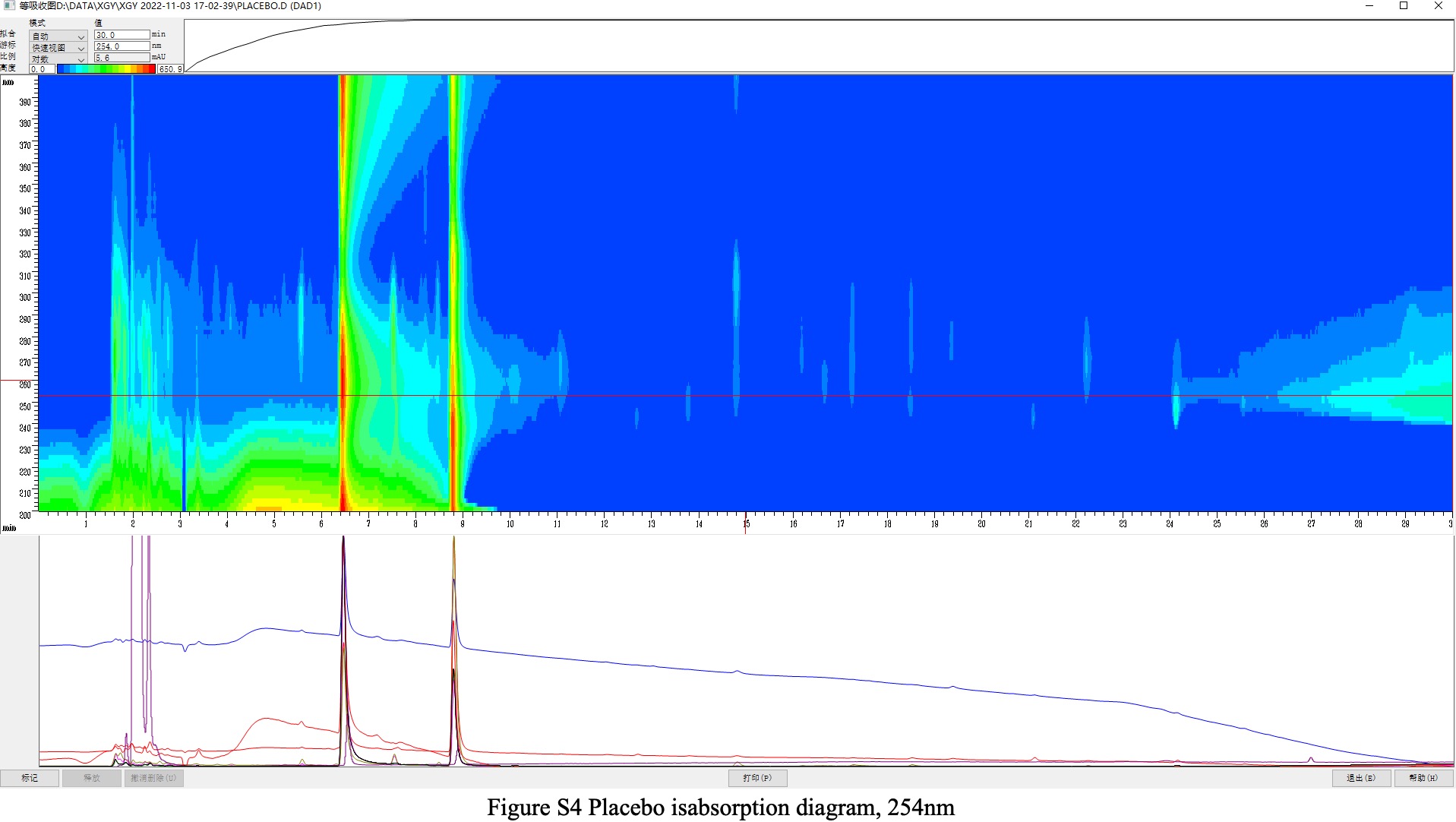

Supplement: Supplementary file 13 [file Image4.jpg]

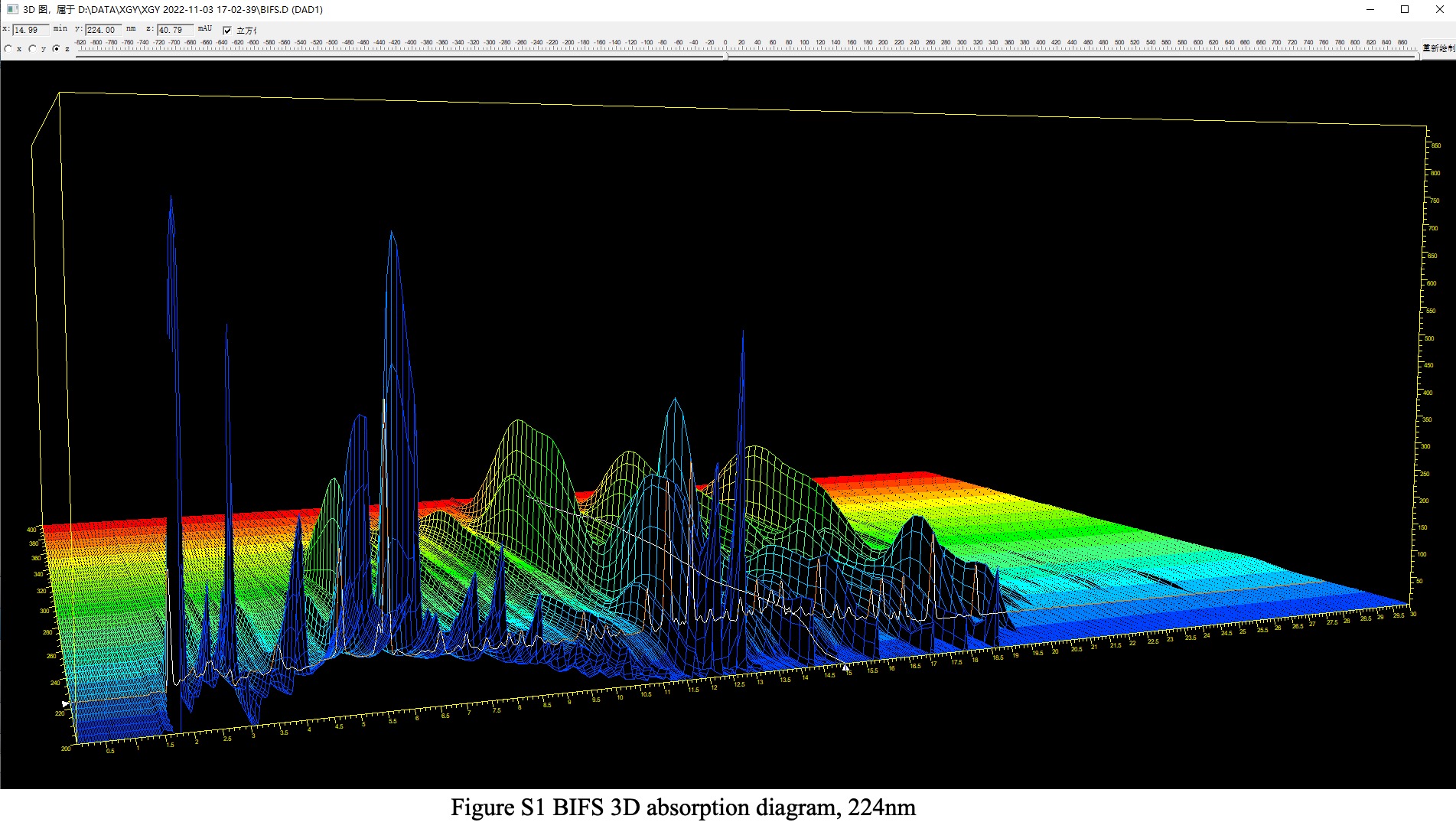

Supplement: Supplementary file 15 [file Image1.jpg]
