# Supplementary material for: Clinical efficacy of Bupleurum inula flower soup for immune damage intervention in Hashimoto’s thyroiditis: A placebo-controlled randomized trial
Source: Front Pharmacol. 2022 Nov 24;13:1049618. doi: 10.3389/fphar.2022.1049618 (PMC9730284; doi:10.3389/fphar.2022.1049618)
Supplement: Supplementary file 7 [file DataSheet9.pdf]

===

采集操作者 : 系统 序列行 : 1
样品操作者 : 系统
采集仪器 : LC-ELSD 位置 : P2-B-01
进样日期 : 2022/11/3 17:03:29 进样次数 : 1
进样量 : 5.000 µl
来自于样品输入的不同进样量! 实际进样量: 2.000 µl
采集方法 : D:\DATA\XGY\XGY 2022-11-03 17-02-39\DAD ELSD XGY.M
上次更改 : 2022/11/3 16:48:33 : 系统
分析方法 : D:\DATA\XGY\XGY 2022-11-03 17-02-39\DAD ELSD XGY.M (序列方法)
上次更改 : 2022/11/3 17:53:19 : 系统
(调用后修改)
附加信息：峰被手动积分

DAD1 A, Sig=205,4 Ref=off (D:\DATA\XGY\XGY 2022-11-03 17-02-39\BIFS.D)

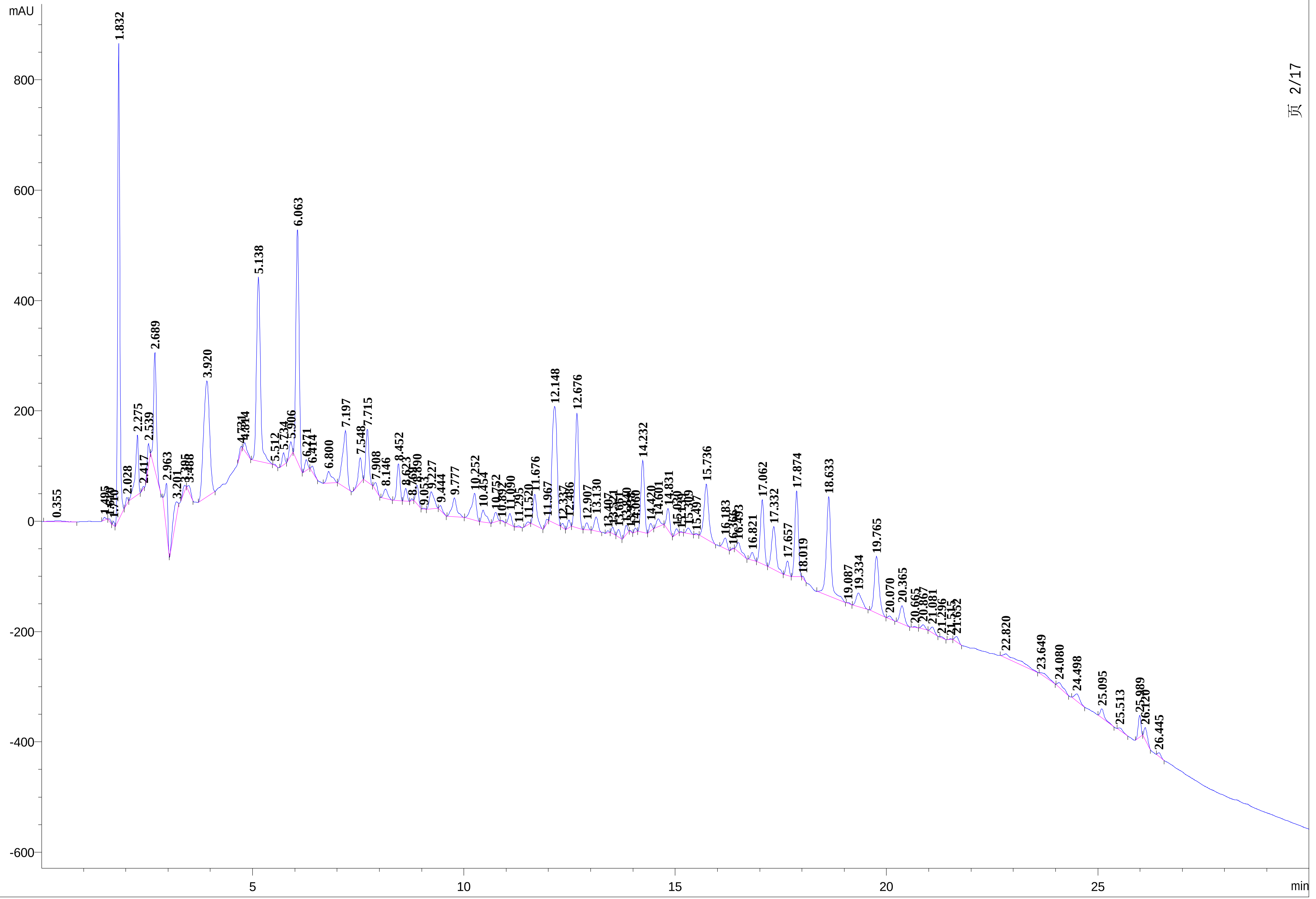

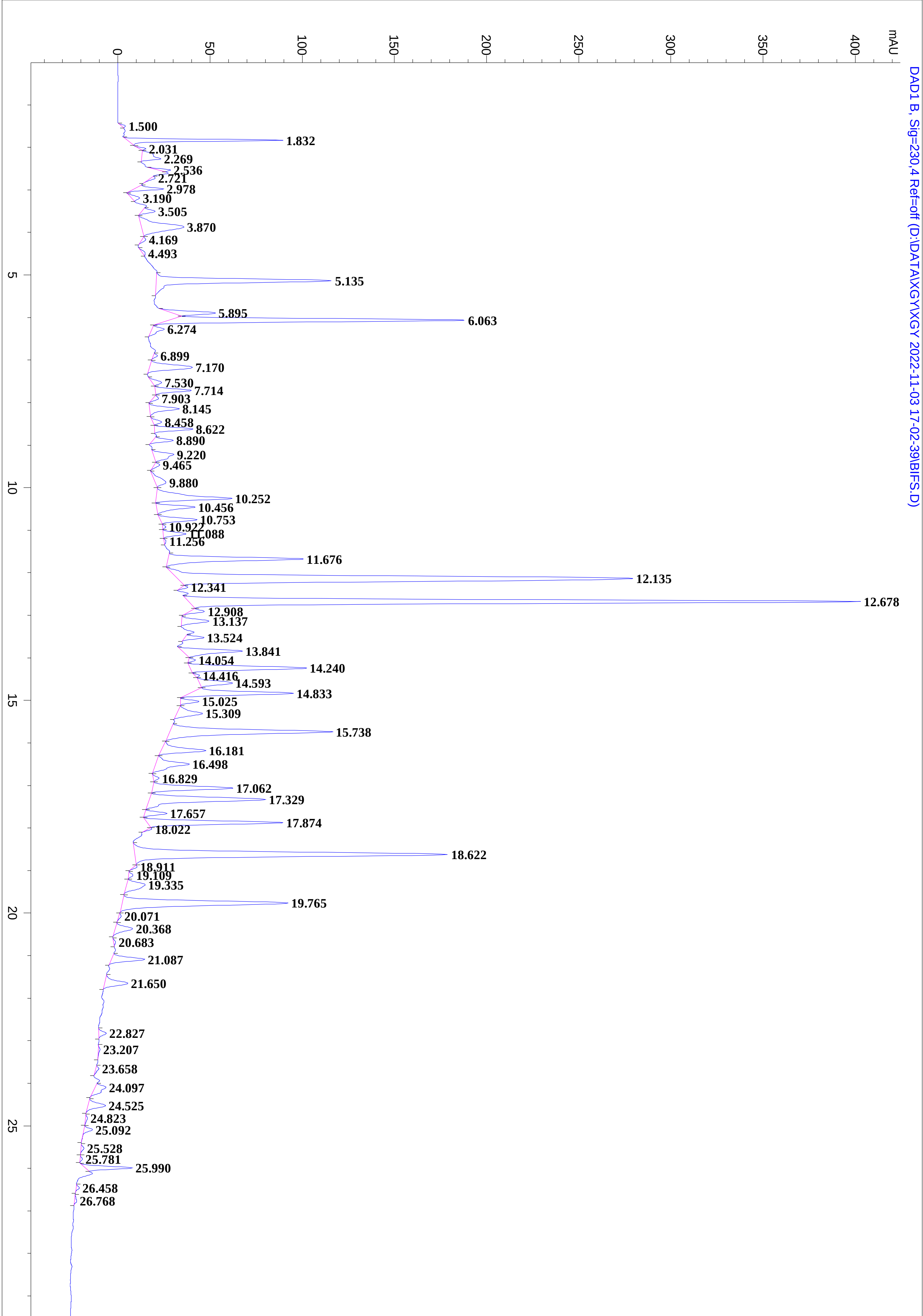

DAD1 B, Sig=230,4 Ref=off (D:\DATA\XGY\XGY 2022-11-03 17-02-39\BIFS.D)
mAU
0
50
100
150
200
250
300
350
400
5
10
15
20
25
min
1.500
1.832
2.031
2.269
2.536
2.721
2.978
3.190
3.505
3.870
4.169
4.493
5.135
5.895
6.063
6.274
6.899
7.170
7.530
7.714
7.903
8.145
8.458
8.622
8.890
9.220
9.465
9.880
10.252
10.456
10.753
10.922
11.088
11.256
11.676
12.135
12.341
12.678
12.908
13.137
13.524
13.841
14.054
14.240
14.416
14.593
14.833
15.025
15.309
15.738
16.181
16.498
16.829
17.062
17.329
17.657
17.874
18.022
18.622
18.911
19.109
19.335
19.765
20.071
20.368
20.683
21.087
21.650
22.827
23.207
23.658
24.097
24.525
24.823
25.092
25.528
25.781
25.990
26.458
26.768

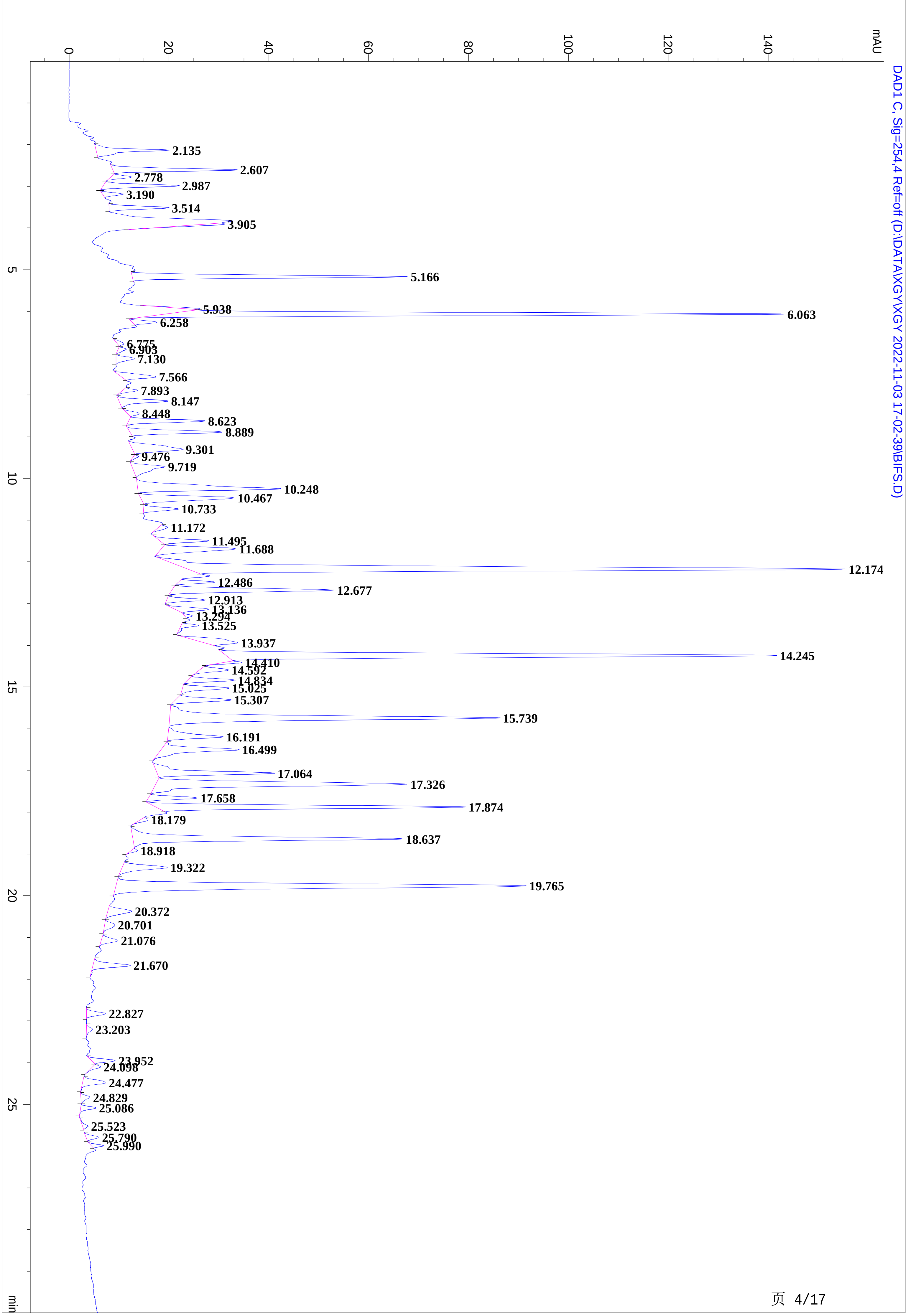
DAD1 C, Sig=254,4 Ref=off (D:\DATA\XGYXGY 2022-11-03 17-02-39\BIFS.D)
mAU
min
2.135
2.607
2.778
2.987
3.190
3.514
3.905
5.166
5.938
6.063
6.258
6.775
6.903
7.130
7.566
7.893
8.147
8.448
8.623
8.889
9.301
9.476
9.719
10.248
10.467
10.733
11.172
11.495
11.688
12.174
12.486
12.677
12.913
13.136
13.294
13.525
13.937
14.245
14.410
14.592
14.834
15.025
15.307
15.739
16.191
16.499
17.064
17.326
17.658
17.874
18.179
18.637
18.918
19.322
19.765
20.372
20.701
21.076
21.670
22.827
23.203
23.952
24.098
24.477
24.829
25.086
25.523
25.790
25.990

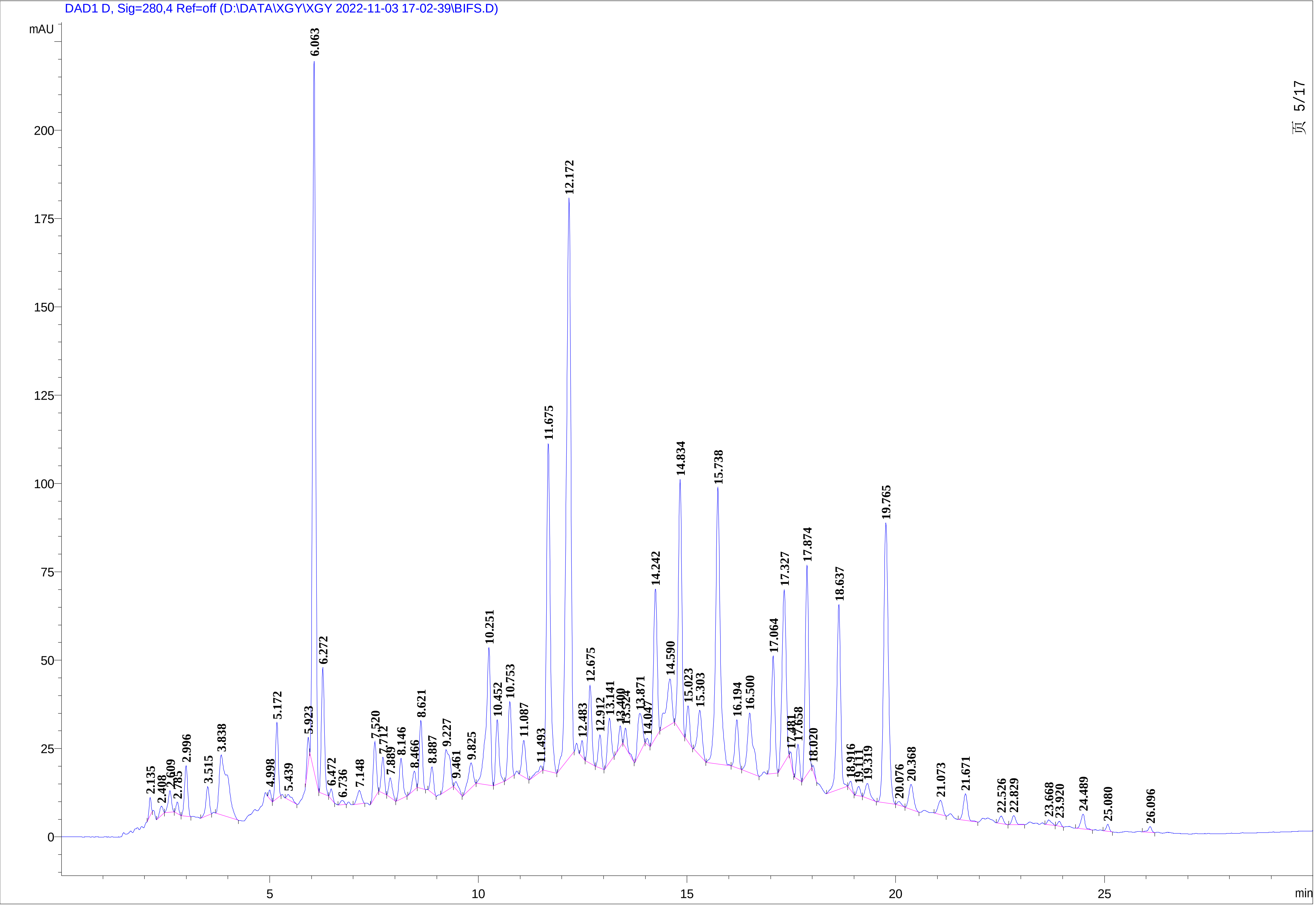

DAD1 D, Sig=280,4 Ref=off (D:\DATA\XGY\XGY 2022-11-03 17-02-39\BIFS.D)
mAU
200
175
150
125
100
75
50
25
0
5
10
15
20
25
min
2.135
2.408
2.609
2.785
2.996
3.515
3.838
4.998
5.172
5.439
5.923
6.063
6.272
6.472
6.736
7.148
7.520
7.712
7.889
8.146
8.466
8.621
8.887
9.227
9.461
9.825
10.251
10.452
10.753
11.087
11.493
11.675
12.172
12.483
12.675
12.912
13.141
13.400
13.524
13.871
14.047
14.242
14.590
14.834
15.023
15.303
15.738
16.194
16.500
17.064
17.327
17.481
17.658
17.874
18.020
18.637
18.916
19.111
19.319
19.765
20.076
20.368
21.073
21.671
22.526
22.829
23.668
23.920
24.489
25.080
26.096

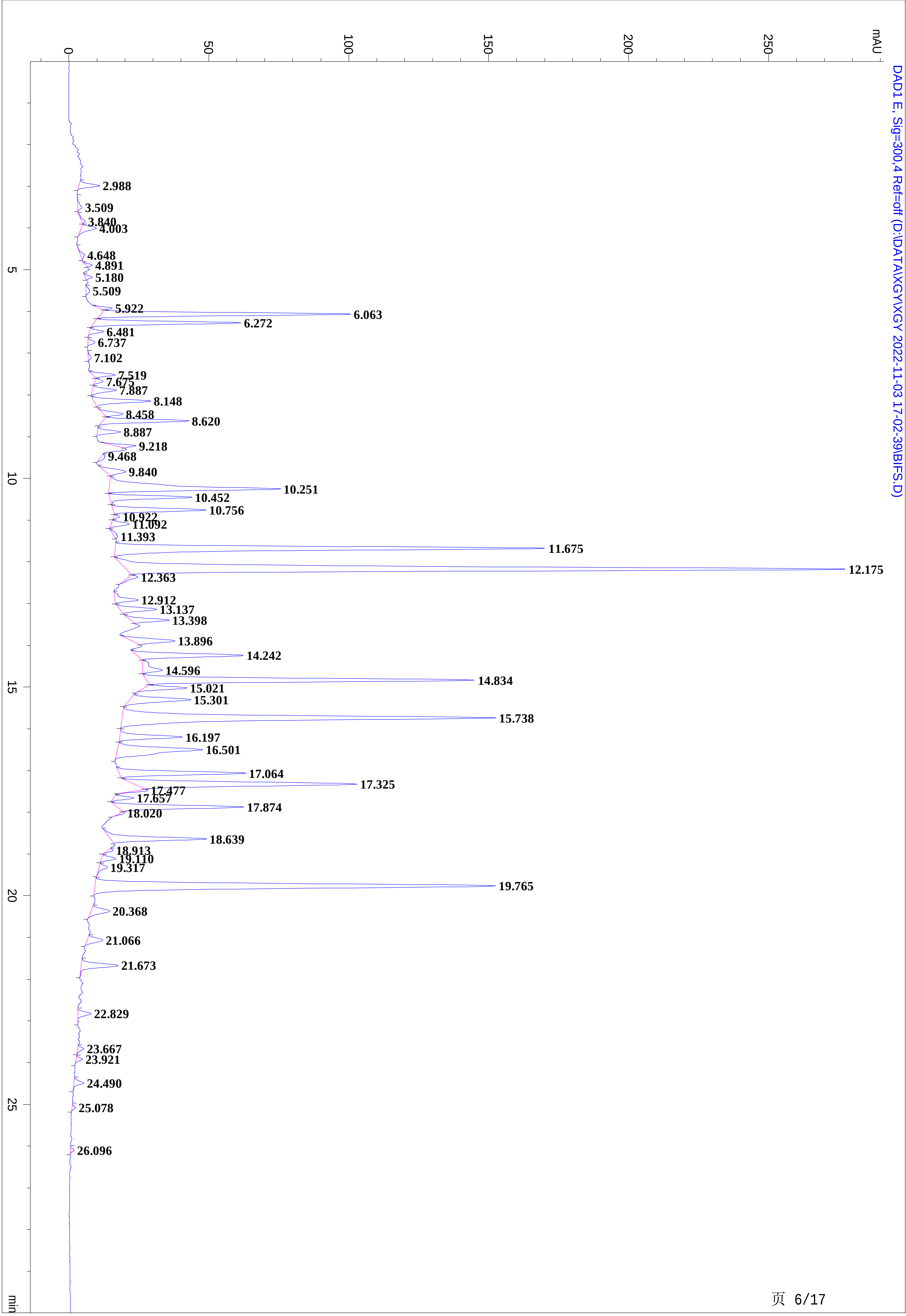
DAD1 E, Sig=300,4 Ref=off (D:\DATA\XGY\XGY 2022-11-03 17-02-39\BIFS.D)
mAU
0
50
100
150
200
250
min
5
10
15
20
25
2.988
3.509
3.840
4.003
4.648
4.891
5.180
5.509
5.922
6.063
6.272
6.481
6.737
7.102
7.519
7.675
7.887
8.148
8.458
8.620
8.887
9.218
9.468
9.840
10.251
10.452
10.756
10.922
11.092
11.393
11.675
12.175
12.363
12.912
13.137
13.398
13.896
14.242
14.596
14.834
15.021
15.301
15.738
16.197
16.501
17.064
17.325
17.477
17.657
17.874
18.020
18.639
18.913
19.110
19.317
19.765
20.368
21.066
21.673
22.829
23.667
23.921
24.490
25.078
26.096

ELS1 A, ELSD Signal (D:\DATA\XGY\XGY 2022-11-03 17-02-39\BIFS.D)

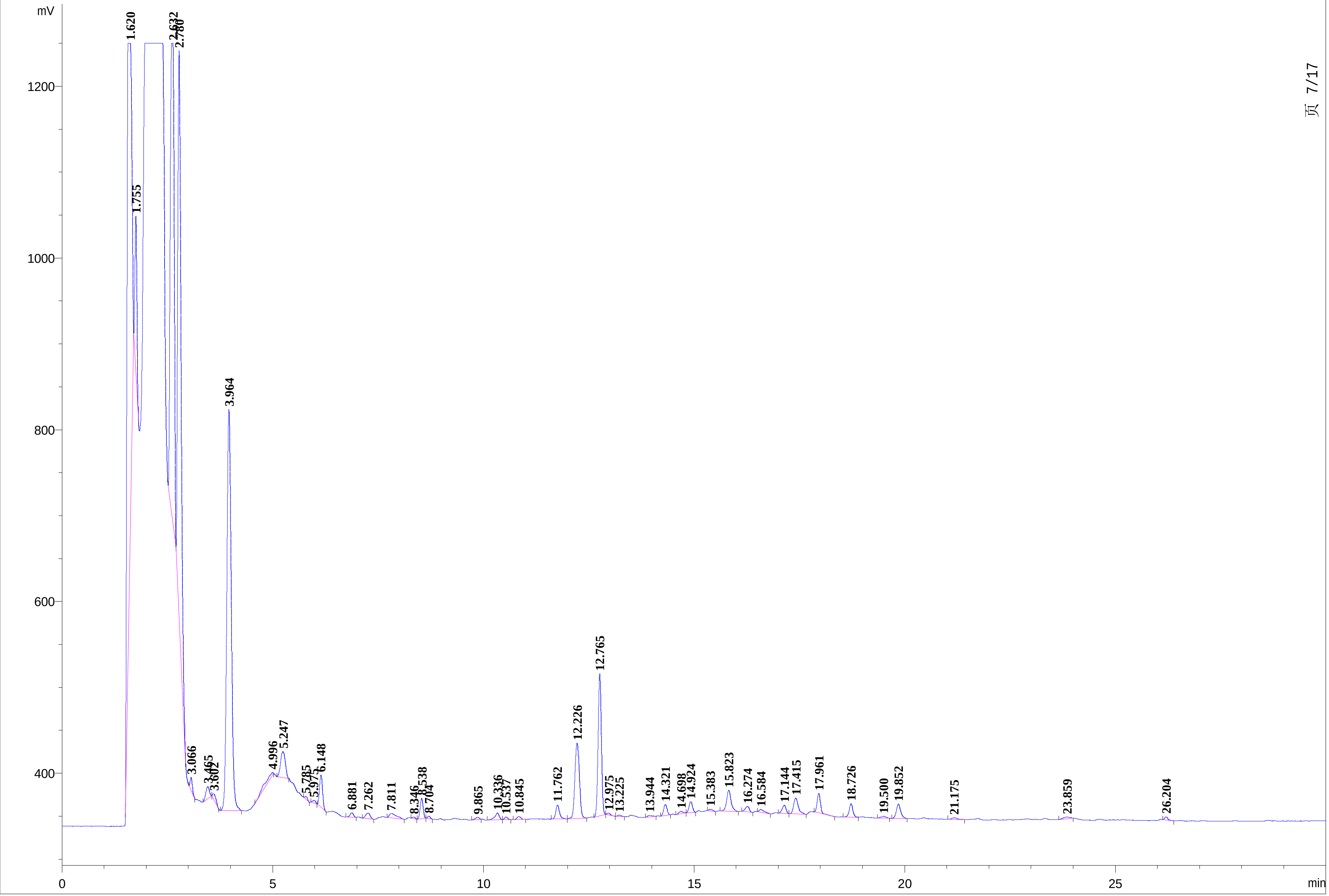

===

面积百分比报告

===

排序 : 信号
乘积因子 : 1.0000
稀释因子 : 1.0000
内标中不使用乘积因子和稀释因子

信号 1: DAD1 A, Sig=205,4 Ref=off

峰 #	保留时间 [min]	类型	峰宽 [min]	峰面积 [mAU*s]	峰高 [mAU]	峰面积 %
1	0.355	BB	0.2947	42.39295	1.77469	0.1468
2	1.495	BB	0.0640	21.21955	5.15322	0.0735
3	1.610	BB	0.0593	15.17460	4.08393	0.0526
4	1.710	BB	0.0423	18.38284	7.56664	0.0637
5	1.832	BB	0.0547	3011.77588	863.32660	10.4321
6	2.028	BB	0.0537	39.19698	12.12501	0.1358
7	2.275	BB	0.0748	568.85699	109.15788	1.9704
8	2.417	BB	0.0448	13.29857	4.73237	0.0461
9	2.539	BB	0.0589	142.32855	38.66883	0.4930
10	2.689	BB	0.0717	986.41547	214.29736	3.4167
11	2.963	BB	0.0690	400.19858	88.06245	1.3862
12	3.201	BB	0.1601	284.25763	27.61386	0.9846
13	3.395	BB	0.0809	54.16378	10.72087	0.1876
14	3.488	BB	0.0875	57.09997	10.16506	0.1978
15	3.920	BB	0.1612	2075.01758	210.19940	7.1874
16	4.731	BB	0.0706	42.26357	9.36907	0.1464
17	4.814	BB	0.0790	74.87705	14.33225	0.2594
18	5.138	BB	0.1016	2159.98218	333.50446	7.4817
19	5.512	BB	0.0740	7.95334	1.71988	0.0275
20	5.734	BB	0.0682	94.59719	21.98676	0.3277
21	5.906	BB	0.0785	118.32918	25.34742	0.4099
22	6.063	BB	0.0799	2165.57275	421.49185	7.5011
23	6.271	BB	0.0752	93.73332	19.79800	0.3247
24	6.414	BB	0.0723	50.55098	10.87095	0.1751
25	6.800	BB	0.1166	175.46733	21.19257	0.6078
26	7.197	BB	0.1082	787.99280	104.23132	2.7294
27	7.548	BB	0.0813	241.96259	46.04881	0.8381
28	7.715	BB	0.0801	493.85977	95.88688	1.7106
29	7.908	BB	0.0828	70.82088	13.57874	0.2453
30	8.146	BB	0.1054	120.72354	17.73426	0.4182
31	8.452	BB	0.0815	340.99661	66.85750	1.1811
32	8.623	BB	0.0766	107.37852	22.94449	0.3719
33	8.769	BB	0.0514	10.98671	3.61822	0.0381
34	8.890	BB	0.0795	169.42442	34.37950	0.5868
35	9.053	BB	0.0610	6.53086	1.85931	0.0226
36	9.227	BB	0.1124	237.84618	30.67862	0.8238
37	9.444	BB	0.0788	42.93112	8.51141	0.1487
38	9.777	BB	0.1185	284.31693	34.37614	0.9848
39	10.252	BB	0.1143	385.63025	48.75177	1.3357

峰 #	保留时间 [min]	类型	峰宽 [min]	峰面积 [mAU*s]	峰高 [mAU]	峰面积 %
40	10.454	BB	0.1088	163.62927	21.49723	0.5668
41	10.752	BB	0.0890	97.51440	17.51102	0.3378
42	10.892	BB	0.0885	7.07731	1.27929	0.0245
43	11.090	BB	0.0914	113.38839	20.24121	0.3928
44	11.295	BB	0.0960	13.57307	2.47270	0.0470
45	11.520	BB	0.0946	25.92439	4.68486	0.0898
46	11.676	BB	0.0924	348.20764	56.08199	1.2061
47	11.967	BB	0.0683	27.12046	6.55617	0.0939
48	12.148	BB	0.1317	1699.95801	211.72168	5.8883
49	12.337	BB	0.0644	28.73602	7.54802	0.0995
50	12.486	BB	0.0660	53.86140	13.64573	0.1866
51	12.676	BB	0.0945	1260.27185	208.54007	4.3653
52	12.907	BB	0.0801	62.72935	12.59027	0.2173
53	13.130	BB	0.0970	159.47948	25.49520	0.5524
54	13.407	BB	0.0574	13.28289	3.93274	0.0460
55	13.521	BB	0.0670	47.08779	11.69729	0.1631
56	13.661	BB	0.0742	62.05379	13.87439	0.2149
57	13.840	BB	0.0732	76.38667	16.75285	0.2646
58	13.938	BB	0.0474	5.78959	2.02256	0.0201
59	14.060	BB	0.0595	25.48954	7.17406	0.0883
60	14.232	BB	0.0932	751.06952	130.32941	2.6015
61	14.420	BB	0.0750	59.56796	13.12323	0.2063
62	14.601	BB	0.0997	88.51067	14.01278	0.3066
63	14.831	BB	0.0855	206.71640	39.23190	0.7160
64	15.030	BB	0.0746	44.17751	9.80352	0.1530
65	15.139	BB	0.0525	5.42610	1.73231	0.0188
66	15.309	BB	0.0996	62.72045	10.21388	0.2172
67	15.497	BB	0.0655	8.73277	2.23815	0.0302
68	15.736	BB	0.1167	778.28485	100.11404	2.6958
69	16.183	BB	0.0984	123.60049	19.38210	0.4281
70	16.366	BB	0.0645	6.78369	1.70299	0.0235
71	16.493	BB	0.1105	122.74691	16.55968	0.4252
72	16.821	BB	0.0852	77.38226	14.28864	0.2680
73	17.062	BB	0.0960	719.29724	116.55522	2.4915
74	17.332	BB	0.1170	580.51984	77.86378	2.0108
75	17.657	BB	0.0815	134.74219	26.40828	0.4667
76	17.874	BB	0.0910	891.22028	155.19493	3.0870
77	18.019	BB	0.0676	14.26503	3.35707	0.0494
78	18.633	BB	0.1170	1401.82751	179.74352	4.8556
79	19.087	BB	0.0843	7.55999	1.46210	0.0262
80	19.334	BB	0.1384	235.46312	24.88995	0.8156
81	19.765	BB	0.1197	808.42841	102.88374	2.8002
82	20.070	BB	0.0917	28.44635	4.90096	0.0985
83	20.365	BB	0.1142	241.61429	32.68378	0.8369
84	20.665	BB	0.0808	9.17886	1.82186	0.0318
85	20.867	BB	0.0970	45.78054	7.52391	0.1586
86	21.081	BB	0.1041	67.31836	10.32303	0.2332
87	21.296	BB	0.0937	9.29260	1.55650	0.0322
88	21.515	BB	0.0686	5.49060	1.31695	0.0190
89	21.652	BB	0.1017	58.76428	9.30245	0.2035
90	22.820	BB	0.4055	238.10538	7.35882	0.8247

峰 #	保留时间 [min]	类型	峰宽 [min]	峰面积 [mAU*s]	峰高 [mAU]	峰面积 %
91	23.649	BB	0.3423	62.18058	2.22336	0.2154
92	24.080	BB	0.1524	84.13884	7.89259	0.2914
93	24.498	BB	0.1334	111.75561	13.11496	0.3871
94	25.095	BB	0.0966	104.60410	16.35644	0.3623
95	25.513	BB	0.1150	31.91423	4.18482	0.1105
96	25.989	BB	0.0722	178.22733	39.83092	0.6173
97	26.120	BB	0.0937	125.43707	22.26923	0.4345
98	26.445	BBA	0.0818	32.85084	6.40590	0.1138

总量 : 2.88702e4 4932.19538

信号 2: DAD1 B, Sig=230,4 Ref=off

峰 #	保留时间 [min]	类型	峰宽 [min]	峰面积 [mAU*s]	峰高 [mAU]	峰面积 %
1	1.500	BB	0.0563	7.56943	2.08667	0.0502
2	1.832	BB	0.0539	288.79095	84.52254	1.9154
3	2.031	BB	0.0523	11.64367	3.54424	0.0772
4	2.269	BB	0.1128	85.70699	10.54806	0.5684
5	2.536	BB	0.0577	21.75764	6.08557	0.1443
6	2.721	BB	0.0732	11.19240	2.36716	0.0742
7	2.978	BB	0.0671	70.18060	16.02485	0.4655
8	3.190	BB	0.1037	26.95844	4.38493	0.1788
9	3.505	BB	0.0855	38.12744	7.23196	0.2529
10	3.870	BB	0.1832	280.88278	23.21473	1.8629
11	4.169	BB	0.1029	12.94381	2.07251	0.0858
12	4.493	BB	0.1201	9.83118	1.30263	0.0652
13	5.135	BB	0.0991	613.28662	95.31789	4.0675
14	5.895	BB	0.0873	122.06020	23.22343	0.8095
15	6.063	BB	0.0796	819.09888	160.46648	5.4325
16	6.274	BB	0.1047	50.26309	6.76355	0.3334
17	6.899	BB	0.0745	11.23259	2.40433	0.0745
18	7.170	BB	0.1296	185.99942	23.19200	1.2336
19	7.530	BB	0.1040	32.31263	5.09383	0.2143
20	7.714	BB	0.0828	101.85383	19.53957	0.6755
21	7.903	BB	0.0946	17.37534	2.95636	0.1152
22	8.145	BB	0.0994	103.77183	16.06412	0.6882
23	8.458	BB	0.0911	25.78036	4.62362	0.1710
24	8.622	BB	0.0811	105.10178	20.73059	0.6971
25	8.890	BB	0.0798	55.07143	11.11452	0.3653
26	9.220	BB	0.1224	98.37071	11.20708	0.6524
27	9.465	BB	0.0918	18.08256	3.02209	0.1199
28	9.880	BB	0.1662	66.97367	5.82040	0.4442
29	10.252	BB	0.1059	302.75388	41.12361	2.0080
30	10.456	BB	0.0962	133.44601	20.97161	0.8851
31	10.753	BB	0.0922	113.18294	19.94534	0.7507
32	10.922	BB	0.0653	6.74376	1.73529	0.0447
33	11.088	BB	0.0895	68.64008	12.22257	0.4552

峰 #	保留时间 [min]	类型	峰宽 [min]	峰面积 [mAU*s]	峰高 [mAU]	峰面积 %
34	11.256	BB	0.0888	5.39726	1.07012	0.0358
35	11.676	BB	0.0948	459.43433	73.60674	3.0471
36	12.135	BB	0.1350	2054.63721	247.23184	13.6270
37	12.341	BB	0.0642	12.47158	3.29234	0.0827
38	12.678	BB	0.0917	2179.52173	364.62222	14.4553
39	12.908	BB	0.0774	38.25878	8.05161	0.2537
40	13.137	BB	0.1000	94.95010	14.96390	0.6297
41	13.524	BB	0.0739	46.56949	10.46410	0.3089
42	13.841	BB	0.0963	208.43076	32.71993	1.3824
43	14.054	BB	0.0633	13.60748	3.67036	0.0902
44	14.240	BB	0.0961	379.92136	63.24660	2.5198
45	14.416	BB	0.0620	8.81328	2.44852	0.0585
46	14.593	BB	0.1058	115.91425	17.83268	0.7688
47	14.833	BB	0.0899	317.44464	56.20996	2.1054
48	15.025	BB	0.0785	48.66907	10.04571	0.3228
49	15.309	BB	0.1232	114.50545	14.02838	0.7594
50	15.738	BB	0.1122	668.21417	88.37401	4.4318
51	16.181	BB	0.1098	174.96925	24.35531	1.1605
52	16.498	BB	0.1320	169.69409	18.32737	1.1255
53	16.829	BB	0.0849	16.62318	3.18264	0.1103
54	17.062	BB	0.0955	267.56784	43.65868	1.7746
55	17.329	BB	0.1171	481.71945	63.08475	3.1949
56	17.657	BB	0.0816	62.12226	12.16271	0.4120
57	17.874	BB	0.0909	422.38245	73.66093	2.8014
58	18.022	BB	0.0642	7.43976	1.87848	0.0493
59	18.622	BB	0.1156	1275.91479	169.98123	8.4623
60	18.911	BB	0.0787	8.53769	1.75651	0.0566
61	19.109	BB	0.0872	12.41190	2.36551	0.0823
62	19.335	BB	0.1495	108.56253	10.09001	0.7200
63	19.765	BB	0.1207	699.64093	90.00140	4.6402
64	20.071	BB	0.0926	7.62199	1.25961	0.0506
65	20.368	BB	0.1203	74.71959	9.44814	0.4956
66	20.683	BB	0.0975	6.75383	1.10208	0.0448
67	21.087	BB	0.1005	118.66165	18.10590	0.7870
68	21.650	BB	0.1232	101.15258	12.65849	0.6709
69	22.827	BB	0.0995	25.50569	4.05097	0.1692
70	23.207	BB	0.1266	8.87890	1.02997	0.0589
71	23.658	BB	0.1012	11.61022	1.66781	0.0770
72	24.097	BB	0.1517	64.81693	6.01397	0.4299
73	24.525	BB	0.1220	75.35707	9.55527	0.4998
74	24.823	BB	0.1123	8.18338	1.08176	0.0543
75	25.092	BB	0.0884	25.98542	4.56781	0.1723
76	25.528	BB	0.1005	11.10804	1.69426	0.0737
77	25.781	BB	0.0798	6.10814	1.23321	0.0405
78	25.990	BB	0.0760	121.60357	25.33246	0.8065
79	26.458	BB	0.0828	9.59523	1.84238	0.0636
80	26.768	BB	0.1022	8.66643	1.22948	0.0575

总量 : 1.50777e4 2299.18227

信号 3: DAD1 C, Sig=254,4 Ref=off

峰 #	保留时间 [min]	类型	峰宽 [min]	峰面积 [mAU*s]	峰高 [mAU]	峰面积 %
1	2.135	BB	0.0840	86.16139	14.77676	1.1111
2	2.607	BB	0.0790	125.82726	24.89637	1.6226
3	2.778	BB	0.0821	20.74263	4.16424	0.2675
4	2.987	BB	0.0801	78.29832	15.19511	1.0097
5	3.190	BB	0.0761	19.83908	4.12479	0.2558
6	3.514	BB	0.0822	61.81519	11.97318	0.7972
7	3.905	BB	0.0928	23.74126	3.80346	0.3062
8	5.166	BB	0.0800	283.63419	55.14694	3.6577
9	5.938	BB	0.0735	12.39906	2.60648	0.1599
10	6.063	BB	0.0792	629.64136	124.14840	8.1197
11	6.258	BB	0.0813	23.78852	5.01744	0.3068
12	6.775	BB	0.1095	8.34949	1.32587	0.1077
13	6.903	BB	0.0860	9.56479	1.64187	0.1233
14	7.130	BB	0.1001	24.05450	3.68657	0.3102
15	7.566	BB	0.1158	47.37444	6.92697	0.6109
16	7.893	BB	0.0770	15.00863	3.07000	0.1935
17	8.147	BB	0.0945	60.88812	9.80313	0.7852
18	8.448	BB	0.1023	14.20131	2.35634	0.1831
19	8.623	BB	0.0862	81.71381	15.32454	1.0538
20	8.889	BB	0.0888	103.11069	18.55585	1.3297
21	9.301	BB	0.1396	96.28706	10.06276	1.2417
22	9.476	BB	0.0791	5.41388	1.03409	0.0698
23	9.719	BB	0.1308	62.14249	6.66414	0.8014
24	10.248	BB	0.1133	224.46773	28.67045	2.8947
25	10.467	BB	0.1152	134.09138	18.81378	1.7292
26	10.733	BB	0.0910	40.16056	6.99181	0.5179
27	11.172	BB	0.1104	11.92305	1.82095	0.1538
28	11.495	BB	0.0880	53.62921	9.77966	0.6916
29	11.688	BB	0.1236	112.48767	14.97494	1.4506
30	12.174	BB	0.1059	924.15857	131.65761	11.9177
31	12.486	BB	0.0692	30.87511	7.31742	0.3982
32	12.677	BB	0.0914	187.78801	32.50509	2.4217
33	12.913	BB	0.0819	39.64278	7.72695	0.5112
34	13.136	BB	0.0904	37.35366	6.76821	0.4817
35	13.294	BB	0.0651	5.87386	1.51940	0.0757
36	13.525	BB	0.0794	18.48794	3.51399	0.2384
37	13.937	BB	0.1330	64.97567	6.71039	0.8379
38	14.245	BB	0.1004	702.83136	110.24030	9.0636
39	14.410	BB	0.0621	13.92925	3.68168	0.1796
40	14.592	BB	0.0983	34.90152	5.79543	0.4501
41	14.834	BB	0.0848	49.43462	9.49140	0.6375
42	15.025	BB	0.0903	54.68845	9.33753	0.7052
43	15.307	BB	0.1027	71.15823	11.11015	0.9176
44	15.739	BB	0.1154	507.37106	66.17928	6.5429
45	16.191	BB	0.1096	81.27144	11.07682	1.0481
46	16.499	BB	0.1173	122.01823	15.59597	1.5735
47	17.064	BB	0.1020	157.41110	23.55396	2.0299
48	17.326	BB	0.1178	379.09900	50.35550	4.8888

峰 #	保留时间 [min]	类型	峰宽 [min]	峰面积 [mAU*s]	峰高 [mAU]	峰面积 %
49	17.658	BB	0.0818	50.50392	9.84878	0.6513
50	17.874	BB	0.0910	356.15829	62.03532	4.5929
51	18.179	BB	0.0975	9.13226	1.53238	0.1178
52	18.637	BB	0.1020	361.78577	54.11402	4.6655
53	18.918	BB	0.0824	6.40671	1.28094	0.0826
54	19.322	BB	0.1200	71.27100	9.03567	0.9191
55	19.765	BB	0.1209	639.93573	82.16563	8.2525
56	20.372	BB	0.1361	41.85543	4.78344	0.5398
57	20.701	BB	0.1564	19.52847	2.06316	0.2518
58	21.076	BB	0.1196	25.28765	3.36609	0.3261
59	21.670	BB	0.1082	52.51503	7.44958	0.6772
60	22.827	BB	0.0999	24.38752	3.84997	0.3145
61	23.203	BB	0.1143	9.53445	1.25924	0.1230
62	23.952	BB	0.0827	24.68368	4.74389	0.3183
63	24.098	BB	0.0937	9.27076	1.59812	0.1196
64	24.477	BB	0.1233	35.73255	4.66640	0.4608
65	24.829	BB	0.1047	12.97793	1.82989	0.1674
66	25.086	BB	0.0861	17.00262	3.09287	0.2193
67	25.523	BB	0.1006	7.34233	1.08931	0.0947
68	25.790	BB	0.0900	14.61140	2.58403	0.1884
69	25.990	BB	0.0707	10.52842	2.42370	0.1358

总量 : 7754.47888 1206.30642

信号 4: DAD1 D, Sig=280,4 Ref=off

峰 #	保留时间 [min]	类型	峰宽 [min]	峰面积 [mAU*s]	峰高 [mAU]	峰面积 %
1	2.135	BB	0.0536	17.77615	5.23924	0.1935
2	2.408	BB	0.0906	14.32315	2.58770	0.1559
3	2.609	BB	0.0826	34.03430	6.15036	0.3705
4	2.785	BB	0.0715	14.68948	3.32740	0.1599
5	2.996	BB	0.0814	75.40809	14.33906	0.8210
6	3.515	BB	0.0860	45.87075	8.11402	0.4994
7	3.838	BB	0.1881	239.04137	16.99450	2.6024
8	4.998	BB	0.0611	9.05738	2.44954	0.0986
9	5.172	BB	0.0742	103.58176	21.50872	1.1277
10	5.439	BB	0.1143	11.67938	1.41523	0.1272
11	5.923	BB	0.0569	27.35698	7.79905	0.2978
12	6.063	BB	0.0798	1031.47095	201.35141	11.2296
13	6.272	BB	0.0809	180.74338	35.78705	1.9677
14	6.472	BB	0.0696	11.92575	2.80367	0.1298
15	6.736	BB	0.1035	7.29408	1.19027	0.0794
16	7.148	BB	0.1044	27.71906	3.83078	0.3018
17	7.520	BB	0.0818	80.69660	15.75339	0.8785
18	7.712	BB	0.0802	50.99134	10.21710	0.5551
19	7.889	BB	0.0911	32.76191	5.52913	0.3567
20	8.146	BB	0.0933	70.52906	11.54396	0.7678

峰 #	保留时间 [min]	类型	峰宽 [min]	峰面积 [mAU*s]	峰高 [mAU]	峰面积 %
21	8.466	BB	0.0887	29.45604	5.31095	0.3207
22	8.621	BB	0.0806	96.50963	19.22563	1.0507
23	8.887	BB	0.0845	38.36713	7.39889	0.4177
24	9.227	BB	0.1314	111.51019	11.67361	1.2140
25	9.461	BB	0.1001	14.36919	2.09173	0.1564
26	9.825	BB	0.1278	59.56745	7.10238	0.6485
27	10.251	BB	0.1106	295.72168	38.94463	3.2195
28	10.452	BB	0.0863	100.50480	18.24763	1.0942
29	10.753	BB	0.0904	123.07836	21.63835	1.3399
30	11.087	BB	0.1006	64.08865	10.30287	0.6977
31	11.493	BB	0.1066	12.55243	1.65233	0.1367
32	11.675	BB	0.0942	575.08105	92.91995	6.2609
33	12.172	BB	0.1111	1183.12549	158.55139	12.8806
34	12.483	BB	0.0650	18.51936	4.59966	0.2016
35	12.675	BB	0.0915	128.33472	22.17729	1.3972
36	12.912	BB	0.0840	49.73076	9.35484	0.5414
37	13.141	BB	0.0970	76.15899	12.52061	0.8291
38	13.400	BB	0.0704	25.84726	5.98002	0.2814
39	13.524	BB	0.0788	27.37229	5.42653	0.2980
40	13.871	BB	0.1327	88.24563	11.10517	0.9607
41	14.047	BB	0.0607	6.22959	1.70225	0.0678
42	14.242	BB	0.0958	252.66647	42.24242	2.7508
43	14.590	BB	0.1431	123.95906	13.02005	1.3495
44	14.834	BB	0.0913	410.53973	71.15630	4.4695
45	15.023	BB	0.0797	50.47131	10.20618	0.5495
46	15.303	BB	0.1142	94.85954	12.83587	1.0327
47	15.738	BB	0.1151	610.63544	78.19471	6.6480
48	16.194	BB	0.0928	80.18913	13.60635	0.8730
49	16.500	BB	0.1321	156.33078	16.87480	1.7020
50	17.064	BB	0.0930	197.06726	33.30764	2.1455
51	17.327	BB	0.1067	331.59564	49.13039	3.6101
52	17.481	BB	0.0676	10.34223	2.53401	0.1126
53	17.658	BB	0.0807	49.89624	9.91451	0.5432
54	17.874	BB	0.0906	337.69592	59.19724	3.6765
55	18.020	BB	0.0685	6.31404	1.51823	0.0687
56	18.637	BB	0.1006	343.52933	52.29137	3.7400
57	18.916	BB	0.0790	12.01330	2.46054	0.1308
58	19.111	BB	0.0888	14.07893	2.61452	0.1533
59	19.319	BB	0.1154	31.33378	4.18032	0.3411
60	19.765	BB	0.1211	619.39557	79.33156	6.7433
61	20.076	BB	0.1001	6.84601	1.04957	0.0745
62	20.368	BB	0.1220	55.57794	7.04528	0.6051
63	21.073	BB	0.1222	30.30233	4.00994	0.3299
64	21.671	BB	0.1087	53.21113	7.50975	0.5793
65	22.526	BB	0.1086	14.33296	2.12876	0.1560
66	22.829	BB	0.1005	17.07530	2.60258	0.1859
67	23.668	BB	0.1012	9.15071	1.34731	0.0996
68	23.920	BB	0.0794	6.87237	1.34954	0.0748
69	24.489	BB	0.0992	28.17593	4.25904	0.3068
70	25.080	BB	0.0789	9.81045	1.94212	0.1068
71	26.096	BB	0.0910	9.70669	1.59536	0.1057

峰 #	保留时间 [min]	类型	峰宽 [min]	峰面积 [mAU*s]	峰高 [mAU]	峰面积 %
总量 :				9185.29711	1435.31452	

信号 5: DAD1 E, Sig=300,4 Ref=off

峰 #	保留时间 [min]	类型	峰宽 [min]	峰面积 [mAU*s]	峰高 [mAU]	峰面积 %
1	2.988	BB	0.0854	41.90388	7.47404	0.3863
2	3.509	BB	0.1269	14.60819	1.65564	0.1347
3	3.840	BB	0.1039	8.41590	1.22820	0.0776
4	4.003	BB	0.1101	39.57283	5.48862	0.3648
5	4.648	BB	0.1307	11.89666	1.46608	0.1097
6	4.891	BB	0.0654	9.61166	2.36402	0.0886
7	5.180	BB	0.0707	11.12707	2.46308	0.1026
8	5.509	BB	0.1377	10.97183	1.33742	0.1011
9	5.922	BB	0.0574	15.00735	4.22924	0.1384
10	6.063	BB	0.0813	453.40686	89.26243	4.1799
11	6.272	BB	0.0805	263.07513	52.43880	2.4253
12	6.481	BB	0.0811	25.27056	4.99037	0.2330
13	6.737	BB	0.1032	16.02814	2.55360	0.1478
14	7.102	BB	0.1136	7.73523	1.10567	0.0713
15	7.519	BB	0.0783	38.66456	8.00702	0.3564
16	7.675	BB	0.0832	15.32574	3.02090	0.1413
17	7.887	BB	0.0959	54.29537	8.57408	0.5005
18	8.148	BB	0.0910	120.32958	20.34913	1.1093
19	8.458	BB	0.0992	41.65239	7.01841	0.3840
20	8.620	BB	0.0826	160.12193	30.80612	1.4761
21	8.887	BB	0.0844	44.52135	8.32425	0.4104
22	9.218	BB	0.0705	32.25499	7.45100	0.2974
23	9.468	BB	0.1257	12.24340	1.30181	0.1129
24	9.840	BB	0.1245	55.01174	7.25496	0.5071
25	10.251	BB	0.1062	452.80133	61.29847	4.1743
26	10.452	BB	0.0824	152.17128	29.37393	1.4028
27	10.756	BB	0.0882	182.86526	33.24699	1.6858
28	10.922	BB	0.0661	8.44467	2.13714	0.0779
29	11.092	BB	0.0988	39.14127	6.44626	0.3608
30	11.393	BB	0.1383	12.47187	1.18935	0.1150
31	11.675	BB	0.0946	957.06903	153.85432	8.8231
32	12.175	BB	0.1053	1794.57288	257.48019	16.5439
33	12.363	BB	0.0971	20.81227	3.23498	0.1919
34	12.912	BB	0.0942	51.61703	8.34355	0.4759
35	13.137	BB	0.0945	80.24174	13.27372	0.7397
36	13.398	BB	0.0829	72.09743	13.81905	0.6647
37	13.896	BB	0.1098	102.22849	15.33901	0.9424
38	14.242	BB	0.0934	226.11737	37.98890	2.0845
39	14.596	BB	0.1259	61.42114	7.17122	0.5662
40	14.834	BB	0.0916	679.96808	117.35806	6.2685
41	15.021	BB	0.0825	76.64197	15.28983	0.7066
42	15.301	BB	0.1087	154.45657	21.78994	1.4239

峰 #	保留时间 [min]	类型	峰宽 [min]	峰面积 [mAU*s]	峰高 [mAU]	峰面积 %
43	15.738	BB	0.1146	1038.55908	133.76144	9.5743
44	16.197	BB	0.0944	135.27739	22.40830	1.2471
45	16.501	BB	0.1340	287.24060	30.45541	2.6480
46	17.064	BB	0.0938	270.69321	45.22559	2.4955
47	17.325	BB	0.1078	531.10931	79.62675	4.8962
48	17.477	BB	0.0684	14.37534	3.32686	0.1325
49	17.657	BB	0.0795	35.42235	7.18261	0.3266
50	17.874	BB	0.0902	256.60284	45.24673	2.3656
51	18.020	BB	0.0740	6.30252	1.36225	0.0581
52	18.639	BB	0.0929	209.16818	34.41596	1.9283
53	18.913	BB	0.0724	8.44917	1.88099	0.0779
54	19.110	BB	0.0949	30.40935	5.14910	0.2803
55	19.317	BB	0.1113	21.78470	3.05153	0.2008
56	19.765	BB	0.1190	1092.01160	143.15965	10.0671
57	20.368	BB	0.1276	55.01623	6.71735	0.5072
58	21.066	BB	0.1224	42.85954	5.65340	0.3951
59	21.673	BB	0.1072	91.75453	13.18116	0.8459
60	22.829	BB	0.1056	31.49073	4.73735	0.2903
61	23.667	BB	0.0913	13.18473	2.21948	0.1215
62	23.921	BB	0.0864	12.20013	2.27921	0.1125
63	24.490	BB	0.0956	22.13557	3.50959	0.2041
64	25.078	BB	0.0816	6.56281	1.28455	0.0605
65	26.096	BB	0.0778	6.51539	1.31594	0.0601

总量 : 1.08473e4 1675.95100

信号 6: ELS1 A, ELSD Signal

峰 #	保留时间 [min]	类型	峰宽 [min]	峰面积 [mV*s]	峰高 [mV]	峰面积 %
1	1.620	BB	0.1466	5066.12354	584.36353	25.2225
2	1.755	BB	0.0528	576.99237	180.35469	2.8726
3	2.632	BB	0.1132	3566.74927	557.28680	17.7576
4	2.780	BB	0.0902	3613.70996	656.42175	17.9914
5	3.066	BB	0.0551	52.20399	15.17250	0.2599
6	3.465	BB	0.0863	80.98039	13.73431	0.4032
7	3.602	BB	0.0791	38.42830	6.35470	0.1913
8	3.964	BB	0.1115	3422.60400	467.04456	17.0400
9	4.996	BB	0.2848	90.26384	3.82457	0.4494
10	5.247	BB	0.1089	235.91086	30.45902	1.1745
11	5.785	BB	0.0602	12.72441	2.61836	0.0634
12	5.975	BB	0.0671	16.02884	3.03759	0.0798
13	6.148	BB	0.0765	187.78539	37.77498	0.9349
14	6.881	BB	0.0817	29.80251	5.28889	0.1484
15	7.262	BB	0.1063	47.86540	6.47148	0.2383
16	7.811	BB	0.1541	63.70308	5.17532	0.3172
17	8.346	BB	0.0601	8.01344	1.79474	0.0399
18	8.538	BB	0.0762	116.01964	23.87659	0.5776

峰 #	保留时间 [min]	类型	峰宽 [min]	峰面积 [mV*s]	峰高 [mV]	峰面积 %
19	8.704	BB	0.0639	13.47941	2.94447	0.0671
20	9.865	BB	0.0884	16.12541	2.59944	0.0803
21	10.336	BB	0.0984	55.57092	7.91156	0.2767
22	10.537	BB	0.0683	16.02188	3.09766	0.0798
23	10.845	BB	0.0838	24.56326	3.63737	0.1223
24	11.762	BB	0.0901	99.63641	16.35211	0.4961
25	12.226	BB	0.1173	653.92084	87.91590	3.2556
26	12.765	BB	0.0843	890.83325	165.43954	4.4351
27	12.975	BB	0.0642	10.45532	2.23153	0.0521
28	13.225	BB	0.0724	10.19264	1.72640	0.0507
29	13.944	BB	0.0885	13.52734	1.85563	0.0673
30	14.321	BB	0.0836	73.98298	12.96961	0.3683
31	14.698	BB	0.0862	18.38113	2.74058	0.0915
32	14.924	BB	0.0849	70.29726	12.64340	0.3500
33	15.383	BB	0.0768	11.97058	1.94539	0.0596
34	15.823	BB	0.1083	182.43558	24.66931	0.9083
35	16.274	BB	0.0902	41.15664	6.42607	0.2049
36	16.584	BB	0.1060	26.78996	3.15772	0.1334
37	17.144	BB	0.0950	62.03576	9.78093	0.3089
38	17.415	BB	0.1211	146.70952	18.68073	0.7304
39	17.961	BB	0.0824	125.49139	22.73145	0.6248
40	18.726	BB	0.0900	94.18520	15.46993	0.4689
41	19.500	BB	0.1222	19.41337	2.21522	0.0967
42	19.852	BB	0.1091	121.73666	16.89278	0.6061
43	21.175	BB	0.1113	16.21062	1.84962	0.0807
44	23.859	BB	0.1500	25.43079	2.07416	0.1266
45	26.204	BB	0.0803	19.29506	3.73448	0.0961

总量 : 2.00858e4 3054.74738

==

*** 报告结束 ***

File S1 BIFS ELSD
