# Supplementary material for: Clinical efficacy of Bupleurum inula flower soup for immune damage intervention in Hashimoto’s thyroiditis: A placebo-controlled randomized trial
Source: Front Pharmacol. 2022 Nov 24;13:1049618. doi: 10.3389/fphar.2022.1049618 (PMC9730284; doi:10.3389/fphar.2022.1049618)
Supplement: Supplementary file 8 [file Table1.docx]

Supplementary Material

Table S1 Laboratory test results

| **Variable** | **BIFS group** | **Control group** | **P value** |
| --- | --- | --- | --- |
| **TPOAb** [IU/ml; mean(SD)] | | | |
| Baseline | 520.23 (238.43) | 539.41 (265.33) | P>0.05 |
| After 8 weeks | 275.77 (132.98) | 441.78 (195.50) | P<0.01 |
| 24-week follow-up | 205.75 (96.20) | 352.67 (171.18) | P<0.01 |
| **TgAb** [IU/ml; mean(SD)] | | | |
| Baseline | 612.16 (350.99) | 641.21 (302.38) | P>0.05 |
| After 8 weeks | 385.92 (281.91) | 596.17 (282.26) | P<0.05 |
| 24-week follow-up | 274.38 (247.28) | 538.92 (258.12) | P<0.01 |
| **TSH** [uIU/ml; mean(SD)] | | | |
| Baseline | 17.47 (9.79) | 17.97 (10.49) | P>0.05 |
| After 8 weeks | 6.57 (3.73) | 9.63 (5.34) | P<0.05 |
| 24-week follow-up | 3.94 (1.26) | 5.82 (2.29) | P<0.01 |
| **FT3** [pmol/L; mean(SD)] | | | |
| Baseline | 3.92 (1.02) | 3.99 (1.01) | P>0.05 |
| After 8 weeks | 4.51 (0.85) | 4.45 (0.88) | P>0.05 |
| 24-week follow-up | 4.81 (0.71) | 4.75 (0.71) | P>0.05 |
| **FT4** [pmol/L; mean(SD)] | | | |
| Baseline | 9.89 (1.87) | 9.49 (2.26) | P>0.05 |
| After 8 weeks | 15.39 (2.90) | 14.04 (3.12) | P>0.05 |
| 24-week follow-up | 15.77 (2.12) | 15.36 (2.24) | P>0.05 |
| **Levothyroxine dose** [number; mean(SD)] | | | |
| Baseline | 1.68 (0.89) | 1.60 (0.83) | P>0.05 |
| After 8 weeks | 1.13 (0.57) | 1.31 (0.72) | P>0.05 |
| 24-week follow-up | 0.58 (0.43) | 1.02 (0.45) | P<0.01 |
| **Left lobe volume** [cm^3^; mean(SD)] | | | |
| Baseline | 0.53 (0.38) | 0.54 (0.35) | P>0.05 |
| After 8 weeks | 0.45 (0.25) | 0.51 (0.37) | P>0.05 |
| 24-week follow-up | 0.46 (0.25) | 0.48 (0.25) | P>0.05 |
| **Right lobe volume** [cm^3^; mean(SD)] | | | |
| Baseline | 0.54 (0.39) | 0.56 (0.43) | P>0.05 |
| After 8 weeks | 0.47 (0.24) | 0.50 (0.40) | P>0.05 |
| 24-week follow-up | 0.44 (0.23) | 0.49 (0.29) | P>0.05 |
| **Thyroid volume** [cm^3^; mean(SD)] | | | |
| Baseline | 1.10 (0.77) | 1.09 (0.75) | P>0.05 |
| After 8 weeks | 0.92 (0.47) | 1.01 (0.72) | P>0.05 |
| 24-week follow-up | 0.89 (0.46) | 0.97 (0.52) | P>0.05 |

SD, standard deviation; thyroid peroxidase antibodies (TPOAb) reference range: 0–34 IU/ml; thyroglobulin antibodies(TgAb) reference range: 0–115 IU/ml; thyroid stimulating hormone (TSH) reference range: 0.27–4.2 IU/m; free triiodothyronine (FT3) reference range: 3.1–6.8 pmol/L; free tetraiodothyronine (FT4) reference range: 12–22 pmol/L

Table S2 Depression, anxiety, and quality of life score results

| **Variable** | **BIFS group** | | **Control group** | | | **P value** |
| --- | --- | --- | --- | --- | --- | --- |
| **Depression score** [number; mean (SD)] | | | | | | |
| Baseline | | 56.33 (7.02) | | 54.08 (6.75) | P>0.05 | |
| After 8 weeks | | 47.00 (5.12) | | 51.04 (3.22) | P<0.01 | |
| 24-week follow-up | | 41.17 (5.29) | | 45.79 (5.60) | P<0.01 | |
| **Anxiety score** [number; mean (SD)] | | | | | | |
| Baseline | | 49.45 (7.02) | | 47.75 (7.80) | P>0.05 | |
| After 8 weeks | | 43.21 (4.22) | | 48.08 (2.81) | P<0.01 | |
| 24-week follow-up | | 38.79 (4.51) | | 43.08 (4.93) | P<0.01 | |
| **Physiological function score** [number; mean (SD)] | | | | | | |
| Baseline | | 55.67 (6.65) | | 54.67 (7.16) | P>0.05 | |
| After 8 weeks | | 62.08 (5.97) | | 57.96 (4.71) | P<0.05 | |
| 24-week follow-up | | 63.58 (6.76) | | 59.33 (5.95) | P<0.05 | |
| **Psychological function score** [number; mean (SD)] | | | | | | |
| Baseline | | 51.83 (8.18) | | 52.54 (7.94) | P>0.05 | |
| After 8 weeks | | 60.17 (5.94) | | 55.75 (7.09) | P<0.05 | |
| 24-week follow-up | | 63.54 (4.62) | | 57.96 (6.40) | P<0.05 | |
| **Social score** [number; mean (SD)] | | | | | | |
| Baseline | | 56.67 (10.41) | | 55.29 (11.14) | P>0.05 | |
| After 8 weeks | | 63.46 (9.48) | | 58.67 (8.74) | P>0.05 | |
| 24-week follow-up | | 66.71 (5.69) | | 63.67 (5.99) | P>0.05 | |
| **Environmental score** [number; mean (SD)] | | | | | | |
| Baseline | | 60.38 (7.59) | | 59.88 (7.01) | P>0.05 | |
| After 8 weeks | | 66.70 (5.69) | | 63.67 (5.99) | P>0.05 | |
| 24-week follow-up | | 68.17 (4.44) | | 65.96 (4.57) | P>0.05 | |

Table S3 Standard health condition test results

| **Variable** | | **BIFS group** | **Control group** | | **P value** | |
| --- | --- | --- | --- | --- | --- | --- |
| **ALT** [number; mean (SD)] | | | | | | |
| Baseline | 17.28 (1.62) | | | 14.73 (1.22) | | P>0.05 |
| After 8 weeks | 16.98 (1.67) | | | 15.94 (1.02) | | P>0.05 |
| 24-week follow-up | 16.54 (0.84) | | | 15.91 (0.99) | | P>0.05 |
| **AST** [number; mean (SD)] | | | | | | |
| Baseline | 17.74 (0.84) | | | 19.44 (1.22) | | P>0.05 |
| After 8 weeks | 18.34 (1.01) | | | 18.34 (0.53) | | P>0.05 |
| 24-week follow-up | 18.53 (0.78) | | | 18.78 (0.75) | | P>0.05 |
| **BUN** [number; mean (SD)] | | | | | | |
| Baseline | 4.02 (0.20) | | | 4.12 (0.21) | | P>0.05 |
| After 8 weeks | 4.38 (0.25) | | | 4.51 (0.21) | | P>0.05 |
| 24-week follow-up | 4.04 (0.17) | | | 4.08 (0.19) | | P>0.05 |
| **CRE** [number; mean (SD)] | | | | | | |
| Baseline | 57.92 (1.83) | | | 60.29 (1.85) | | P>0.05 |
| After 8 weeks | 57.54 (1.93) | | | 59.08 (1.33) | | P>0.05 |
| 24-week follow-up | 56.92 (1.74) | | | 57.29 (1.31) | | P>0.05 |
| **WBC** [number; mean (SD)] | | | | | | |
| Baseline | 6.52 (0.41) | | | 6.56 (0.29) | | P>0.05 |
| After 8 weeks | 6.25 (0.28) | | | 6.05 (0.26) | | P>0.05 |
| 24-week follow-up | 5.96 (0.29) | | | 6.19 (0.27) | | P>0.05 |
| **RBC** [number; mean (SD)] | | | | | | |
| Baseline | 4.43 (0.09) | | | 4.40 (0.09) | | P>0.05 |
| After 8 weeks | 4.54 (0.10) | | | 4.39 (0.10) | | P>0.05 |
| 24-week follow-up | 4.60 (0.12) | | | 4.30 (0.13) | | P>0.05 |
| **PLT** [number; mean (SD)] | | | | | | |
| Baseline | 219.92 (6.42) | | | 217.33 (7.21) | | P>0.05 |
| After 8 weeks | 226.58 (7.66) | | | 220.46 (7.99) | | P>0.05 |
| 24-week follow-up | 219.92 (6.71) | | | 216.42 (6.08) | | P>0.05 |

Alanine aminotransferase (ALT) reference range: 9–50 U/L; aspartate aminotransferase (AST) reference range: 13–40 U/L; blood urea nitrogen (BUN) reference range: 2.76–8.07 mmol/L; blood creatinine (CRE) reference range: 45–104 μmol/L; white blood cell (WBC) reference range: 4–10×10^9^/L; red blood cell (RBC) reference range: 3.8–5.1×10^12^/L; platelet (PLT) reference range: 100–300×10^9^/L
