# Supplementary material for: Clinical efficacy of Bupleurum inula flower soup for immune damage intervention in Hashimoto’s thyroiditis: A placebo-controlled randomized trial
Source: Front Pharmacol. 2022 Nov 24;13:1049618. doi: 10.3389/fphar.2022.1049618 (PMC9730284; doi:10.3389/fphar.2022.1049618)
Supplement: Supplementary file 9 [file DataSheet3.pdf]

CAMAG®
World Leader in Planar Chromatography

[illegible] 010530-2202001
R White

visionCATS
Clean, RemissionVis

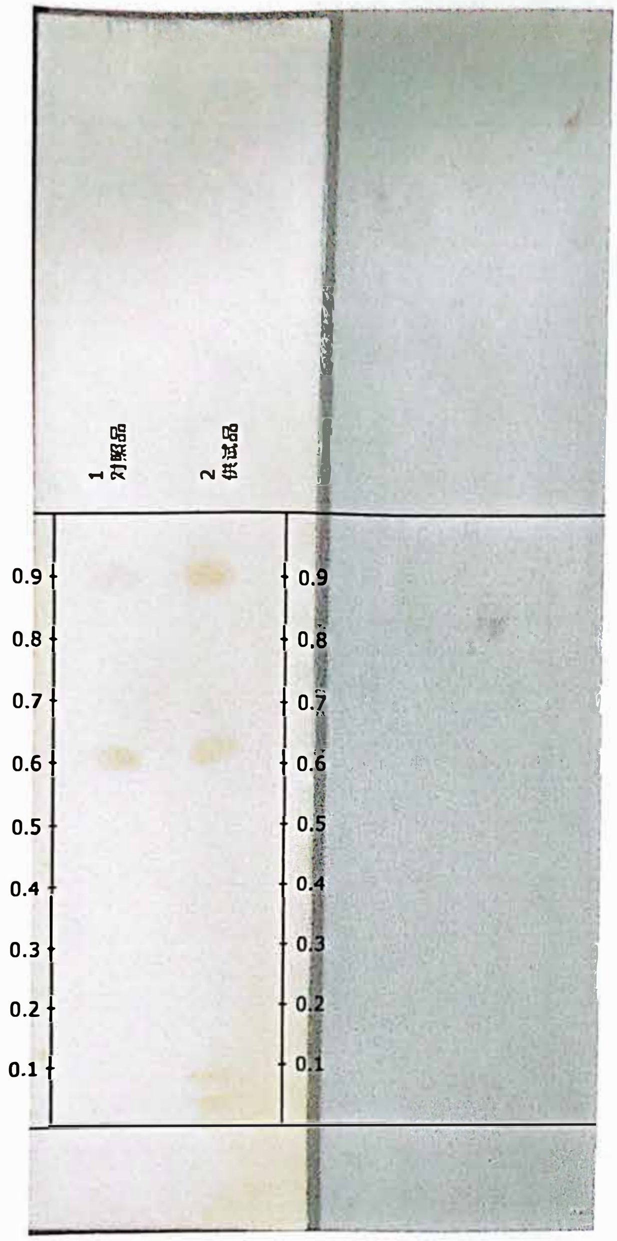

Exposure	0.124 s
Contrast	1
Normalized exposure	Disabled
Clarify	Disabled
White balance	1.00, 1.00, 1.00

Log:

03-Mar-2022 14:14:19 - 许静秋 - hpz240: File created with name '/Demo Project/2022年3月/药材/浙贝母（药材）_010530-2202001'

Steps

Plate layout

浙贝母 010530-2202001

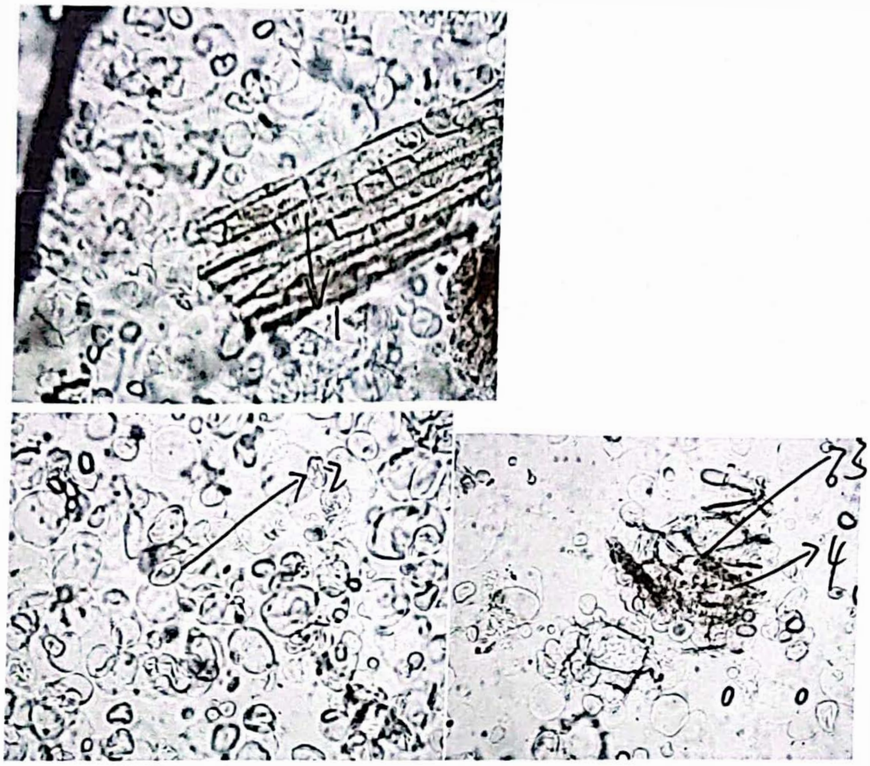

1. 导管 2.淀粉粒直径 20um3.表皮细胞 4.草酸钙结晶

Figure S7 Fritillaria
