# Supplementary material for: Clinical efficacy of Bupleurum inula flower soup for immune damage intervention in Hashimoto’s thyroiditis: A placebo-controlled randomized trial
Source: Front Pharmacol. 2022 Nov 24;13:1049618. doi: 10.3389/fphar.2022.1049618 (PMC9730284; doi:10.3389/fphar.2022.1049618)
Supplement: Supplementary file 11 [file DataSheet5.pdf]

World Leader in Planar Chromatography

[illegible] 010035-2203003 , 010035-2203004 visionCATS

Executed	01-Apr-2022 17:36:40 尹恩亲

R White

Clean, RemissionVis

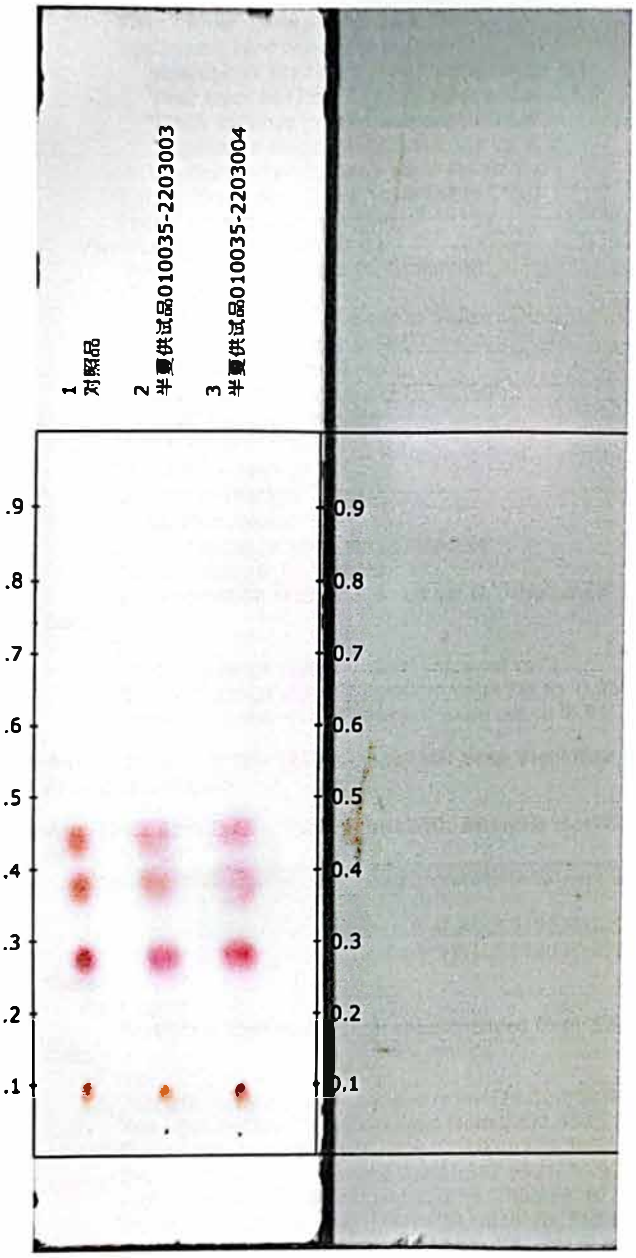

Exposure	0.129 s
Contrast	1
Normalized exposure	Disabled
Clarify	Disabled
White balance	1.00, 1.00, 1.00

Log:

01-Apr-2022 17:36:39 - 尹恩亲 - hpz240: File created with name '/Demo Project/2022年4月/药

半夏 010035-2203003

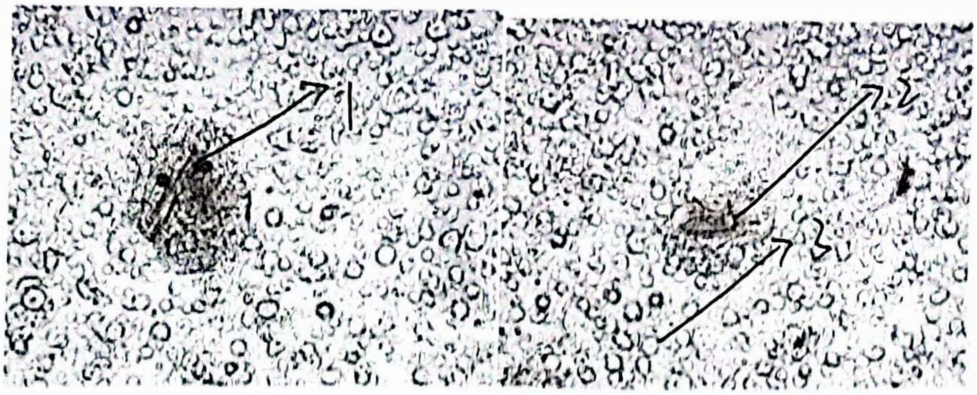

1.导管直径 20um 2.草酸钙针晶束长 90um 3.淀粉粒直径 10um

Figure S9 Pinellia
