# Supplementary material for: Clinical efficacy of Bupleurum inula flower soup for immune damage intervention in Hashimoto’s thyroiditis: A placebo-controlled randomized trial
Source: Front Pharmacol. 2022 Nov 24;13:1049618. doi: 10.3389/fphar.2022.1049618 (PMC9730284; doi:10.3389/fphar.2022.1049618)
Supplement: Supplementary file 12 [file DataSheet8.pdf]

World Leader in Planar Chromatography

[illegible] 010021-2112003 visionCATS

R White Clean, RemissionVis

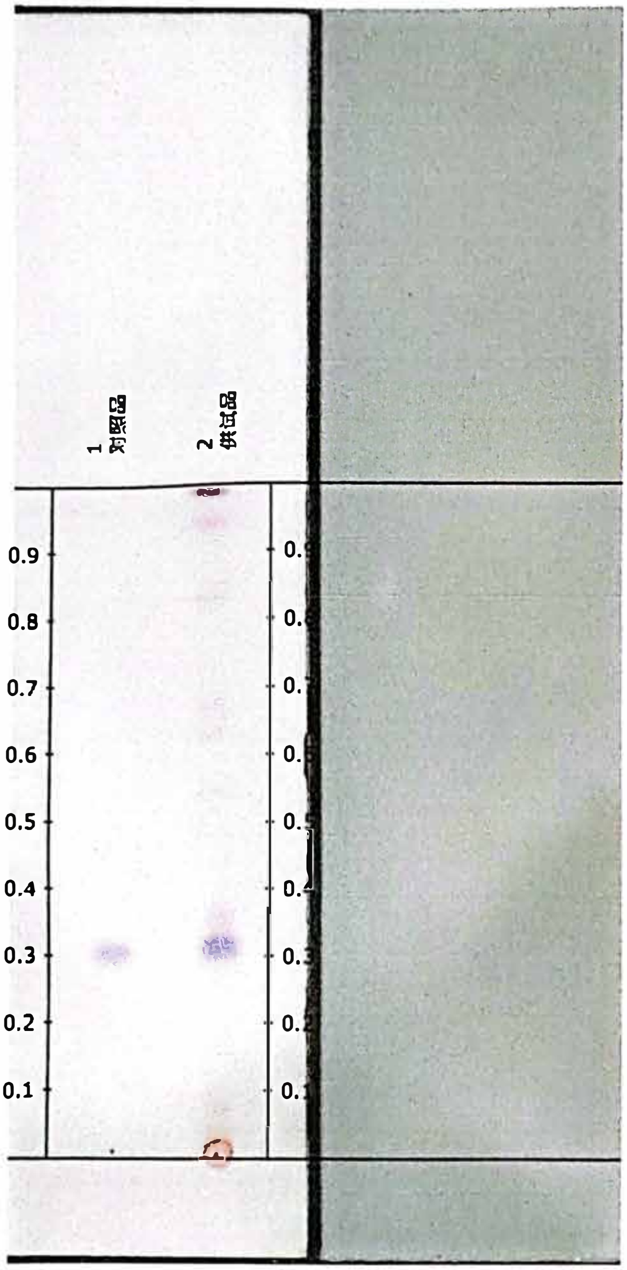

Exposure	0.167 s
Contrast	1
Normalized exposure	Disabled
Clarify	Disabled
White balance	1.00, 1.00, 1.00

Log:

23-Dec-2021 13:49:51 - 尹思亲 - hpz240: File created with name '/Demo Project/2021年12月/药材/白芍（药材）010021-2112003'

Steps

Plate layout

白芍 010021-2112003

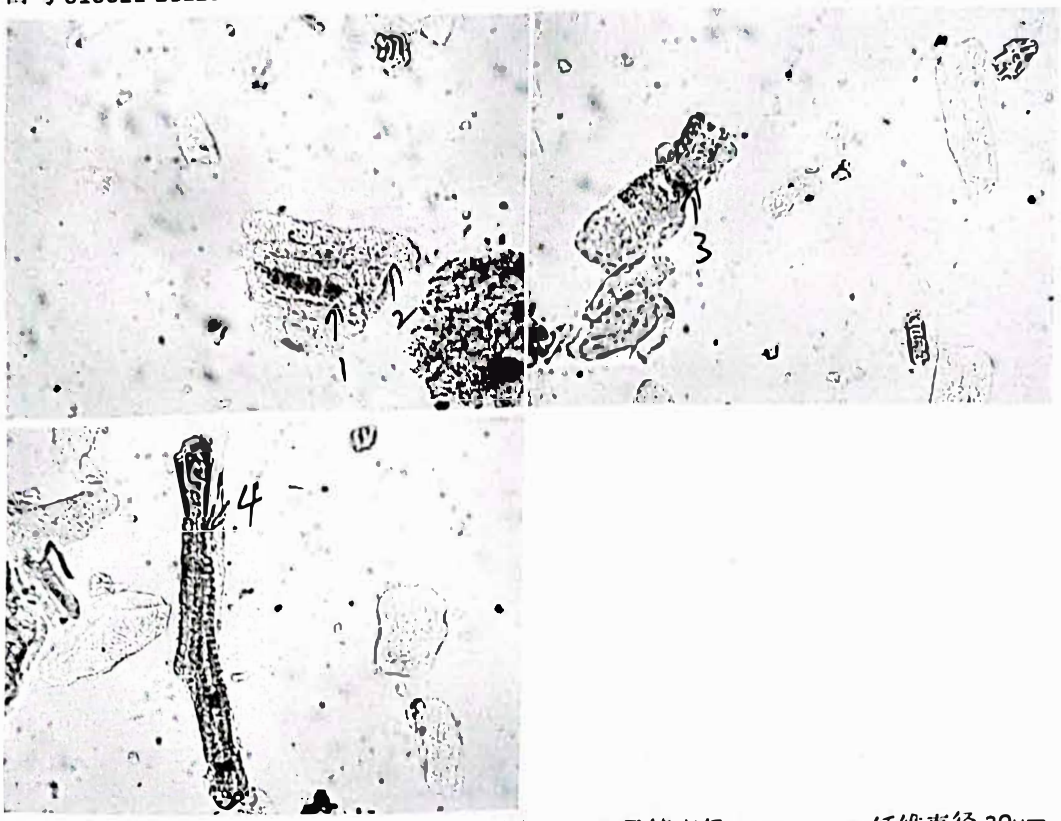

1.草酸钙簇晶直径 25um　2.糊化淀粉粒团块　3.导管直径 50um　4.纤维直径 30um

Figure S12 Roasted White Peony
