# Supplementary material for: Clinical efficacy of Bupleurum inula flower soup for immune damage intervention in Hashimoto’s thyroiditis: A placebo-controlled randomized trial
Source: Front Pharmacol. 2022 Nov 24;13:1049618. doi: 10.3389/fphar.2022.1049618 (PMC9730284; doi:10.3389/fphar.2022.1049618)
Supplement: Supplementary file 14 [file DataSheet10.pdf]

===

采集操作者	: 系统	序列行 :	2
样品操作者	: 系统		
采集仪器	: LC-ELSD	位置 :	P2-B-02
进样日期	: 2022/11/3 17:39:12	进样次数 :	1
		进样量 :	5.000 µl

来自于样品输入的不同进样量! 实际进样量: 2.000 µl

采集方法	: D:\DATA\XGY\XGY 2022-11-03 17-02-39\DAD ELSD XGY.M
上次更改	: 2022/11/3 16:48:33 : 系统
分析方法	: D:\DATA\XGY\XGY 2022-11-03 17-02-39\DAD ELSD XGY.M (序列方法)
上次更改	: 2022/11/3 17:53:19 : 系统<br>(调用后修改)

附加信息：峰被手动积分

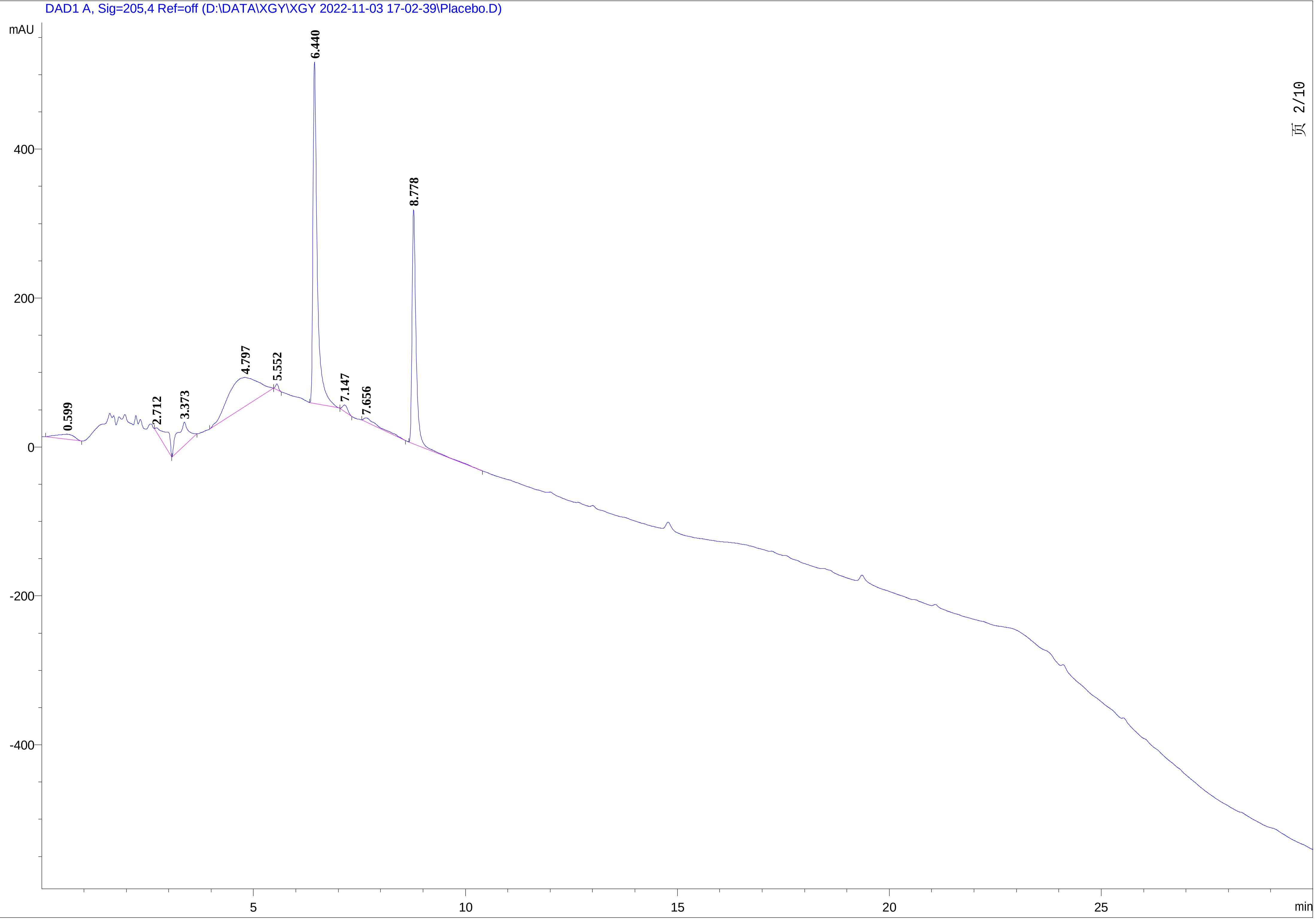
DAD1 A, Sig=205,4 Ref=off (D:\DATA\XGY\XGY 2022-11-03 17-02-39\Placebo.D)
mAU
400
200
0
-200
-400
0.599
2.712
3.373
4.797
5.552
6.440
7.147
7.656
8.778
5
10
15
20
25
min

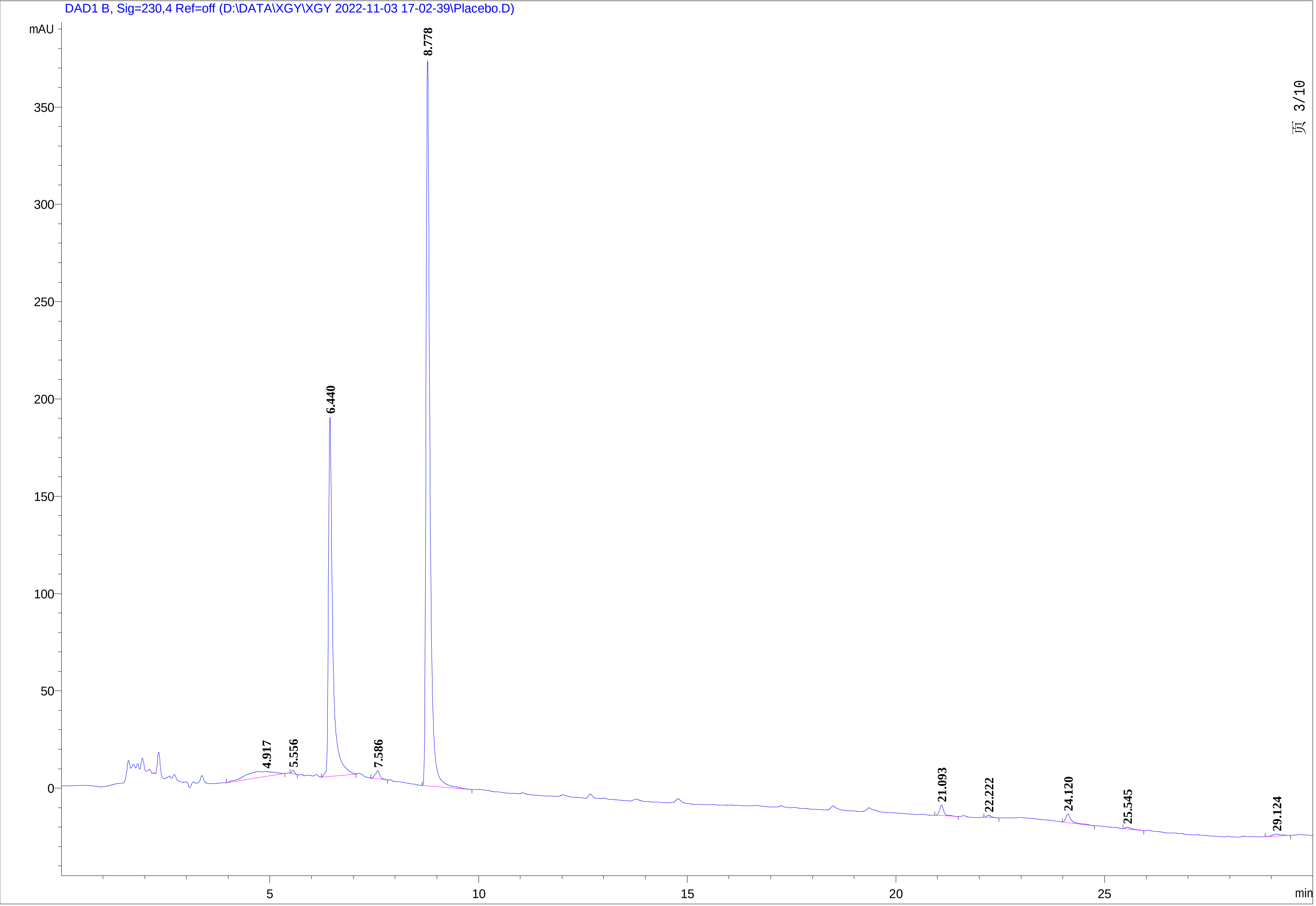
DAD1 B, Sig=230,4 Ref=off (D:\DATA\XGY\XGY 2022-11-03 17-02-39\Placebo.D)
mAU
350
300
250
200
150
100
50
0
8.778
6.440
4.917
5.556
7.586
21.093
22.222
24.120
25.545
29.124
5
10
15
20
25
min

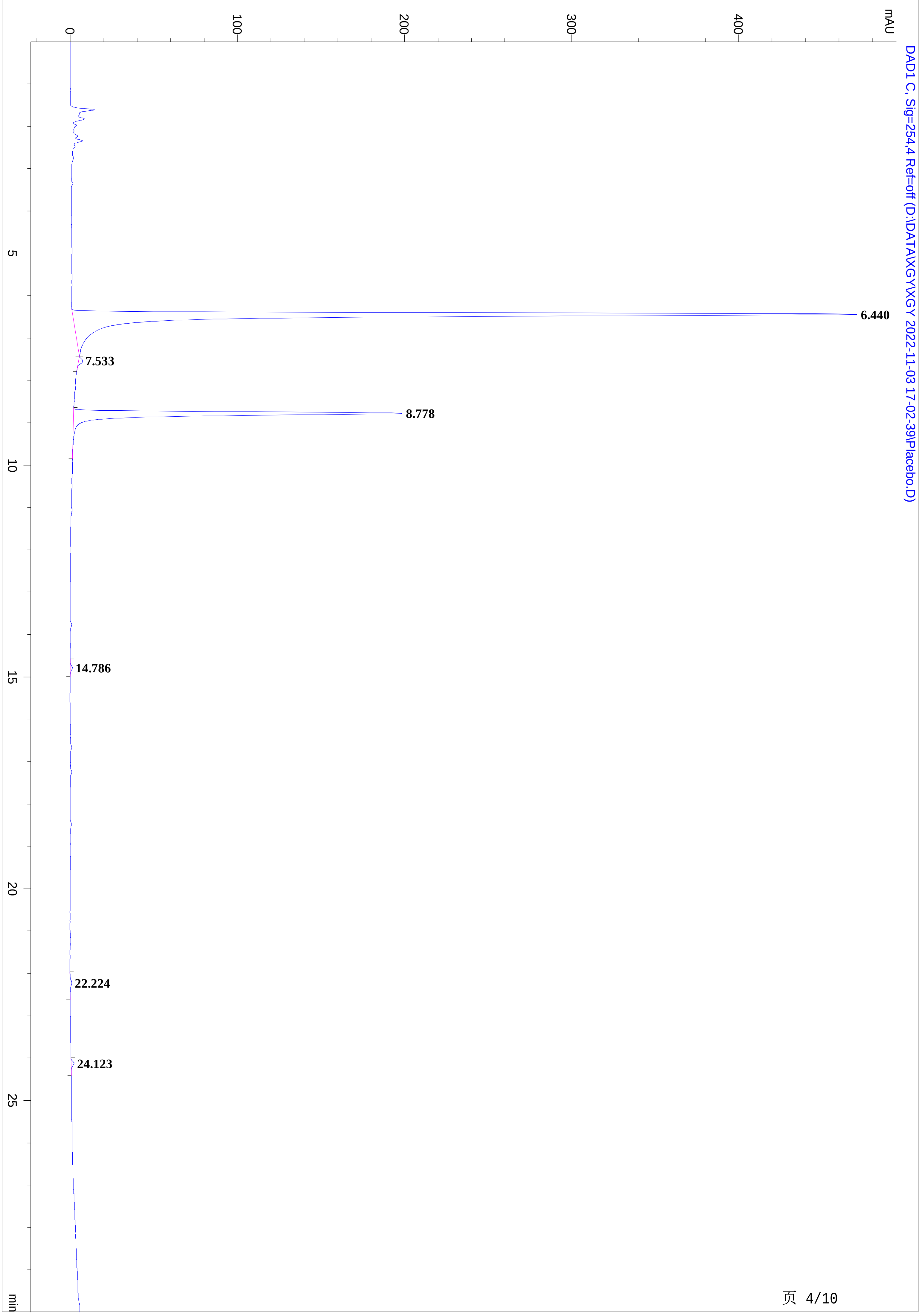
DAD1 C, Sig=254,4 Ref=off (D:\DATA\XGY\XGY 2022-11-03 17-02-39\Placebo.D)
mAU
0
100
200
300
400
min
5
10
15
20
25
6.440
7.533
8.778
14.786
22.224
24.123

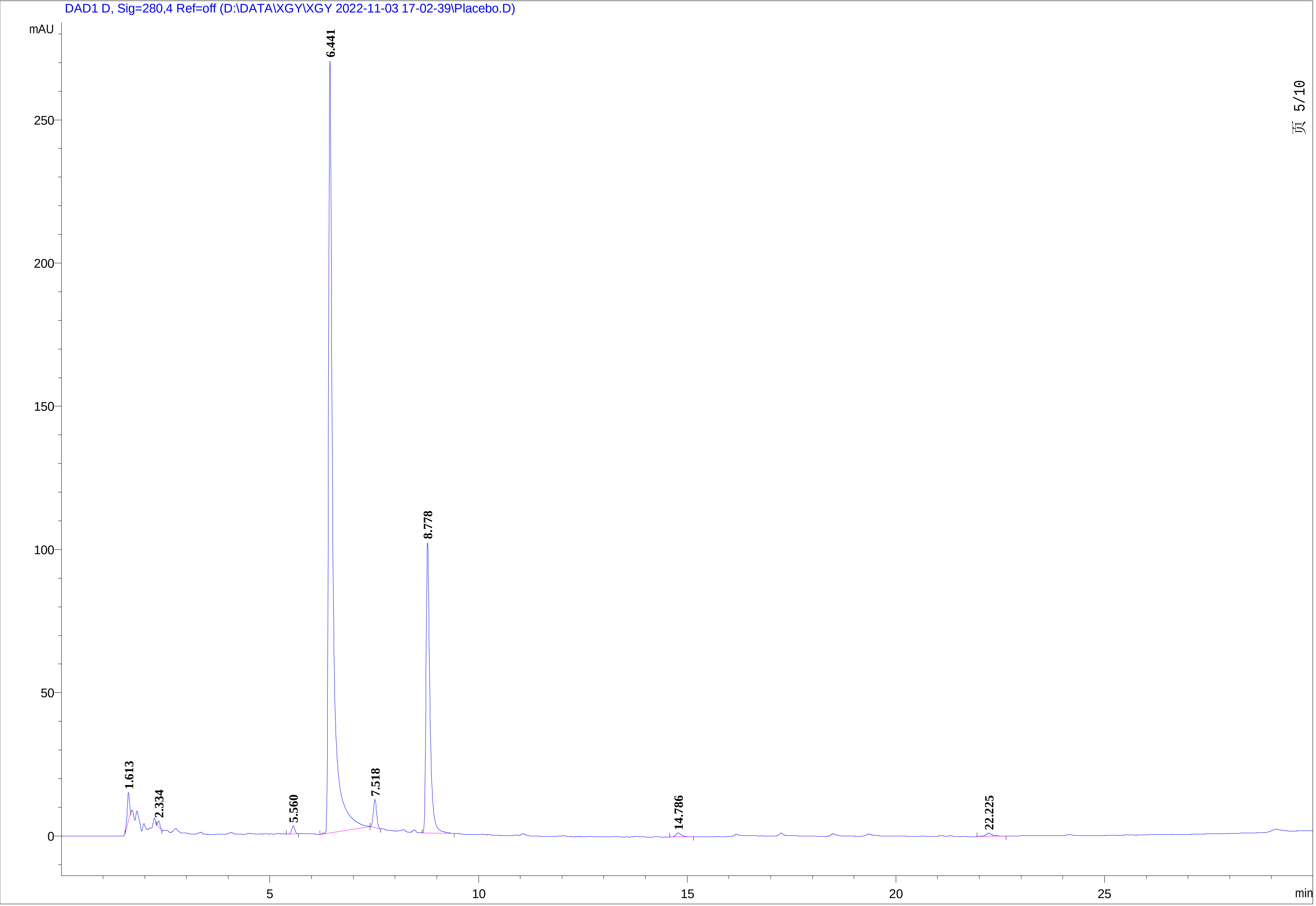
DAD1 D, Sig=280,4 Ref=off (D:\DATA\XGY\XGY 2022-11-03 17-02-39\Placebo.D)
mAU
250
200
150
100
50
0
1.613
2.334
5.560
6.441
7.518
8.778
14.786
22.225
5
10
15
20
25
min

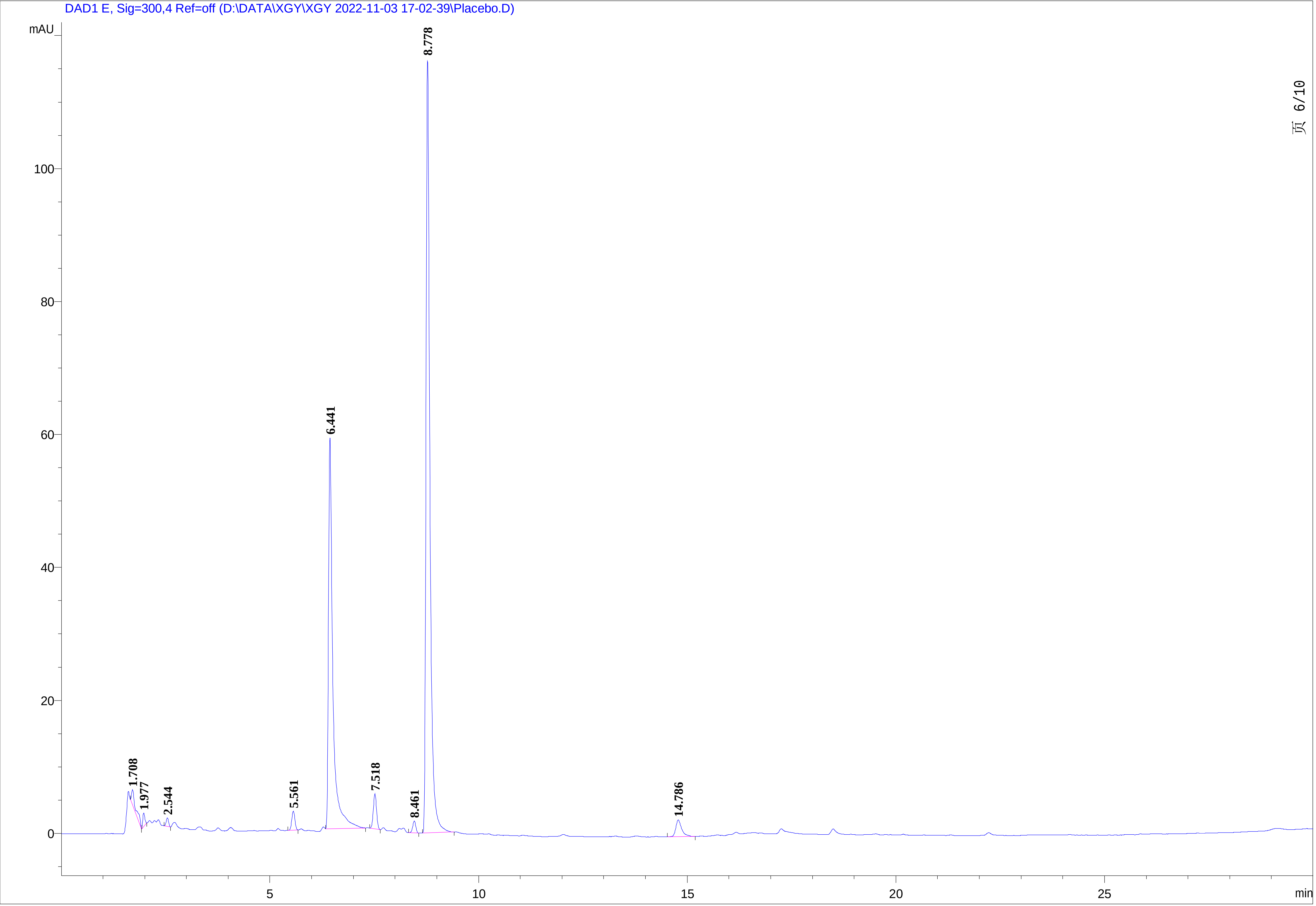
DAD1 E, Sig=300,4 Ref=off (D:\DATA\XGY\XGY 2022-11-03 17-02-39\Placebo.D)
mAU
100
80
60
40
20
0
1.708
1.977
2.544
5.561
6.441
7.518
8.461
8.778
14.786
5
10
15
20
25
min

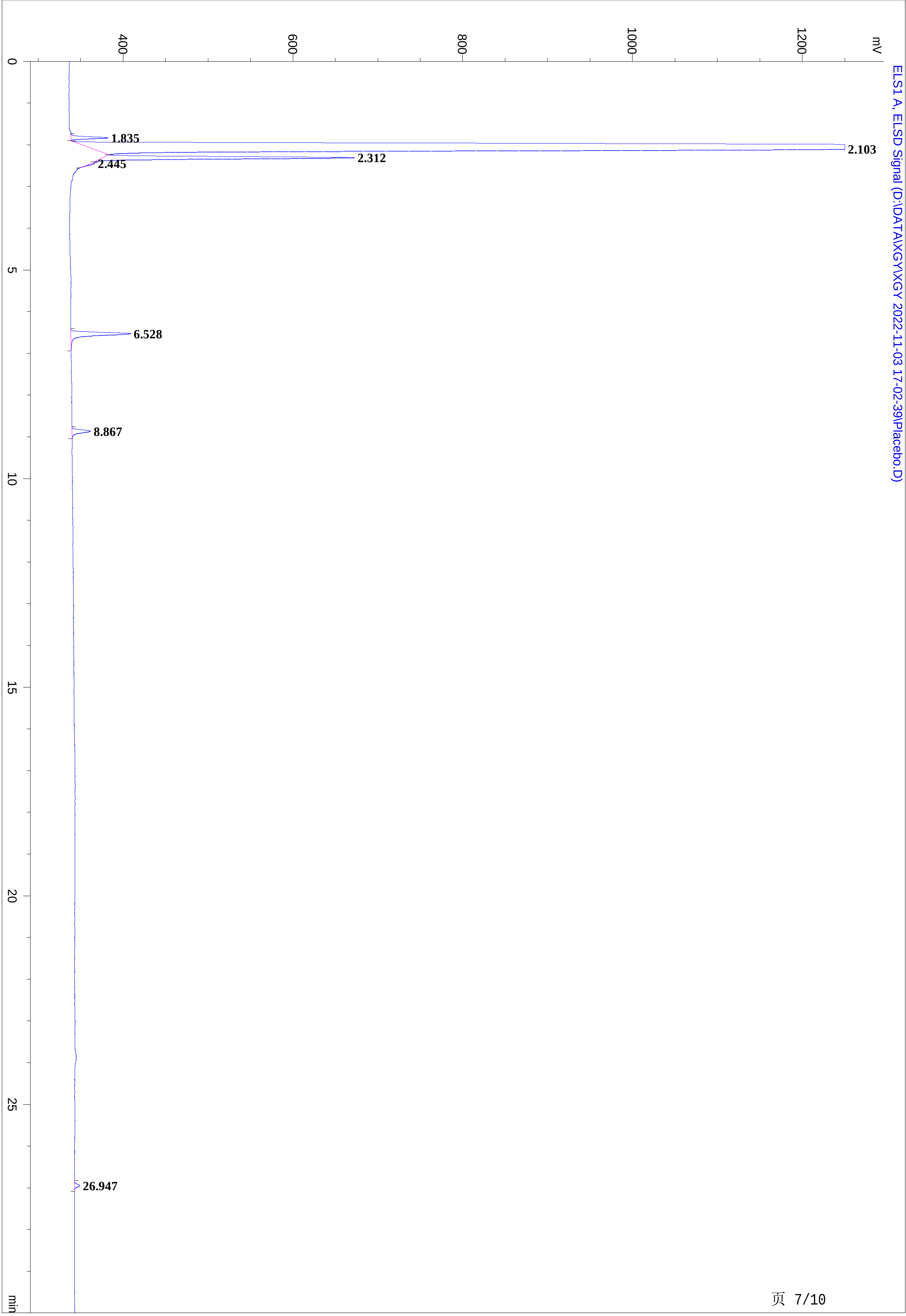
ELS1 A, ELSD Signal (D:\DATA\XGY\XGY 2022-11-03 17-02-39\Placebo.D)
mV
400
600
800
1000
1200
min
0
5
10
15
20
25
1.835
2.103
2.312
2.445
6.528
8.867
26.947

===

面积百分比报告

===

排序 : 信号
乘积因子 : 1.0000
稀释因子 : 1.0000
内标中不使用乘积因子和稀释因子

信号 1: DAD1 A, Sig=205,4 Ref=off

峰 #	保留时间 [min]	类型	峰宽 [min]	峰面积 [mAU*s]	峰高 [mAU]	峰面积 %
1	0.599	BB	0.3426	190.82060	6.77540	2.2140
2	2.712	BB	0.7221	337.67087	5.62737	3.9178
3	3.373	BB	0.2435	595.29810	31.07706	6.9069
4	4.797	BB	0.6098	1961.81360	38.94125	22.7619
5	5.552	BB	0.0723	35.66453	7.65602	0.4138
6	6.440	BB	0.1018	3218.13818	458.89496	37.3384
7	7.147	BB	0.1341	70.17633	8.34698	0.8142
8	7.656	BB	0.3330	137.91223	5.49070	1.6001
9	8.778	BB	0.0967	2071.34619	315.22043	24.0328

总量 : 8618.84063 878.03017

信号 2: DAD1 B, Sig=230,4 Ref=off

峰 #	保留时间 [min]	类型	峰宽 [min]	峰面积 [mAU*s]	峰高 [mAU]	峰面积 %
1	4.917	BB	0.6753	144.82384	2.60203	3.5649
2	5.556	BB	0.0727	7.92131	1.75267	0.1950
3	6.440	BB	0.1030	1313.41418	184.68388	32.3303
4	7.586	BB	0.1091	31.48432	4.12087	0.7750
5	8.778	BB	0.0965	2450.33862	373.56699	60.3162
6	21.093	BB	0.1081	39.69612	5.50814	0.9771
7	22.222	BB	0.1199	8.02660	1.01928	0.1976
8	24.120	BB	0.1326	39.81590	4.35578	0.9801
9	25.545	BB	0.1384	9.93736	1.03103	0.2446
10	29.124	BB	0.2347	17.02752	1.03094	0.4191

总量 : 4062.48578 579.67160

信号 3: DAD1 C, Sig=254,4 Ref=off

峰 #	保留时间 [min]	类型	峰宽 [min]	峰面积 [mAU*s]	峰高 [mAU]	峰面积 %
1	6.440	BB	0.1069	3499.43945	469.82562	72.2299
2	7.533	BB	0.1372	19.57948	2.35116	0.4041
3	8.778	BB	0.0962	1287.59631	197.13515	26.5765
4	14.786	BB	0.1298	11.46586	1.34046	0.2367
5	22.224	BB	0.1596	12.20280	1.08197	0.2519
6	24.123	BB	0.1204	14.57691	1.80161	0.3009

总量 : 4844.86083 673.53596

信号 4: DAD1 D, Sig=280,4 Ref=off

峰 #	保留时间 [min]	类型	峰宽 [min]	峰面积 [mAU*s]	峰高 [mAU]	峰面积 %
1	1.613	BB	0.0576	36.64767	9.80793	1.3132
2	2.334	BB	0.0582	7.56934	2.09187	0.2712
3	5.560	BB	0.0796	14.36916	2.81159	0.5149
4	6.441	BB	0.1065	1998.10437	269.62103	71.5999
5	7.518	BB	0.0815	51.69260	9.80153	1.8523
6	8.778	BB	0.0954	656.20306	101.51998	23.5143
7	14.786	BB	0.1468	13.60130	1.38208	0.4874
8	22.225	BB	0.1561	12.46589	1.13529	0.4467

总量 : 2790.65340 398.17130

信号 5: DAD1 E, Sig=300,4 Ref=off

峰 #	保留时间 [min]	类型	峰宽 [min]	峰面积 [mAU*s]	峰高 [mAU]	峰面积 %
1	1.708	BB	0.1009	17.00579	2.33677	1.3200
2	1.977	BB	0.0548	7.24640	2.07188	0.5625
3	2.544	BB	0.0630	5.13247	1.27383	0.3984
4	5.561	BB	0.0821	14.81147	2.87477	1.1496
5	6.441	BB	0.1046	426.52548	58.82265	33.1063
6	7.518	BB	0.0818	27.89631	5.27001	2.1653
7	8.461	BB	0.0789	8.94756	1.77361	0.6945
8	8.778	BB	0.0957	755.81384	116.50497	58.6652
9	14.786	BB	0.1448	24.97177	2.53749	1.9383

总量 : 1288.35110 193.46596

信号 6: ELS1 A, ELSD Signal

峰 #	保留时间 [min]	类型	峰宽 [min]	峰面积 [mV*s]	峰高 [mV]	峰面积 %
1	1.835	BB	0.0561	151.48112	43.56853	1.2553
2	2.103	BB	0.1986	1.02456e4	884.98657	84.9028
3	2.312	BB	0.0605	1129.71436	296.27988	9.3616
4	2.445	BB	0.0617	18.47926	3.67172	0.1531
5	6.528	BB	0.0820	378.02597	70.58166	3.1326
6	8.867	BB	0.0792	112.29459	21.77238	0.9306
7	26.947	BB	0.0747	31.86050	6.44021	0.2640
总量 :				1.20675e4	1327.30094	

===

*** 报告结束 ***

File S2 Placebo ELSD
